# SIRE 2.0: a novel method for estimating polygenic host effects underlying infectious disease transmission, and analytical expressions for prediction accuracies

**DOI:** 10.1186/s12711-025-00956-4

**Published:** 2025-04-01

**Authors:** Christopher M. Pooley, Glenn Marion, Jamie Prentice, Ricardo Pong-Wong, Stephen C. Bishop, Andrea Doeschl-Wilson

**Affiliations:** 1 Biomathematics and Statistics Scotland, James Clerk Maxwell Building, The King’s Buildings, Peter Guthrie Tait Road, Edinburgh, EH9 3FD UK; 2 The Roslin Institute, The University of Edinburgh, Easter Bush Campus, Midlothian, EH25 9RG UK

## Abstract

**Background:**

Genetic selection of individuals that are less susceptible to infection, less infectious once infected, and recover faster, offers an effective and long-lasting solution to reduce the incidence and impact of infectious diseases in farmed animals. However, computational methods for simultaneously estimating genetic parameters for host susceptibility, infectivity and recoverability from real-word data have been lacking. Our previously developed methodology and software tool SIRE 1.0 (Susceptibility, Infectivity and Recoverability Estimator) allows estimation of host genetic effects of a single nucleotide polymorphism (SNP), or other fixed effects (e.g*.* breed, vaccination status), for these three host traits using individual disease data typically available from field studies and challenge experiments. SIRE 1.0, however, lacks the capability to estimate genetic parameters for these traits in the likely case of underlying polygenic control.

**Results:**

This paper introduces novel Bayesian methodology and a new software tool SIRE 2.0 for estimating polygenic contributions (i.e. variance components and additive genetic effects) for host susceptibility, infectivity and recoverability from temporal epidemic data, assuming that pedigree or genomic relationships are known. Analytical expressions for prediction accuracies (PAs) for these traits are derived for simplified scenarios, revealing their dependence on genetic and phenotypic variances, and the distribution of related individuals within and between contact groups. PAs for infectivity are found to be critically dependent on the size of contact groups. Validation of the methodology with data from simulated epidemics demonstrates good agreement between numerically generated PAs and analytical predictions. Genetic correlations between infectivity and other traits substantially increase trait PAs. Incomplete data (e.g. time censored or infrequent sampling) generally yield only small reductions in PAs, except for when infection times are completely unknown, which results in a substantial reduction.

**Conclusions:**

The method presented can estimate genetic parameters for host susceptibility, infectivity and recoverability from individual disease records. The freely available SIRE 2.0 software provides a valuable extension to SIRE 1.0 for estimating host polygenic effects underlying infectious disease transmission. This tool will open up new possibilities for analysis and quantification of genetic determinates of disease dynamics.

**Supplementary Information:**

The online version contains supplementary material available at 10.1186/s12711-025-00956-4.

## Background

Infectious diseases in animals, plants and humans cause huge economic losses, threats to global health and food security, and have a large carbon footprint [1–3]. Many infectious pathogens infect both animals and humans, implying that infections in farmed animals, which are commonly reared in confined environments that foster pathogen transmission, also pose significant threats to human health [4]. Hence, more than ever, reducing pathogen transmission is paramount for healthy populations and farming systems, for national and international food security and for alleviating poverty in developing countries [5]. To supplement existing strategies for disease control (e.g. vaccination, improved biosecurity, pharmaceuticals), genetic control strategies have long been considered a promising avenue with long-lasting benefits [6–8]. These are rapidly gaining momentum through breakthroughs in genomic technologies leading to a deepening scientific understanding in disease genetics [9–12].

Until recently, genetic disease control has mostly focused on increasing individual disease resistance or resilience [7, 13]. However, individuals are rarely fully resistant to infection, and the impact of these genetic improvement strategies on pathogen transmission, and thus infectious disease prevalence at the population level, is still poorly understood [12, 14–16]. Epidemiological theory points to three distinct (but potentially correlated) host traits affecting transmission dynamics: susceptibility (propensity to become infected), infectivity (propensity to pass on infection to others) and recoverability (propensity to recover or die), affecting how long hosts are infectious for [16, 17]. Recent research indicates that all three of these epidemiological host traits may harbour substantial genetic (co-)variation [18–21], and so could be targeted for more effective disease control [12, 16, 20, 22, 23]. However, what currently blocks practical implementation is the absence of suitable methods and computational tools for quantitative genetic analyses of these traits.

Genetic analysis using existing quantitative genetics methods, such as genome-wide association studies (GWAS) [24] or genomic prediction methods [25, 26], are hampered by the fact that the susceptibility, infectivity and recoverability of individuals cannot be measured directly but must be inferred from available disease data. Moreover, an individual’s disease status is determined not only by its own susceptibility and recoverability, but also by the infectivity of infected individuals it comes into contact with. This complex dynamic interdependence between underlying unobservable epidemiological and observable disease traits poses challenges for existing genetic evaluation approaches.

Several methods have been proposed to simultaneously estimate genetic effects for host susceptibility and infectivity from epidemiological data. One has been to incorporate infectivity as an indirect genetic effect (IGE) on individuals’ observed infection status in a generalized linear mixed model (GLMM) framework, using existing software tools, such as ASReml [27], for variance component estimation and genetic evaluations. This approach is exemplified by [28, 29] who investigate the effects of single genes or SNPs for host susceptibility and infectivity, and by [21, 30] who treat these traits as polygenic. Whilst appealing, these approaches rely on numerous assumptions and approximations, which could potentially generate bias and compromise prediction accuracies and thus genetic gain. Moreover, they lack flexibility with regard to the types of diseases or disease phenotypes that can be used, and require precise information about individuals’ infection status at frequent time intervals [29, 30], which are rarely available in practice.

Bayesian inference, on the other hand, has proven a powerful approach for dealing with missing information, being flexible in terms of model structure, and for accommodating different sources of uncertainty inherent in data [31, 32]. This is exemplified by our previously developed Markov chain Monte Carlo (MCMC) approaches for estimating genotype effects or genetic parameters for host susceptibility and infectivity [33, 34]. However, further improvements of these methods, in terms of computational efficiency and versatility to accommodate more complex infection dynamics and various sources of underlying genetic and non-genetic variation, are needed to be applicable to real-word scenarios.

The first SIRE (Susceptibility, Infectivity and Recoverability Estimator) [17] software tool (SIRE 1.0) is designed to fill some of these gaps. It allows for simultaneous estimation of the genetic effect of a single nucleotide polymorphism (SNP), or other fixed genetic or non-genetic effects (e.g*.* breed, sex, vaccination status) on the three unobservable host traits from a wide range of individuals’ disease records. The data requirements and optimal experimental design for estimating such effects for all three epidemiological traits are specified in a follow-up study with complementary online precision calculate software (SIRE-PC), pertaining to a range of experimental designs and sampling methods [35].

Whilst some diseases are known to be regulated by a single gene of large effect (e.g. IPN in salmon [34, 36]), it is expected that in most cases the epidemiological host traits are polygenic in nature [37, 38]. This paper introduces the methodology and software tool SIRE 2.0 that extends the capability of SIRE 1.0 [17] to allow for estimation of polygenic contributions to susceptibility, infectivity and recoverability. These contributions not only take into account genetic relationships between individuals through a pedigree/genomic relationship matrix, but also correlations between the three epidemiological host traits themselves. Furthermore, we derive analytical expression for prediction accuracies (PAs) for these traits for simplified scenarios. These prove useful in validating the new methodology and inference software, but also in providing relevant insights for future experimental or field study designs.

The methodology is first illustrated and validated using an exemplar scenario that employs a somewhat arbitrary, but plausible, parameter set and simulated data (representative of what might be available from experimental or field studies on farmed animals). This is followed by a systematic simulation study aimed at understanding variation in prediction accuracies across parameter space, population structure and contact group structure.

## Methods

### Data structure and assumptions

The inference methodology introduced here applies to individual-level disease data originating from epidemics in one or more closed contact groups. In the ideal case such data are individuals’ infection or recovery or death times, but SIRE 2.0 accommodates a range of more realistic data scenarios that can be generated by controlled disease transmission experiments or less controlled field studies. Such data may range from records of individual recovery or mortality times, or periodic checks of individual infection status based on in-vivo diagnostic test results (a more detailed description of the diverse data scenarios is given in [17]). SIRE 2.0 can also accommodate time censored data. To estimate genetic effects, it is necessary for pedigree or genomic relationship information of all individuals involved in the epidemics to be available.

It is assumed that epidemics in each contact group are initiated by one or a few infectious animals which transmit infectious pathogens to susceptible individuals in the same contact group through direct contact. These initially infected individuals may be artificially infected (e.g. in the case of experiments) or acquire infection by some unknown means (as is more usual for field studies). Contact groups refer to groups of individuals sharing the same environment such as a pasture, pen, cage or pond. For simplicity it is assumed that, throughout the observation period, groups are closed, i.e*.* no births, migrations, or transmission of infectious pathogens between groups. This assumption generally holds for experimental studies, and also for most common field situations, where a movement ban is imposed after disease notification, or outbreaks are sufficiently distinct in time and/or space.

It is assumed that the infection dynamics within each contact group can be adequately represented by an epidemiological SIR (or SI) model, in which individuals are classified as being either susceptible to infection (S), infected and infectious (I), or recovered/removed/dead (R) [1]. SIR models are commonly used to model the transmission dynamics for a wide range of infectious diseases affecting animals, humans and plants [39].

### The genetic-epidemiological model

SIRE 2.0 embeds a stochastic SIR model with genetic heterogeneity in susceptibility, infectivity and recoverability.

Under the standard SIR model for homogeneous populations, the time-dependent force of infection for a susceptible individual (i.e. the probability per unit time of becoming infected) is given by *λ*(t) = *βI*(t), which is the product of an average transmission rate *β* and *I*(t), the number of infected individuals at time *t*. The simplest SIR model assumes individuals recover on average at rate *γ*. In reality, however, most diseases do not follow this recovery profile. A more accurate representation, as used here, is to introduce a gamma distributed infectious duration with mean *w* = *1*/*γ* and shape parameter *k* (where the shape accounts for variation around the mean corresponding to the particular pathogen/host under investigation).^1^ Mathematically this can be written as $\delta t_{p} \sim \Gamma \left( {w_{p} ,k} \right)$, where ${\delta t}_{p}$ is the infection duration for individual *p* (note, the quantity ${\delta t}_{p}$ will crop up later in the model likelihood Eq. (4), which shows how Bayesian inference can inform both the means and shape of this distribution).

Incorporating individual-based variation in susceptibility, infectivity and recoverability into this basic SIR model, the force of infection *λ*_*j*_(t) for an individual* j* in contact group *z*, and the infectious duration distribution mean* w*_*j*_ (from which the infection duration is sampled) are given by

1 $$\lambda_{j} (t) = \beta e^{{c_{z} }} e^{{g_{j} }} \sum\nolimits_{i} {e^{{f_{i} }} } ,\quad \quad w_{j} = (\gamma e^{{r_{j} }} )^{ - 1}$$

(for a formal derivation see [33, 34] and [17]). The sum *i* goes over all infected individuals sharing the same contact group *z* as *j* at time *t*. Here *β* refers to a population-level average transmission rate for the disease, and *g*_*j*_ and *r*_*j*_ represent fractional deviations^2^ in the susceptibility and recoverability of individual *j* compared to a population-wide reference. The fractional deviation in infectivity for individual *i* is denoted *f*_*i*_. Finally, **c** is a vector of random effects (with standard deviation σ_c_ and each element *c*_*z*_ acting on contact group *z*) which accounts for group-specific factors that influence the overall speed of an epidemic in one contact group relative to another (e.g*.* animals kept under different management conditions, climatic or other environmental differences). The time taken for individual *j* to recover after being infected is taken to be sampled from a gamma distribution with mean *w*_*j*_ and shape parameter *k*. For reference, a list of all model parameters is shown in Table 1.

**Table 1:** Parameters

Type	Parameter	Description
Population-wide epidemiological parameters	*β*	Population average transmission rate
	*γ*	Population average recovery rate
	*k*	Shape parameter that characterises the dispersion in infection durations
Individual-based epidemiological traits	*λ*_*j*_	Force of infection (probability per unit time for individual *j* to become infected)
	*w*_*j*_	Mean of infection duration distribution for individual *j*
	*g*_*j*_, *f*_*j*_, *r*_*j*_	Fractional deviation in susceptibility, infectivity and recoverability of individual *j*
Genetic effects	**a**_**g**_, **a**_**f**_, **a**_**r**_	Additive genetic contributions to **g**, **f**, **r**
	**A**	Relationship matrix (from pedigree or genomic) which characterises trait correlations between individuals
	**Ω**	3 × 3 covariance matrix that characterises genetic variances and correlations between traits
Environmental effects	**ε**_**g**_, **ε**_**f**_**, ε**_**r**_	Environmental contributions to **g**, **f**, **r**
	**I**	The identity matrix
	**Ψ**	Covariance matrix of residual contributions
Fixed effects	**b**_**f**_, **b**_**f**_, **b**_**r**_	Vectors of fixed effects for the three traits
	**X**	The design matrix for fixed effects
Group effects	*c*_*z*_	Contact group effects (accounts for differences in transmission rate for different contact groups)
	σ_c_	Standard deviation in contact group effects
Bayesian model	*θ*	Set of all model parameters
	*ξ*	Set of all event data (infection and recovery times)
Derived genetic parameters	**Θ** = **Ω + Ψ**	Phenotypic covariance matrix
	${h}_{g}^{2}={\Omega }_{gg}/{\Theta }_{gg}$	Heritability for susceptibility
	${h}_{f}^{2}={\Omega }_{ff}/{\Theta }_{ff}$	Heritability for infectivity
	${h}_{r}^{2}={\Omega }_{rr}/{\Theta }_{rr}$	Heritability for recoverability
Prediction accuracies	${\alpha }_{g,n},$ ${\alpha }_{f,n},{\alpha }_{r,n}$	Prediction accuracies for susceptibility, infectivity and recoverability for individual *n* (found from the correlation between the posterior mean of the additive genetic values *a*_*g,n*_, *a*_*f,n*_,* a*_*r,n*_ and their true values across multiple datasets)
	${\alpha }_{g,SIRE},$ ${\alpha }_{f,SIRE}, {\alpha }_{r,SIRE}$	Prediction accuracies for susceptibility, infectivity and recoverability for sires
Contact groups	*Z*	Number of contact groups
	*N*	Number of individuals per contact group,
	*P*	Number of offspring per sire

This table provides a list of key parameters and quantities used in this paper, categorised by type and briefly described. Bold quantities represent vectors or matrices

The latent phenotypes, i.e. the individual-based fractional deviations in susceptibility, infectivity and recoverability, are parameterised using a linear mixed model with additive genetic effects:

2 $$\begin{gathered} {\mathbf{g}} = {\mathbf{X}}{\mathbf{b}}_{{\mathbf{g}}} + {\mathbf{a}}_{{\mathbf{g}}} + {{\varvec{\upvarepsilon}}}_{{\mathbf{g}}} , \\ {\mathbf{f}} = {\mathbf{X}}{\mathbf{b}}_{{\mathbf{f}}} + {\mathbf{a}}_{f} + {{\varvec{\upvarepsilon}}}_{{\mathbf{f}}} , \\ {\mathbf{r}} = {\mathbf{X}}{\mathbf{b}}_{{\mathbf{r}}} + {\mathbf{a}}_{r} + {{\varvec{\upvarepsilon}}}_{{\mathbf{r}}} . \\ \end{gathered}$$

Here **g**, **f** and **r** are vectors (containing information for each of the individuals) that are decomposed into fixed effects **b**_**g**_, **b**_**f**_ and **b**_**r**_ (e.g*.* to account for sex differences in the traits or vaccination status), where **X** is the corresponding design matrix, additive genetic contributions **a** = (**a**_**g**_, **a**_**f**_, **a**_**r**_) and residual contributions **ε** = (**ε**_**g**_, **ε**_**f**_, **ε**_**r**_).

The additive genetic contributions **a** in Eq. (2) account for the relationship in trait values between different individuals. These are taken to be multivariate-normally distributed with zero mean and covariance matrix defined by the Kronecker product **A** ⊗ **Ω**. Here **A** is the genetic relationship matrix (calculated using pedigree information or high-density genotyping, or a combination of both) and** Ω** is a 3 × 3 covariance matrix that characterises potential genetic correlations between the three traits.

The residual contributions **ε** in Eq. (2), subsequently referred to as “environmental” effects, account for all other variation. These are also multivariate normal with zero mean and covariance matrix **I** ⊗ **Ψ**, where **I** is the identity matrix, reflecting the fact that residuals are assumed to be uncorrelated between individuals, and **Ψ** is a 3 × 3 covariance matrix that characterises environmental correlations between traits.

The phenotypic variance is defined as the sum of the genetic and environmental contributions **Θ** = **Ω + Ψ**.

As shown in Additional file 1 [40], the model in Eq. (2) can easily be extended to incorporate SNP effects, following the methods of the original SIRE 1.0 paper [17], and so estimation of these isn’t investigated here.

### Bayesian inference methodology

Bayesian inference is used to estimate the genetic-epidemiological model parameters based on potentially uncertain individual disease records, as described in the ‘Data structure and assumptions’ section above. The model contains the following set of parameters: *θ* = {*β*, *γ*, *k*, **b**_**g**_, **b**_**f**_, **b**_**r**_, **a**_**g**_, **a**_**f**_, **a**_**r**_, **Ω**, **ε**_**g**_,** ε**_**f**_, **ε**_**r**_, **Ψ**, **c**, σ_c_} (Table 1). We denote the complete set of infection and recovery event times for all individuals over the observed duration of the epidemic as *ξ*. Typically *ξ* is not precisely known, and so we consider the general case in which *ξ* represents a set of latent model variables. The nature of the actual observed data will be problem dependant. For example, in some instances recovery or removal (e.g. due to death) times will be precisely known, but infection times completely unknown. In other cases, infection and recovery times will both be unknown, but results from disease diagnostic tests provide uncertain information regarding individuals’ infection status at particular points in time.

Application of Bayes’ theorem implies that the posterior probability distribution for model parameters and latent variables given the data *y* is

3 $$\pi (\theta ,\xi |y) \propto \pi \left( {y|\xi } \right)L(\xi |\theta )\pi (\theta ),$$

with the following terms:

#### Observation model π(y|ξ)

This is the probability of the data *y* given a set of event times *ξ* (i.e*.* individuals’ infection and/or recovery times). The expression for the observation model depends on the nature of the data being observed (see description of possible data scenarios in the ‘Data structure and assumptions’ section above). In many cases this model simply uses the values zero or constant depending on whether *ξ* is consistent with *y* or not. For example, an infected individual identified by a perfect disease diagnostic would only be consistent with *ξ* containing an infection event on that individual *prior* to the time of the test and a recovery event *after* the time of the test. In the case of imperfect disease diagnostic test results, the probability in the observation model includes the sensitivity and specificity of the test to account for this uncertainty in the data. In summary, the observation model depends on the data collection process and constrains the possible event sequences *ξ*, which in turn informs the model parameters *θ*.

#### Latent process likelihood L(ξ|θ)

This is the probability of *ξ* being sampled from the model given parameters *θ*. It is derived from the genetic-epidemiological model following [41, 42] and given by

4 $$L(\xi |\theta ) = \prod\nolimits_{z} {\left[ {\left( {\prod\nolimits_{j \in z} {\lambda_{j} } } \right)\left( {\prod\nolimits_{{e \in E_{z} }} {e^{{ - \Lambda_{z} (t_{e} ) \times (t_{e} - t_{e - 1} )}} } } \right)} \right]} \left( {\prod\nolimits_{p} {F_{\Gamma } (\delta t_{p} |w_{p} ,k)} } \right).$$

The functional dependence of *L*(*ξ*|*θ*) on parameters *θ* is expressed in terms of the force of infections *λ*_*j*_ and mean recovery time *w*_*p*_ defined in Eq. (1), which themselves depend in **g**, **f** and **r** in Eq. (2). The product *z* goes over all contact groups and within each contact group: *j* goes over individuals that become infected *excluding* those which initiate epidemics, *p* goes over individuals that become infected *including* those which initiate the epidemics and *e* goes over both infection and recovery events (with corresponding event times *t*_*e*_). Here the notation *j* ∈ *z* indicates that *j* goes over all individuals in contact group *z*, and *e* ∈ *E*_*z*_ indicates that *e* goes over all events *E*_*z*_ in *z*. The force of infection *λ*_*j*_ is calculated immediately prior to individual *j* becoming infected. The gamma distributed probability density function *F*_*Γ*_ for recovery events gives the probability an individual *p* is infected for duration *δt*_*p*_, given a mean *w*_*p*_ and shape parameter *k*. The time dependent total rate of infection events Λ_*z*_ in contact group *z* immediately prior to either an infection or recovery event at time *t*_*e*_ is given by

5 $$\Lambda_{z} (t_{e} ) = \sum\nolimits_{s} {\lambda_{s} } ,$$

where the sum *s* goes over all susceptible individuals in contact group *z* at that time.

An important point to mention is that Eq. (4) is calculated on an unbounded time line. In situations in which data is censored, the observation model restricts events that occur within the observed time window, but other events can exist outside of this observed region.^3^

#### Prior π(θ)

This encapsulates the state of knowledge regarding potential parameter values prior to data *y* being considered (along with capturing correlations between parameters as a result of genetic relatedness). Details of the prior used for this study are given in Additional file 2.

The posterior distribution for parameters *θ* and events *ξ* in Eq. (3) is sampled by means of an adaptive data-augmentation MCMC scheme (see Additional file 3 and [17] for details).

### SIRE 2.0 software tool

The SIRE 2.0 desktop application reads in epidemiological data, user-specified fixed or random effects, and pedigree-based or genomic relationship matrices, and generates posterior estimates using the Bayesian inference methodology outlined above. The software, together with the user guide is freely available to download from https://github.com/theITEAM/SIRE2.0 (with versions for Windows, Linux and Mac). For more detailed information on the software see Additional file 4.

### Analytical expressions for prediction accuracies for the epidemiological host traits

To validate the inference methodology described above, and inform experimental or field study sampling designs [35], we derived analytical expressions for prediction accuracies for the three epidemiological host traits.

Prediction accuracy (PA) *α* is defined as the Pearson correlation coefficient between the estimated additive genetic contribution (the posterior mean) of an individual for a trait and its true value.

Deriving general analytical expressions for PAs for the model above is challenging. Additional file 6 shows, however, that it can be done for a somewhat idealised situation that assumes infection and recovery times of individuals are precisely known (or just infection times in the case of the SI model) as well as ignoring group and fixed effects. Furthermore, the traits are assumed to be uncorrelated with the diagonals of Ω and Ψ having small, known values, and epidemics are instigated by a single randomly infected individual. The basic reproduction number *R*_*0*_ is taken to be sufficiently high such that most individuals become infected.^4^ Since PA estimates vary across individuals (because they depend not only on an individual’s epidemiological data, but also on genetic relatedness to other individuals), it is here defined at an individual level [43].

Under these idealised conditions, the PAs for susceptibility, infectivity and recoverability genetic effects for individual *n* are given by:

6 $$\begin{gathered} \alpha_{g,n} = \frac{{\sqrt {\Omega_{gg} } \sum\nolimits_{s} {{\text{A}}_{ns}^{2} } }}{{\sqrt {\sum\nolimits_{s} {{\text{A}}_{ns}^{2} } + \sum\nolimits_{{z,z^{\prime}}} {\left[ {\sum\nolimits_{{j \in z,j^{\prime} \in z^{\prime}}} {{\text{A}}_{nj} {\text{A}}_{{nj^{\prime}}} \left( {\Omega_{gg} {\text{A}}_{{jj^{\prime}}} + \Psi_{gg} I_{{jj^{\prime}}} } \right.} } \right.} \left. {\left. { + \tfrac{1}{{N^{2} }}\sum\nolimits_{{q \in z,q^{\prime} \in z^{\prime}}} {(\Omega_{ff} {\text{A}}_{{qq^{\prime}}} + \Psi_{ff} {\text{I}}_{{qq^{\prime}}} )} } \right)} \right]} }}{, } \hfill \\ \alpha_{f,n} = \frac{{\sqrt {\Omega_{ff} } \left( {\tfrac{\log (2N)}{N}\sum\nolimits_{m} {{\text{A}}_{nm}^{2} + \sum\nolimits_{z} {\left[ {\tfrac{1}{N}\sum\nolimits_{j \ne q \in z} {{\text{A}}_{nj} {\text{A}}_{nq} } } \right]} } } \right)}}{{\sqrt {\tfrac{\log (2N)}{N}\sum\nolimits_{m} {{\text{A}}_{nm}^{2} } + \sum\nolimits_{{z,z^{\prime}}} {\left[ {\tfrac{1}{{N^{2} }}\sum\nolimits_{{j,q \in z,j^{\prime},q^{\prime} \in z^{\prime}}} {{\text{A}}_{nj} {\text{A}}_{{nj^{\prime}}} \left( {\omega_{{jqj^{\prime}q^{\prime}}} \left( {\Omega_{gg} {\text{A}}_{{qq^{\prime}}} + \Psi_{gg} {\text{I}}_{{qq^{\prime}}} } \right)\left. {\left. {{ + }\eta_{{jqj^{\prime}q^{\prime}}} \left( {\Omega_{ff} {\text{A}}_{{qq^{\prime}}} + \Psi_{ff} {\text{I}}_{{qq^{\prime}}} } \right)} \right)} \right]} \right.} } \right.} } }}, \hfill \\ \alpha_{r,n} = \frac{{\sqrt {\Omega_{rr} } \sum\nolimits_{m} {{\text{A}}_{nm}^{2} } }}{{\sqrt {\tfrac{1}{k}\sum\nolimits_{m} {{\text{A}}_{nm}^{2} + \sum\nolimits_{mq} {{\text{A}}_{nm} {\text{A}}_{nq} \left( {\Omega_{rr} {\text{A}}_{mq} + \Psi_{rr} {\rm I}_{mq} } \right)} } } }}, \hfill \\ \end{gathered}$$

where *s* sums over all initially susceptible individuals^5^ and *m* sums over all individuals that become infected; *z* sums over contact groups and *j* and *q* sum over all individuals within *z*. Similarly, *z′* sums over contact groups and *j*′ and *q*′ sum over all individuals within *z′*. *N* is the number of individuals within each contact group, **Ω** and** Ψ** are, respectively, the covariance matrices for additive genetic and environmental effects, **A** and **I** are, respectively, the relationship and identity matrices and *k* is the shape parameter for recovery. The quantities *ω*_*qjq*′*j*′_ and *η*_*qjq*′*j*′_, which depend on the combination of individuals within and between groups, are as follows:

$$\begin{gathered} \omega_{jqj^{\prime}q^{\prime}} = \left\{ {\begin{array}{*{20}c} {\tau_{jq} \tau_{j^{\prime}q^{\prime}} } & {{\text{if }}j{\text{ and }}q{\text{ different from }}j^{\prime}{\text{ and }}q^{\prime}} \\ 3 & {{\text{if }}j = j^{\prime} \ne q = q^{\prime}} \\ 2 & {{\text{if }}j = j^{\prime} \ne q \ne q^{\prime}{\text{ or }}q = q^{\prime} \ne j \ne j^{\prime}} \\ 0 & {{\text{if }}j = q^{\prime} \ne j^{\prime} = q} \\ \end{array} } \right. \hfill \\ \eta_{jqj^{\prime}q^{\prime}} = \left\{ {\begin{array}{*{20}c} {\nu_{jq} \nu_{j^{\prime}q^{\prime}} } & {{\text{if }}j{\text{ and }}q{\text{ different from }}j^{\prime}{\text{ and }}q^{\prime}} \\ {3.5} & {{\text{if }}j = q = j^{\prime} = q^{\prime}} \\ 2 & \begin{gathered} {\text{if }}j = j^{\prime} \ne q \ne q^{\prime}{\text{ or }}j = q^{\prime} \ne q \ne j^{\prime} \, \hfill \\ {\text{or }}q = j^{\prime} \ne j \ne q^{\prime}{\text{or }}q = q^{\prime} \ne j \ne j^{\prime} \hfill \\ \end{gathered} \\ {\log^{2} (N)} & \begin{gathered} {\text{if }}j \ne j^{\prime} = q = q^{\prime}{\text{ or }}j^{\prime} \ne q = q^{\prime} = j \, \hfill \\ {\text{or }}q \ne j = j^{\prime} = q^{\prime}{\text{or }}q^{\prime} \ne q = j = j^{\prime} \hfill \\ \end{gathered} \\ {2\log (N)} & {{\text{if }}j = j^{\prime} \ne q = q^{\prime}{\text{ or }}j = q^{\prime} \ne q = j^{\prime}} \\ \end{array} } \right. \hfill \\ \tau_{jq} = \left\{ {\begin{array}{*{20}c} 1 & {{\text{if }}j \ne q} \\ 0 & {{\text{if }}j = q} \\ \end{array} } \right., \, \nu_{jq} = \left\{ {\begin{array}{*{20}c} 1 & {{\text{if }}j \ne q} \\ {\log (2N)} & {{\text{if }}j = q} \\ \end{array} } \right. \hfill \\ \end{gathered}$$

Details of this derivation are provided in the Additional file 6. In the results section later the analytical PA expressions in Eq. (6), which were derived using apparently restrictive and simplifying assumptions, are compared with the numerical PAs obtained from applying SIRE 2.0 to data from diverse simulated epidemics. This not only provides validation of the inference methodology and SIRE 2.0, but also assesses the validity of the analytical expressions when some of the underlying assumptions are not met.

#### Interpretation of the analytical PA expressions

Despite the complexity of the expressions in Eq. (6), a number of insights of relevance to future experimental or sampling designs can be obtained:

When genetic and phenotypic variation in traits is small (i.e*.* taking the limit when Ω_gg_, Ω_ff_, Ω_rr_, Ψ_gg_, Ψ_ff_ and Ψ_rr_ all go to zero) the denominators approach constant values and the numerators imply that α_*g,n*_ is proportional to √Ω_*gg*_ (i.e*.* the fractional variation in susceptibility), and similarly α_*f,n*_ is proportional to √Ω_*ff*_ and α_*r,n*_ is proportional to √Ω_*rr*_. The fact that PAs approach zero in this limit makes sense, because if there is only small genetic variation in a trait, it becomes very difficult to order individuals in that trait (e.g. identify which individuals are least to most susceptible). For the latent epidemiological phenotypes this is exacerbated by the fact that traits are not directly measured, and so there is uncertainty in the phenotypes themselves due to noise coming from stochasticity in the epidemics as well as confounding between susceptibility and infectivity effects on observed disease data. Hence, to obtain high PAs for the epidemiological traits reasonably large genetic variances are required.

Since the contributions in the denominators in Eq. (6) are all positive, they all act to reduce PAs. For example, in the denominator for α_*g,n*_ (the susceptibility PA for individual *n*) we see that a large environmental variation in susceptibility Ψ_*gg*_, or large additive genetic and environmental variation in infectivity (Ω_*ff*_, and Ψ_*ff*_), all act to reduce the PA for susceptibility.

It is also worth noting that the values for the mean transmission and recovery rate *β* and *γ* don’t appear in any of the analytic expressions for the PAs in Eq. (6). In fact, they only indirectly influence PAs if their choice leads to a substantial proportion of individuals not becoming infected (which happens if the basic reproductive ratio *R*_*0*_ = *βN*/γ becomes near to or less than 1). This agrees with previous observations that estimation of heritability in disease related traits requires (substantial) disease transmission [16, 44].

Intuitively, the expressions for the PAs for susceptibility *α*_*g,n*_ and infectivity *α*_*f,n*_ in Eq. (6) also indicate that they partly depend on how related individuals are distributed within and across contact groups (as indicated by the terms in the sums over *z*, *z*′ in Eq. (6)). In contrast, the PA for recoverability *α*_*r,n*_ is independent of how individuals are arranged into contact groups.

The sum over all initially susceptible individuals *s* in the numerator of the susceptibility PA expression indicates that information about the corresponding genetic contribution of individual *n* not only comes from its infection time, but also from the infection times of other susceptible individuals, weighted according to their degree of relatedness to *n*. In the case of infectivity and recoverability, dependence of the numerator on the sum over infected individuals *m* reveals that information is obtained via related infected individuals.

However, the contributions to the numerator are not all equal. In particular, the first term in the infectivity PA numerator *α*_*f,n*_ in Eq. (6) is multiplied by a factor log(2*N*)/*N*, indicating PAs for infectivity tend to be lower than those for susceptibility or recoverability, especially when the number of individuals per group *N* is large. This reduction, however, is partly mitigated by the additional second term in the infectivity PA numerator, which sums over pairs of individuals related to *n* within each contact group, indicating that these can provide additional information about individual *n*’s genetic contribution to infectivity. Different denominators in the expressions for the PA in susceptibility and infectivity make it challenging to make a direct comparison of the contributions of related individuals within and across different contact groups to the corresponding PA.

Finally, correlations between epidemiological host traits were assumed to be zero in the derivation of the expressions above, but it should be emphasised that these may also affect PAs (indeed, this is what is observed in the results section later).

### Method validation

To illustrate and validate SIRE 2.0 and the analytical expressions for PAs in Eq. (6), simulated epidemic data were generated using a variety of parameter combinations and population and contact group structures. These data were generated by means of a Doob–Gillespie algorithm [45], modified to account for non-Markovian recovery times (details of this procedure are given in Additional file 7). For each analysis, 20 replicated datasets were generated (by simulating from the model 20 times), to assess the effect of stochastic variation on the results.

#### Simulated scenarios

Two types of scenario were considered for the validation: firstly an “exemplar” scenario that represents a realistic set of model parameters and epidemiological data, and secondly, a simpler set of “baseline” scenarios that allow for the systematic exploration of the role of individual parameters and data structures whilst keeping everything else fixed. Table 2 lists the diverse analyses associated with each scenario. Parameter combinations and epidemiological data for the inferences associated with each scenario are described below.

**Table 2:** Analyses performed

Analysis	Scenario	Disease data for inference	Results
Precision of genetic and epidemiological model parameter estimates	Exemplar	Infection status (i.e*.* classified as S, I or R) at periodic time points	Figure 2
PAs for host susceptibility, infectivity and recoverability	Exemplar	Infection status at periodic time points	Table 3
Effect of genetic and phenotypic covariances on PAs	Baseline	Infection and recovery time^a^	Figure 3
Effect of population structure and contact group size and makeup on PAs^b^	Baseline	Infection and recovery time^a^	Figures 4a-c
Effect of population structure and contact group size and makeup on PAs^b^	Baseline	Recovery time only^a^	Figures 4d-f
Effect of data recording on PAs	Baseline	Time censored infection and recovery times (varying observation period) Infection status at periodic time points (varying sampling frequency)	Figure 5
Effect of varying population structures on PAs	Baseline	Infection and recovery times	Additional file 8
Effect of varying the size of a fixed effect or the group effect ${\sigma }_{c}$ on PAs	Baseline	Infection and recovery times	Additional file 11

This table provides a summary of all analyses carried out to validate SIRE 2.0

^a ^Note, 'recovery' time can, for some diseases, correspond to time of death. Unless otherwise stated, the analyses assumed a population of 2000 individuals, consisting of *P* = 20 paternal half-sib offspring of 100 unrelated sires, each mated to *P* = 20 unrelated dams. Offspring were randomly allocated into *Z* = 200 contact groups of group size *N* = 10

^b ^Specifically, the following parameters were varied: number of sire offspring $P$, contact group size* N*, and number of different paternal half-sib families represented in each contact group. When the number of half-sib families per contact group or the number of sire offspring $P$ were varied, the number of sires was modified accordingly to ensure that the total number of progeny is fixed (as closely as possible) to 2000 individuals

Unless otherwise stated, a population of 2000 individuals was assumed. This consisted of paternal-half sib progeny from 100 unrelated sires, each mated to *P* = 20 dams randomly selected from an unrelated population (resulting in one offspring for each mating). These 2000 progeny were randomly allocated into *Z* = 200 contact groups of size *N* = 10. The effect of alternative population structures or allocation of individuals on PAs was also investigated, as outlined in Table 2 and Additional file 8. In cases in which *P* or *N* were altered, the number of sires and/or contact groups were adjusted, as closely as possible, to retain 2000 progeny.

#### Exemplar scenario

To represent a “typical” possible scenario, the following choices for the epidemiological parameters were made: *β* = 0.05 and *γ* = 0.1 such that the basic reproductive ratio (assuming no variation in traits) is given by *R*_*0*_ = *βN*/γ = 5, and *k* = 3 such that the infection duration distribution has a wide but peaked distribution around its mean.^6^. The genetic **Ω** and environmental **Ψ** covariance matrices for this set were taken to be:

7 $${{\varvec{\Omega}}} = \left[ {\begin{array}{*{20}c} {0.33} & {0.30} & { - 0.044} \\ {0.30} & {1.68} & { - 0.5} \\ { - 0.044} & { - 0.5} & {0.6} \\ \end{array} } \right], \, {{\varvec{\Psi}}} = \left[ {\begin{array}{*{20}c} {0.77} & {0.56} & { - 0.25} \\ {0.56} & {1.12} & { - 0.4} \\ { - 0.25} & { - 0.4} & {0.9} \\ \end{array} } \right],$$

giving a total phenotypic covariance matrix **Θ** = **Ω** + **Ψ** of

8 $${{\varvec{\Theta}}} = \left[ {\begin{array}{*{20}c} {1.0} & {0.86} & { - 0.29} \\ {0.86} & {2.8} & { - 0.9} \\ { - 0.29} & { - 0.9} & {1.5} \\ \end{array} } \right].$$

Although the choices for these matrices were essentially arbitrary, we outline some of the guiding principles which have been used:

Equation (8) implies a large phenotypic variation in infectivity Θ_*ff*_ = 2.8, which is consistent with the often quoted “80/20 rule” characteristic of super-spreaders, in which ≈ 20% of infected individuals cause ≈ 80% of infections (see Additional file 9 for details [46]).

Given that evolutionary theory suggests that selection acts against high susceptibility and possibly also low recoverability (e.g. [47]), one would expect genetic variation in these traits to be lower compared to infectivity. Consequently we have chosen the variance in the genetic contribution to infectivity in Eq. (7) to be a relatively large value of ${\Omega }_{ff}$ = 1.68 compared to the smaller values of ${\Omega }_{gg}$=0.33 for susceptibility and ${\Omega }_{rr}$ = 0.6 for recoverability.

Heritabilities were chosen to lie in a reasonable range consistent with other polygenic traits: ${h}_{g}^{2}={\Omega }_{gg}/{\Theta }_{gg}=0.33$ for susceptibility, ${h}_{f}^{2}={\Omega }_{ff}/{\Theta }_{ff}=0.6$ for infectivity and ${h}_{r}^{2}={\Omega }_{rr}/{\Theta }_{rr}=0.4$ for recoverability. Note that moderately high heritability values for underlying latent epidemiological phenotypes result in substantially lower heritability estimates for observed individual traits [9], such as infection or recovery times, because the latter combines uncertainty in the traits themselves with inherent stochasticity in the spread of disease.

Finally, trait covariances were chosen based on the expectation that highly susceptible individuals have a weaker immune response to inhibit pathogen replication, and are thus likely to be infectious for longer; consequently we assumed a positive correlation between these two traits: Ω_*gf*_/√(Ω_*gg*_Ω_*ff*_) = 0.4 for the genetic and Ψ_*gf*_/√(Ψ_*gg*_Ψ_*ff*_) = 0.6 for the environmental contributions. Similarly, more susceptible or more infectious individuals might be expected to recover more slowly, hence resulting in negative correlations: Ω_*gr*_/√(Ω_*gg*_Ω_*rr*_) = − 0.1 and Ω_*fr*_/√(Ω_*ff*_Ω_*rr*_) = − 0.4 for the genetic, and Ψ_*gr*_/√(Ψ_*gg*_Ψ_*rr*_) = − 0.3 and Ψ_*fr*_/√(Ψ_*ff*_Ψ_*rr*_) = − 0.4 for the environmental contributions.

For simplicity no fixed effects were incorporated into the model, and the standard deviation in the random contact group effects was taken to be σ_c_ = 0.25 (corresponding to an approximate 25% variation in transmission rate across different contact groups).

For the exemplar scenario it was assumed that infection and recovery times are unknown. Instead, the disease status of individuals is periodically checked every Δ*T* = 2 time units up until *T*_max_ = 40 (hence 20 measurements in all are made on each individual, along with their initial disease status).

#### Baseline scenario

The exemplar parameter set above represents a plausible realistic scenario, but used many essentially arbitrary parameter choices which are not appropriate when conducting a systematic exploration of the parameter domain. To this end we chose a simplified baseline set of model parameters associated with the three host epidemiological traits:

9 $${{\varvec{\Omega}}} = \left[ {\begin{array}{*{20}c} {h_{g}^{2} \Theta_{gg} } & 0 & 0 \\ 0 & {h_{f}^{2} \Theta_{ff} } & 0 \\ 0 & 0 & {h_{r}^{2} \Theta_{rr} } \\ \end{array} } \right],\quad \quad {{\varvec{\Psi}}} = \left[ {\begin{array}{*{20}c} {(1 - h_{g}^{2} )\Theta_{gg} } & 0 & 0 \\ 0 & {(1 - h_{f}^{2} )\Theta_{ff} } & 0 \\ 0 & 0 & {(1 - h_{r}^{2} )\Theta_{rr} } \\ \end{array} } \right],$$

which, by default, doesn’t include correlations. Unless stated, heritabilities were assumed to be equal with ${h}_{g}^{2}={h}_{f}^{2}={h}_{r}^{2}=0.5$. With these choices, the phenotypic variance is given by the diagonal identity matrix:

10 $${{\varvec{\Theta}}} = \left[ {\begin{array}{*{20}c} 1 & 0 & 0 \\ 0 & 1 & 0 \\ 0 & 0 & 1 \\ \end{array} } \right].$$

Values for the epidemiological model parameters *β*, *γ* and *k*, were the same as for the exemplar scenario. However, for the baseline scenarios, individuals’ infection and recovery times were mostly assumed to be known, except for when the impact of different data structures on the parameter estimates was explicitly investigated (Table 2). For the baseline scenario with known infection and recovery times, PAs obtained from the simulated data were compared to the corresponding analytical predictions derived in Eq. (6).

Note, for the default population structure, consisting of *P* paternal half-sibs randomly allocated into contact groups of size *N*, the analytic expressions for the PAs in Eq. (6) for the sire subpopulation^7^ simplify to (see Additional file 10 for details):

11 $$\begin{gathered} \alpha_{g,SIRE} = \left[ {1 + \tfrac{4N}{{P\Omega_{gg} (N - 1)}}\left( {1 + \Theta_{gg} + \tfrac{1}{N}\Theta_{ff} } \right)} \right]^{{ - \tfrac{1}{2}}} , \hfill \\ \alpha_{f,SIRE} = \left[ {1 + \tfrac{4N}{{P\Omega_{ff} \log (2N)}}\left( {1 + 2\Theta_{ff} + \tfrac{3}{\log (2N)}\Theta_{gg} } \right)} \right]^{{ - \tfrac{1}{2}}} , \hfill \\ \alpha_{r,SIRE} = \left[ {1 + \tfrac{4}{{P\Omega_{rr} }}\left( {\tfrac{1}{k} + \Theta_{rr} } \right)} \right]^{{ - \tfrac{1}{2}}} . \hfill \\ \end{gathered}$$

This shows that, even if susceptibility and infectivity are uncorrelated, PA for susceptibility depends not only on variation in susceptibility, but also on phenotypic variation in infectivity, and vice versa. Furthermore, Eq. (11) demonstrates that PAs for infectivity are generally lower than those for recoverability and susceptibility if all three host epidemiological traits have equal genetic and phenotypic variances (and *k* > 1, which would be expected). Specifically, for the parameter values stated above, sire prediction accuracies from Eq. (11) are 0.81 and 0.72 for recoverability and susceptibility, respectively, and only 0.24 for infectivity. Interestingly, these expressions do not contain the number of contact groups *Z*, which means that even as *Z* becomes large, the PAs are not expected to approach 1 (remember even in this limit a given sire only has information from its *P* progeny, which is fixed, and that information is diluted by the stochasticity of the epidemic process). This is in contrast to estimates for other model parameters, such as genetic variances, which do become more and more exact in the limit of large *Z*.^8^

## Results

### Analysis of the exemplar scenario

**Fig. 1:**
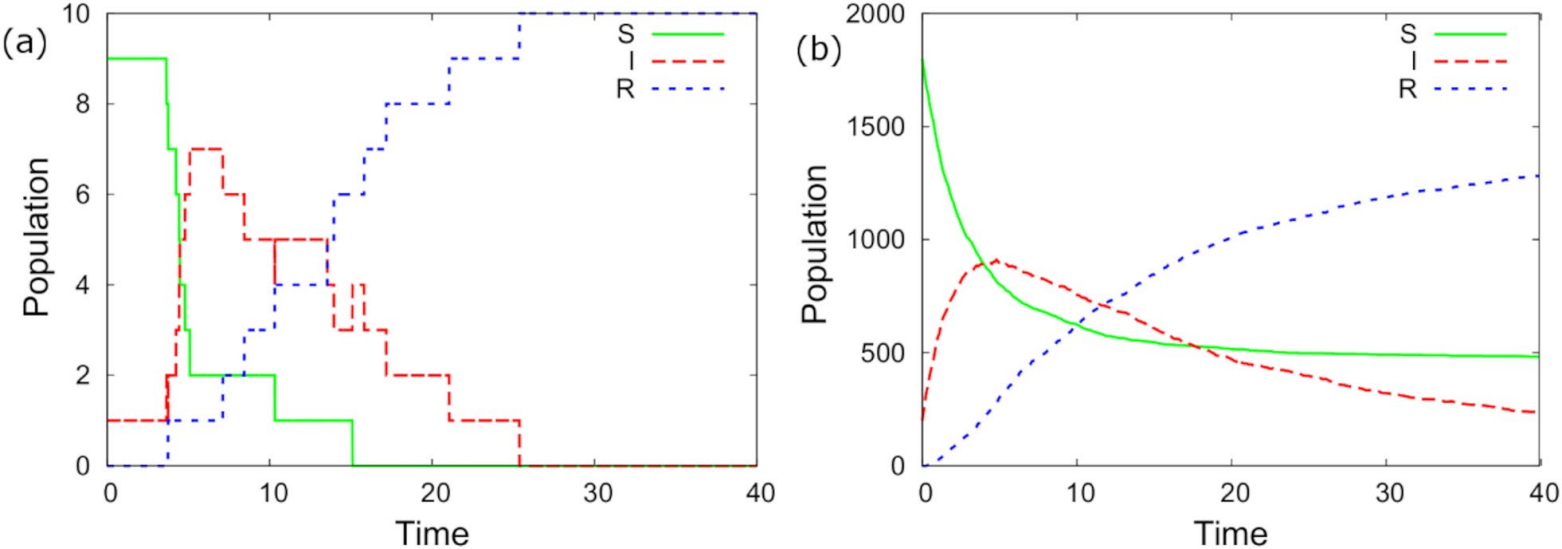
Population dynamics corresponding to the exemplar parameter set. **a** Simulated dynamic changes within the susceptible (S), infected (I) and recovered (R) populations within a single contact group consisting of *N* = 10 individuals, one of which is initially infected. **b** Collective dynamics summing over simulated epidemics from *Z* = 200 separate contact groups

Figure 1a shows the simulated population dynamics of a single contact group containing *N* = 10 individuals for the exemplar parameter set. The total number of infected individuals, as shown by the red curve, starts at one and rapidly increases until all individuals become infected^9^ and then recover. The discontinuous changes to the curves arise because of discrete infection and recovery events. Figure 1b shows the collective dynamics over the *Z* = 200 contact groups, resulting in smooth S-I-R profiles.

**Fig. 2:**
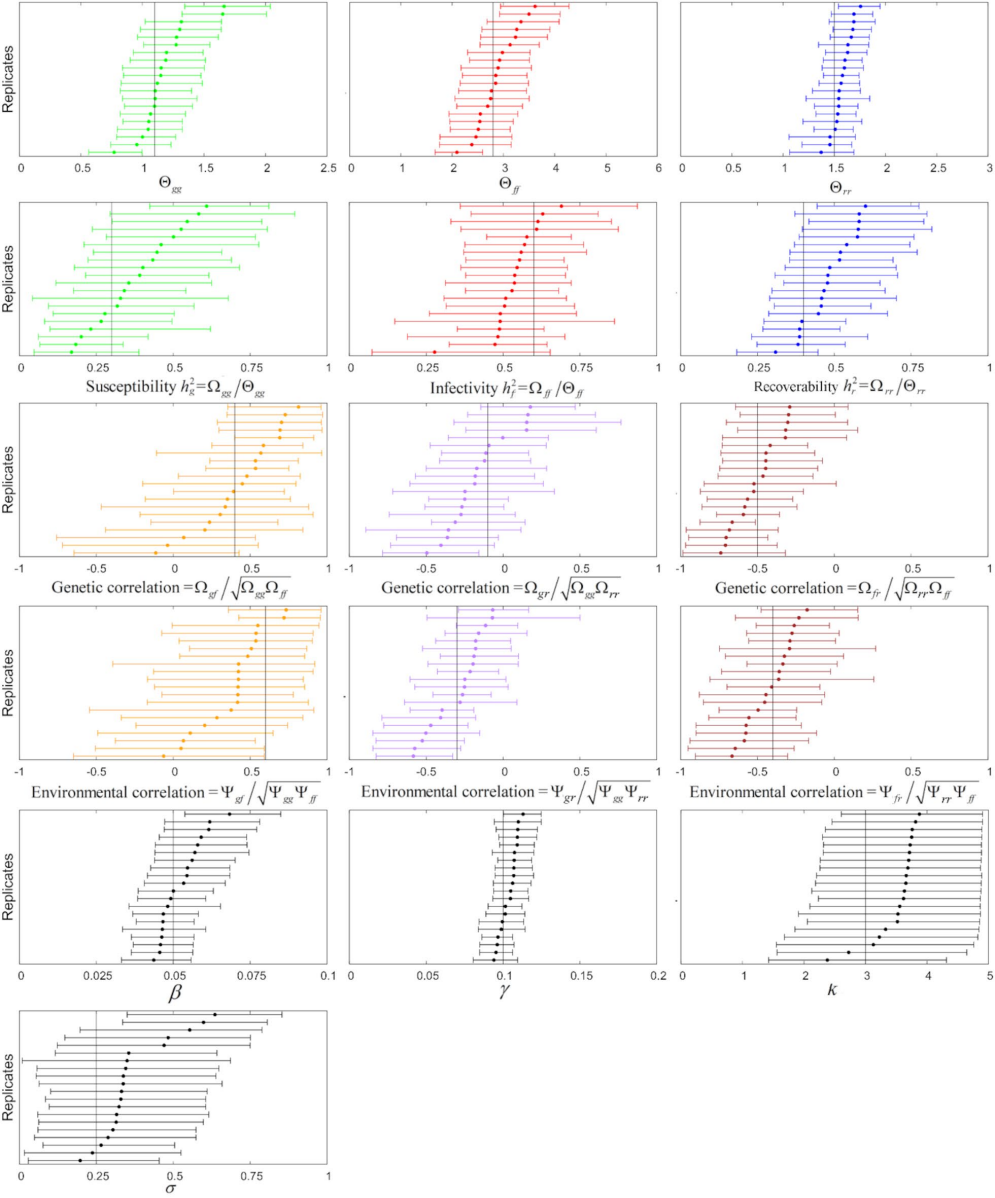
Tornado plots for posterior distributions for genetic and epidemiological model parameters. These show the posterior means (circles) and 90% credible intervals for different model parameters (see description in Table 1) inferred from 20 replicated datasets (generated from the exemplar scenario). Note, in each plot the distributions are ordered by posterior mean, leading to the characteristic tornado shape. The vertical black lines represent the true parameter values. Priors are defined in Additional file 2

Figure 2 shows tornado plots of posterior distributions for each model parameter generated by SIRE 2.0, using twenty simulated data replicates.^10^ For all model parameters, the true values lie within the 90% credible intervals obtained from Bayesian inference for at least 17 out of the 20 replicates, and in close proximity to the credible intervals in all replicates. This indicates that the inference procedure is able to provide calibrated interval estimates for the entire set of epidemiological and genetic model parameters.

As indicated by the width and symmetry of the credible intervals about the true value, the precision with which different parameters can be estimated varies. Relatively precise and unbiased estimates were obtained for the population-wide transmission rate *β* and recovery rate *γ* as well as the phenotypic variances for the susceptibility Θ_*gg*_, infectivity Θ_*ff*_, and recoverability Θ_*rr*_. Slightly less precise estimates and a tendency for slight bias were obtained for the narrow-sense heritabilities ${h}_{g}^{2}, {h}_{f}^{2}, {h}_{r}^{2}$ and the genetic and environmental correlations, as indicated by the larger uncertainty and slightly skewed intervals. In particular, the heritability posterior means for susceptibility ${h}_{g}^{2}$ tended to slightly over-estimate the true value, whereas those for infectivity ${h}_{f}^{2}$ tended to more often under-estimate it. However, the lower boundaries of the credible intervals for heritability estimates were substantially larger than zero for all replicates, showing that SIRE 2.0 can capture heritable genetic variation in all three traits, if it exists and is moderately high. Similarly, the majority of replicates produced posterior means and credible intervals for the genetic and environmental correlations that correctly indicate whether the epidemiological traits were positively or negatively correlated.

Finally, relatively wide distributions were obtained for the shape parameter *k* and contact group effect size σ_c_, although the posterior distributions clearly preclude the value *k* = 1 (meaning that inference can determine that the infectious duration distribution is unimodal, but is uncertain as to how sharp that mode is) and σ_c_ = 0 (meaning that group effects can be unambiguously detected).

Table 3 shows PAs for the exemplar scenario. It is noteworthy that PAs are generally higher for sires (varying between 0.57 and 0.74 for different traits) than for their offspring (between 0.30 and 0.56), despite the fact that no direct epidemiological data is obtained for these individuals. This emphasises that knowledge of an individual’s traits is very much linked to data from closely related individuals, as was demonstrated in Eq. (6). Since the PAs of sires are of critical importance in many breeding programs, these will be the focus for the remainder of the paper.

**Table 3:** Prediction accuracies

	Susceptibility PA	Infectivity PA	Recoverability PA
Sires	α_*g,SIRE*_ = 0.57	α_*f,SIRE*_ = 0.62	α_*r,SIRE*_ = 0.74
Progeny	α_*g,PRO*_ = 0.30	α_*f,PRO*_ = 0.40	α_*r,PRO*_ = 0.56

This table shows prediction accuracies (PAs) for the different host traits for sires (top row) and offspring (bottom row) under the exemplar scenario (see “Method validation” section for a description of this)

Generally, the highest PAs in Table 3 are for recoverability, but it is noteworthy that PAs for infectivity were higher than those for susceptibility for the exemplar scenario (Eq. (7)). This is for two reasons: Firstly, relatively strong genetic correlation between infectivity and recoverability (− 0.4) compared to that between susceptibility and recoverability (− 0.1) mean that good estimates for recoverability yield improved estimates for infectivity, and secondly, genetic variation in infectivity ${\Omega }_{ff}=1.68$ was assumed to be substantially higher than that for susceptibility ${\Omega }_{gg}=$ 0.33.

### Prediction accuracies (PAs) using the baseline scenario

The following sections utilise simulations for the baseline scenario from above and explore how PAs vary as a result of changes made to different aspects of the model:

#### PA dependence on genetic and environmental variances and correlations

**Fig. 3:**
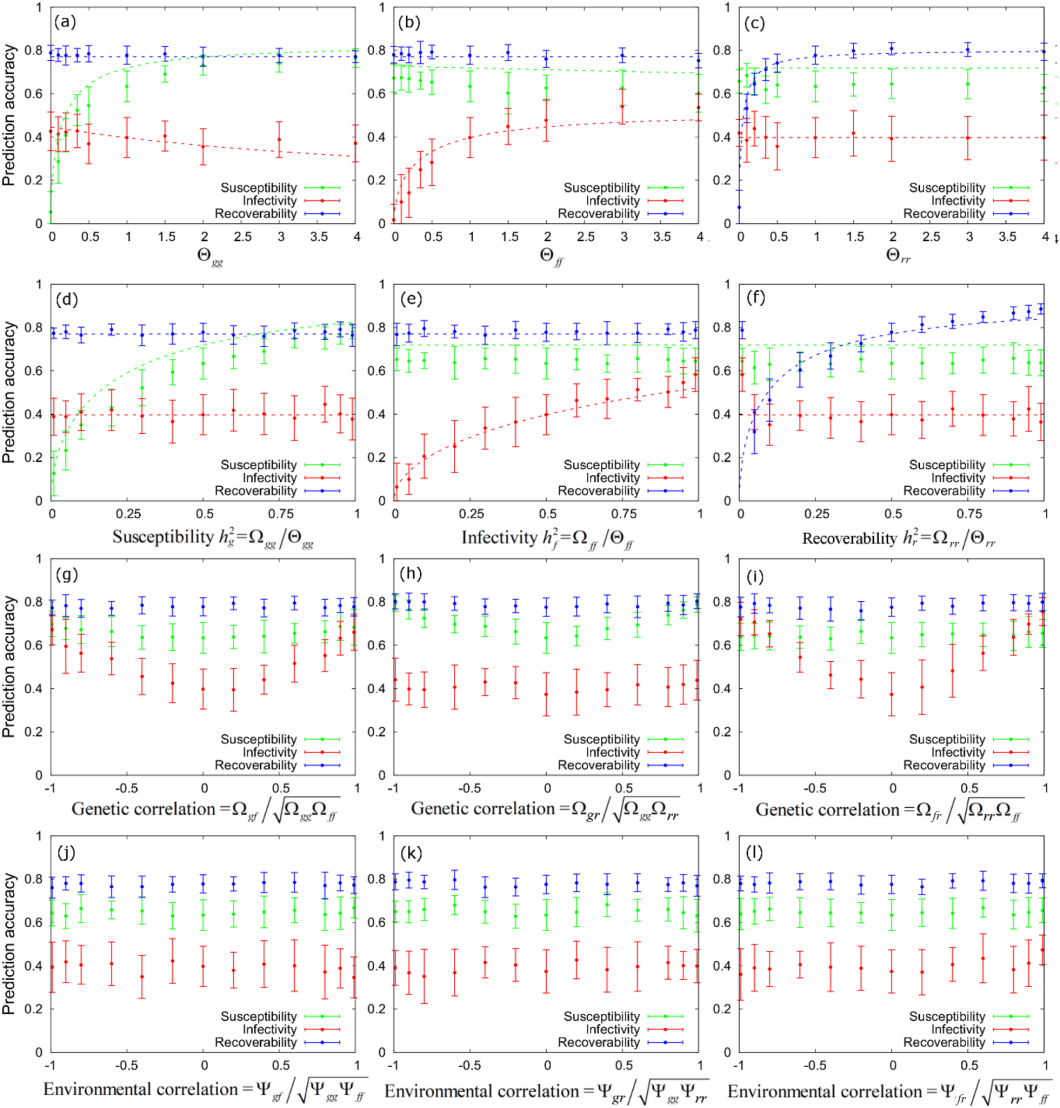
Dependency of PAs on genetic and environmental covariance matrices. These plots show how sire PAs for susceptibility (green), infectivity (red) and recoverability (blue) vary with phenotypic variances (**a**–**c**), heritabilities (**d**–**f**), genetic (**g**–**i**) and environmental (**j**–**l**) correlations derived from the additive genetic **Ω**, environmental **Ψ** and phenotypic **Θ** = **Ω** + **Ψ** covariance matrices. Results were generated from simulated data using the baseline scenario with known infection and recovery times (see Table 2). The circles with error bars give the mean and standard deviation of numerical estimates obtained from 20 simulated datasets. The analytical dashed lines come from Eq. (6) (note, they are not applicable to (**g**–**l**) because analysis assumed uncorrelated traits)

Figure 3 shows how PAs for sire susceptibility, infectivity and recoverability change with respect to changes in genetic and environmental variances and covariances for these traits, assuming that infection and recovery times are known. SIRE 2.0 results are provided by the symbols (where the error bars indicate variation over 20 replicates) and the corresponding analytical expressions from Eq. (11) are shown by the dashed lines. Below we discuss the results for the different types of variation being investigated:

#### Phenotypic variation

Assuming fixed heritabilities, PAs for each epidemiological trait generally increase with increasing phenotypic (and consequently increasing genetic) variance in that trait until they reach an asymptote, as shown in Figs. 3a–c and indicated in Eq. (11). In fact, PA for α_*g,SIRE*_ in Fig. 3a asymptotically approaches^11^$$\left[ {1 + \left( {4/\left[ {Ph_{g}^{2} } \right]} \right)\left( {N/\left[ {N - 1} \right]} \right)} \right]^{{ - \raise.5ex\hbox{$\scriptstyle 1$}\kern-.1em/ \kern-.15em\lower.25ex\hbox{$\scriptstyle 2$} }}$$. The corresponding asymptotes for α_*f,SIRE*_ and α_*r,SIRE*_ in Figs. 3b, c are $$\left[ {1 + 8N/\left( {Ph_{f}^{2} \log \left( N \right)} \right)} \right]^{{ - \raise.5ex\hbox{$\scriptstyle 1$}\kern-.1em/ \kern-.15em\lower.25ex\hbox{$\scriptstyle 2$} }}$$ and $$\left[ {1 + 4/\left( {Ph_{r}^{2} } \right)} \right]^{{ - \raise.5ex\hbox{$\scriptstyle 1$}\kern-.1em/ \kern-.15em\lower.25ex\hbox{$\scriptstyle 2$} }}$$, respectively. The observed slight decrease of α_*f,SIRE*_ with increasing Θ_*gg*_ in Fig. 3a, and similarly a slight decrease of α_*g,SIRE*_ with increasing phenotypic variance in infectivity Θ_*ff*_ in Fig. 3b, is caused by the fact that PA for susceptibility depends on the phenotypic variance in both susceptibility and infectivity, and vice versa for infectivity (see Eq. (11)).

#### Heritability

Figure 3d shows how PAs vary with increasing susceptibility heritability ${h}_{g}^{2}={\Omega }_{gg}/{\Theta }_{gg}$ (which was achieved by increasing the genetic variance ${\Omega }_{gg}$ and decreasing the environmental variance ${\Psi }_{gg}$ such that the phenotypic variance ${\Theta }_{gg}$ remains constant, as shown in Eq. (9)). We find that this increases the PA for susceptibility, whilst leaving the PAs for the remaining two traits approximately the same. This is in accordance with Eq. (11), because α_*g,SIRE*_ depends on ${\Omega }_{gg}$ and α_*f,SIRE*_ and α_*r,SIRE*_ are related only to quantities being fixed. A similar pattern is seen for variation in the heritability for infectivity and recoverability, as shown in Figs. 3e, f. Similar to what was observed for changes in variances above, the biggest rate of change in PAs occur for low heritability values.

#### Genetic trait correlations

Figures 3g–i show the effect of genetic correlations between the host epidemiological traits on PAs (note, analytic estimates for PAs are not shown here because they were only derived for the uncorrelated case). Whereas PA for susceptibility are not much affected by genetic correlations between susceptibility and infectivity, the PA for infectivity *α*_*f,SIRE*_ approaches that of *α*_*g,SIRE*_ as the traits become increasingly correlated (Fig. 3g). This effect, coupled with a larger genetic variance in infectivity, can lead to the PA for infectivity actually being higher than that for susceptibility, as was demonstrated for the exemplar scenario in Table 3. As expected, PAs for recoverability are not affected by genetic correlations between susceptibility and infectivity. In fact, Figs. 3g–i shows similar patterns for all pairwise genetic correlations: when two traits become increasingly genetically correlated, the PA for the trait with the lower PA approaches that of the correlated trait. Taken together this means that the analytical expression in Eq. (6) and Eq. (11) actually represent lower bounds based on uncorrelated traits, as any genetic correlations are found to increase PAs.

#### Environmental trait correlations

In addition to genetic correlations in traits, it is possible to imagine that environmental correlations may also exist. For example, adverse weather conditions might lead to higher infectiousness and longer recovery times. Figures 3j–l show that PAs for genetic effects are, to a large extent, *independent* of environmental correlations, as would be expected.

#### PA dependence on population and contact group structure

To investigate the effect of population structure and genetic relatedness within and across contact groups, the impact of the number of offspring per sire and their allocation across contact groups, and the size of the contact groups on the analytical and numerical PAs were looked at (Table 2).

#### Number of offspring P

**Fig. 4:**
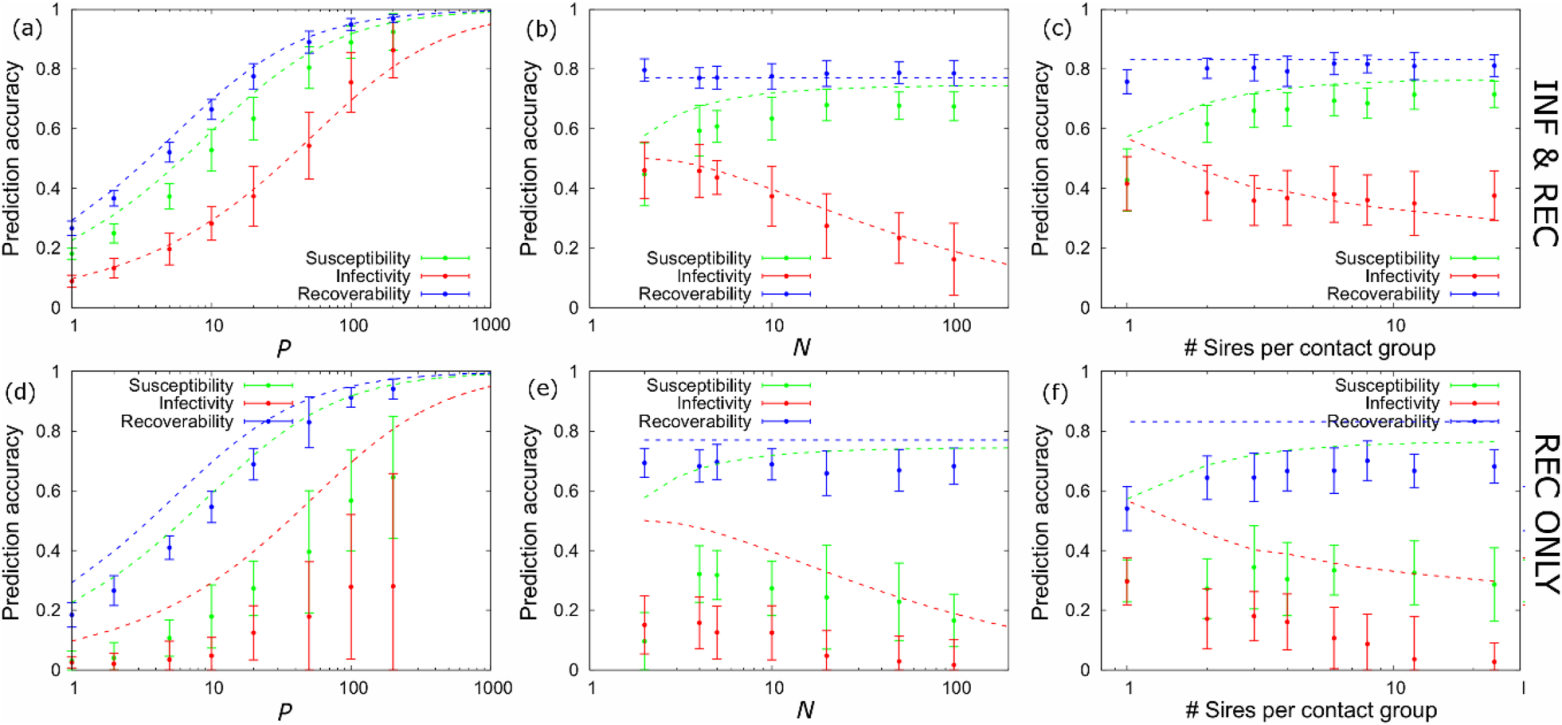
Dependency of PAs on population and contact group structure. These plots show how sire PAs for the susceptibility (green), infectivity (red) and recoverability (blue) vary with: **a**, **d** the number of offspring per sire *P*, **b**, **e** the number of individuals per contact group *N*, and **c**, **f** the number of paternal half-sib families allocated to each contact group. In graphs (**a**–**c**) it was assumed that both infection and recovery times are known; graphs (**d**–**f**) are corresponding graphs when only recovery times are known. Data were generated using the baseline scenario (outlined in “Method validation” section). For graphs (**c**, **f**) it was assumed that each contact group contains offspring from a certain number of sires (as shown on the *x*-axis). The contact group size was set to *N* = 24 and each sire had *P* = 24 offspring that are divided amongst contact groups in various ways (the number of sires and contact groups *Z* were both fixed to 84 giving 2016 progeny). Hence, on the left-hand side of the graphs (1 sire per contact group) all 24 offspring were placed into a single contact group. For the next point along they are split into two subgroups of 12 paternal offspring placed into two different (randomly chosen) contact groups. Subgroups are made smaller and smaller until finally, on the right-hand side of the graph, offspring are individually placed across 24 groups. In all graphs, the circles with error bars give the mean and standard deviation of numerical estimates obtained from 20 simulated datasets. The analytical dashed lines also refer to the baseline parameter set and come from Eq. (6)

Figure 4a shows that PAs for all three epidemiological host traits increase substantially as the number of a sire’s offspring *P* increases (with the number of sires adjusted to keep the progeny population size approximately fixed). For example, sire PAs for all three traits are more than three times higher for sires with *P* = 100 half-sib off-spring compared to *P* = 2. In particular, for infectivity, data from more than *P* = 50 paternal offspring are required to achieve sire PAs above 0.5.

#### Size of contact groups N

Figure 4b shows that the PA for susceptibility *α*_*g,SIRE*_ increases slightly^12^ tailing off to an asymptotic limit as contact group sizes become large (here the number of contact groups were adjusted to keep the progeny population size approximately fixed). In contrast, the PA for infectivity *α*_*f,SIRE*_ decreases because larger contact group sizes mean that information regarding who acquires infection from whom becomes less certain. This is also apparent in the expression for ${\alpha }_{f,SIRE}$ in Eq. (11) because of the fraction *N*/log(2*N*) which becomes larger as *N* increases. PAs for recoverability *α*_*r,SIRE*_ are independent of the size of contact groups (Fig. 4b and Eq. (11)).

#### Allocation of offspring

The scenarios considered so far assume that related individuals (here paternal half-sib offspring) are randomly allocated across different contact groups. Figure 4c shows how sire PAs change as the number of half-sib families per contact group increases, based on simulated data and the corresponding analytical expressions (see Additional file 10, Eq. (A4)). Taken together, these results show that the PA for infectivity *α*_*f,SIRE*_ increases slightly, whereas the PA for susceptibility *α*_*g,SIRE*_ tends to decrease as individuals in contact groups become more highly related (i.e. as we move from right to left in Fig. 4c). The trends are, however, more pronounced for the analytical approximations than for the simulations. In particular, when contact groups contain only offspring from one or two sires the analytical approximations result in slightly higher PAs than those obtained from the simulations. Both analytical and simulated results show, however, that PAs become identical when all progeny for each contact group originate from the same sire. This is because susceptibility and infectivity become entirely confounded in this case.

#### Inclusion of fixed effects or random group effects

Additional file 11 shows that PAs are largely independent of the presence or magnitude of fixed or random group effects in the model (justifying their omission from the analytical derivations in Additional file 6).

#### Extension to other population structures

Additional file 8 investigates scenarios other than the simple paternal half-sib mating structure used in the validation models. Specifically, full-sib families are considered, as well as adding relationships between parents (generated though mating over multiple generations). Relatively weak dependency between PAs and population structure is found, in good agreement with the analytical predictions from Eq. (6).

#### Alternative data recording scenarios

Results related to the baseline scenarios discussed so far have assumed that exact infection and recovery times are known over the entire course of the epidemics. In most real-world applications, however, such precise data is typically unavailable. Here we consider how PAs change under three different data recording scenarios:

#### Recovery times only

This scenario assumes that infection times are unknown and only individuals’ “recovery” times, which may represent their time of death, are the only disease data available. Results corresponding to this scenario are shown in Fig. 4d–f, where the analytic results are shown for comparison as these assume full knowledge of infection and recovery times. Whilst the PA for recoverability is only marginally reduced, PAs for susceptibility and infectivity drop substantially compared to when infection times are also known (Fig. 4a–c). This strong reduction in PA is partly mitigated if the number of related animals is large (e.g*.* a large number of offspring *P* per sire*,* as shown in Fig. 4d, which would need to exceed around 100), contact groups are relatively small (Fig. 4e) and (for estimating infectivity) contain mainly (though not exclusively) related individuals (Fig. 4f).

#### Time censoring

**Fig. 5:**
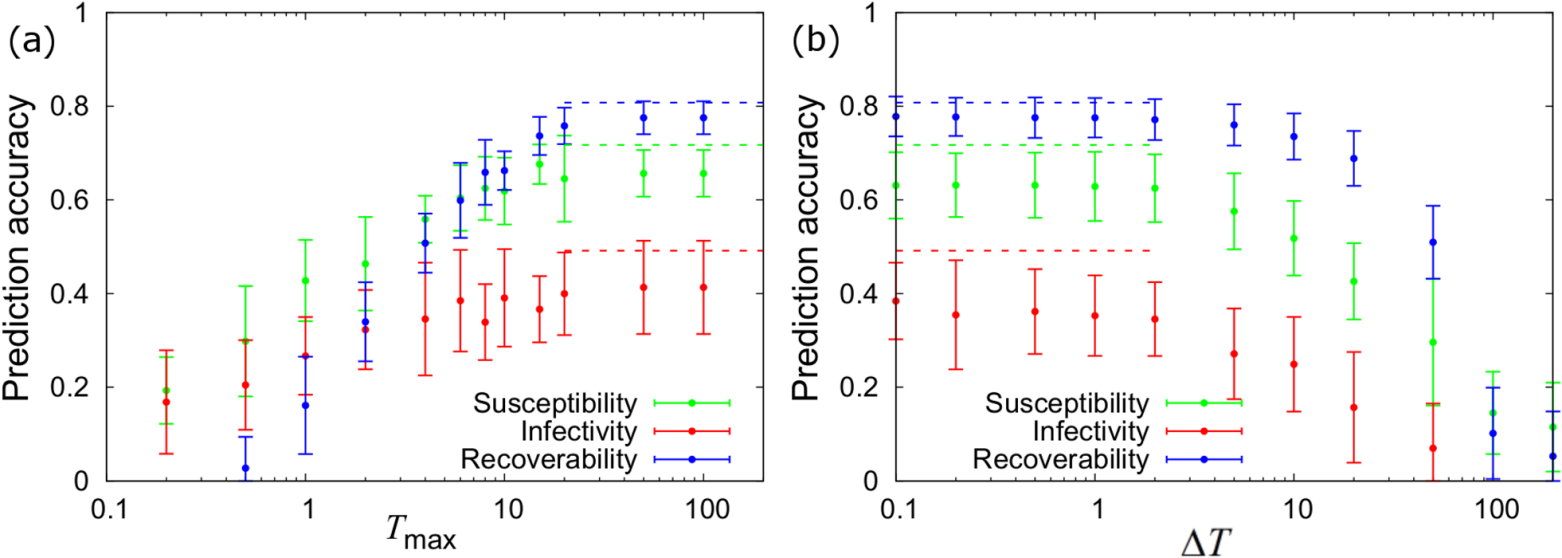
Time censoring and periodic measurements. These plots show how sire PAs for susceptibility (green), infectivity (red) and recoverability (blue) vary with: (**a**) the total time over which events are observed T_max and (**b**) the time interval between periodic infection status statusmeasurements Δ*T*. For reference the mean infection time for individuals is 5.2 and the mean recovery time is 22.1. Data were generated using the baseline scenario (Table 2). The circles with error bars give the mean and standard deviation of numerical estimates obtained from 20 simulated datasets. The analytical dashed lines also refer to the baseline parameter set and come from Eq. (6) (note, they assume *T*_max_ = ∞ and Δ*T* = 0)

Figure 5a shows PAs for all three epidemiological host traits when measurements of individuals’ infection and recovery time are only recorded up to a final time* T*_max_ (with information about any subsequent dynamics missing). The large *T*_max_ limit corresponds to the scenario in which epidemics are fully observed (the dashed lines show the analytical expressions from Eq. (6)). Only when *T*_max_ falls below around 20 do we see some noticeable reduction in PAs (note, the mean recovery time for individuals for this particular dataset was 22.1, so the value 20 corresponds to censoring more than half the recovered individuals). This decline is most pertinent in the PA for recoverability, because recovery events happen later on, so they will tend to become censored first. However, even when *T*_max_ is relatively small (say *T*_max_ = 2), Fig. 5a shows that useful information can still be obtained for all three host epidemiological traits.

#### Periodic measurements

Rather than knowing infection and recovery times, Fig. 5b considers periodic disease status measurements made at intervals of Δ*T* on each individual (as was assumed for the exemplar scenario). For very frequent measurements (i.e*.* in the small Δ*T* limit) the results are essentially the same as knowing event times exactly. As ΔT increases we find that PAs start reducing (beyond around Δ*T* ≈ 3). Even when Δ*T* reaches 20 (which covers a substantial period of the entire epidemic, as seen in Fig. 1b), PAs are still found to be reasonably high. This means that relatively infrequent disease checks may be sufficient to obtain informative estimates of individual’s genetic susceptibility, infectivity and recoverability.

## Discussion

### Novelty and significance

The inference methodology and software tool SIRE 2.0 introduced in this paper constitute an important milestone in the genetic analyses of infectious disease data. The important role of host genetics on disease transmission is well recognized [16, 44, 48, 49]. However very few studies consider individuals’ genetic contributions to transmitting infections to others (infectivity) [12, 21, 34, 50, 51]. Almost all genetic studies of disease data to date exclusively focus on either the genetic risk of becoming infected or developing disease (susceptibility), or dying from infection (recoverability/tolerance). There is currently no available method able to exploit available data to estimate individuals’ genetic contributions to all three of these disease phenotypes, in the likely case they are under polygenic control.

The methodology and software presented here allow simultaneous estimation of variance components and additive genetic contributions for host susceptibility, infectivity and recoverability from a range of epidemiological data that can be readily collected in experimental or field studies. Provided that data from epidemics in multiple contact groups are available, reliable genetic parameter estimates and good prediction accuracies for all three host traits can be obtained from disease records and sample sizes analogous to those currently used for quantitative genetic analyses for disease resistance or resilience [52]. Furthermore, current frequentist approaches typically need precise information of individuals’ infection status at frequent time intervals [29, 30], which are rarely available in practice. In contrast SIRE 2.0 is considerably more flexible, being able to cope with a wide range of incomplete and uncertain assessment of individuals’ disease status.

The ability to estimate genetic parameters for host infectivity, in addition to susceptibility and recovery, opens a greater range of opportunities for more effective infectious disease control. In particular, evolutionary theory predicts relatively large genetic variance in infectivity compared to susceptibility or recoverability [53], and this is backed up by recent empirical findings [50]. Modelling studies suggest this largely untapped source of genetic variation could be exploited to more effectively reduce disease transmission in livestock through genetic selection, mitigating also the risk of pathogen evolution towards increased virulence [12, 22]. Super-spreaders, characterized as a small proportion of highly infectious individuals, have been linked to large outbreak size [54–57]. SIRE 2.0 can account for skewed distributions in host infectivity associated with super-spreaders,^13^ quantify to what extent super-spreading is genetically controlled, and, should they exist, enable identification of individuals with high genetic risk of becoming a potential super-spreaders. Early identification and removal has been suggested as an effective means to prevent large-scale epidemics or pandemics [54].

SIRE 2.0 also provides reliable estimates for genetic correlations between traits, which is a significant advance over previously published methods to infer variance components associated with epidemiological host traits (e.g. [19, 21, 33, 53]). Such estimates are crucial for understanding the evolutionary dynamics of infectious diseases and potential epidemiological trade-offs. From a biological viewpoint, genetic correlations between susceptibility, infectivity and recoverability (encompassing infection-induced mortality) may be expected, as all three traits are likely to be associated with within-host pathogen load, which is partly controlled by host genetics (e.g. [58–60]). For example, genetically more susceptible individuals may have lower capacity to reduce pathogen replication, and thus have higher within-host pathogen load, which likely affects pathogen excretion and hence their infectivity, as well as propensity to recover (e.g. [61]).

Simulation studies have demonstrated that genetic selection for low susceptibility or mortality may not effectively reduce disease prevalence, if antagonistically genetically related to infectivity [20, 22, 23]. In aquaculture and various livestock species, breeding for disease resistance is currently based on mortality data as the disease phenotype [62, 63]. However, this could unintentionally result in production of genetically tolerant super-spreaders if unfavourable correlation with infectivity is not taken into account [64].

### Summary and discussion of main results associated with prediction accuracies (PAs)

As with any new method, systematic investigation of SIRE 2.0’s computational predictions using a variety of simulated data scenarios is essential for validating the methodology and understanding its limits. Validation was further facilitated by derivation of analytic expressions for prediction accuracies (PAs) for the three host traits assuming simplified conditions. These showed good agreement with the equivalent numerical predictions obtained by SIRE 2.0 for scenarios where these assumptions are met. This shows that SIRE 2.0 is able to predict additive genetic contributions for host susceptibility, infectivity and recoverability with the expected accuracy under ideal data scenarios (i.e. balanced population and groups structure and known infection and recovery times). Furthermore, deviations between the numerical and analytical predictions under less ideal data scenarios provide valuable insights into the importance of the assumptions and data requirements for reliably estimating genetic parameters for the epidemiological host traits, as further discussed below.

The analytic equations also provide explicit expressions for the inter-dependency of PAs on the group size and number of groups, as well as the genetic relatedness between individuals, their allocation across epidemic contact groups, and the genetic and phenotypic trait covariance matrices. In conjunction with numerical results, these reveal key insights into how to estimate genetic parameters for these unobservable host traits, highlighting data requirements for future experimental or field studies. The analytical equations for the polygenic epidemiological traits thus complement our previously developed online Precision Calculator software (SIRE-PC) to aid power calculations for estimation of single genetic marker effects for these traits [35, 65].

As would be expected, PAs in the epidemiological traits increase with increasing trait heritability. Both numerical and analytical results show that the rate of increase is largest when trait heritability (and phenotypic variances) are small, and approach an asymptotic limit below 1 as heritability goes to 1. It is noteworthy, however, that in addition to dependence on the trait heritability, PAs for all three host epidemiological traits also depend on the phenotypic variances. In particular for host susceptibility and infectivity, these depend on phenotypic variances in both traits. This is firstly because the susceptibility, infectivity, (and also recoverability) phenotypes are not themselves directly measured, and secondly because whether an individual becomes infected depends on its own susceptibility as well as on the infectivity of its infected group members.

In addition to a dependence on trait heritability, our results also show that PAs can increase substantially if the traits are genetically correlated. This is particularly relevant for infectivity, for which low PA may hamper genetic improvement. As was demonstrated for the exemplar scenario, strong genetic correlations between infectivity and recoverability (the trait associated with highest PA in our study) can lead to more accurate estimates of genetic contributions to infectivity than susceptibility. In other words, depending on the genetic variances and correlations, it may be more effective to reduce infectious disease transmission in farmed animal populations through genetic improvement in recoverability or low infectivity rather than selecting for low susceptibility, which is the primary target trait in current breeding programmes.

Previous research has highlighted that estimation of genetic parameters for infectivity may require different field study or experimental designs to those currently used for quantitative genetic studies of disease data [34, 35]. Application of SIRE 2.0 here suggests that key factors are: the number of paternal half-sibs *P*, the contact group size, and to a lesser extent how related individuals are distributed across contact groups. In a real-world setting, the number of paternal half-sibs is very much dependent on the reproduction parameters and the production process for the species in question. For example, in cases in which artificial insemination is used (such as in the cattle industry), sires can have hundreds of offspring, in principle allowing for accurate estimation of additive genetic effects for all three epidemiological host traits [21, 66]. In line with previous results (e.g. [17, 29, 33]) group size was found to have a substantial, though opposite effect on PAs for host susceptibility and infectivity. Whereas PAs for susceptibility tend to be compromised when data comes from many small contact groups, the opposite is true for infectivity. This is because larger contact groups (when initial infective numbers are fixed) imply a greater fraction of initially susceptible individuals (which provide data regarding their susceptibility trait), but more uncertainty as to who is infecting whom. Furthermore, as individuals in contact groups become more and more related, PAs for susceptibility tend to decrease, whereas those for infectivity tend to increase. It should be noted, however, that contact groups must contain more than one family in order to disentangle genetic contributions for susceptibility and infectivity.

Finally, this study provides important insights regarding data requirements for estimating all three host epidemiological traits with adequate PA. Obviously, knowledge of the transmission routes between individuals, i.e. who infects whom, in addition to individuals’ infection and recovery times would be highly beneficial. Such knowledge is currently rarely available in real-world scenarios, although novel technological advances in, e.g., pathogen whole genome sequencing and barcoding, or contact tracing data could provide such valuable information in the near future [51, 67, 68]. This study thus assumed known infection and recovery times of individuals as the optimal observable disease phenotypes for estimating the epidemiological host traits, and investigated how increasing uncertainties affect PAs. Firstly, we found that time-censored data only resulted in a modest reduction in PAs, unless a substantial proportion of the epidemic is unobserved. In real-world scenarios some individuals may not become infected, recover or die within the observation period. Our results indicate that infection and recovery records on these individuals are not essential for obtaining good predictions.

In practice, individual infection times are also rarely known with precision. Encouragingly, the predictive ability of SIRE 2.0 was only marginally compromised when repeated observations of individual infection status based on diagnostic tests were used as observable disease phenotypes. Strikingly, a substantial reduction in prediction accuracies only became apparent when the time step between these observations was relatively large (of the order of the time for the epidemic as a whole). In line with previous studies, our research highlights the importance of longitudinal individual disease records for estimating host epidemiological traits, but that high sampling frequencies are not essential [17, 29, 30, 33]. Our results do show, however, a significant reduction in PAs, especially for susceptibility and infectivity, if only the recovery (or mortality) times of individuals are known. Such data are common, e.g., in aquaculture disease challenge experiments, where only individual mortality times can be reported because non-invasive in-vivo diagnostics are sparse [62]. However, as outlined above, this reduction in PAs can be partly mitigated by the appropriate experimental design and large populations of related individuals, which may be easier to achieve in aquaculture than terrestrial farmed animal species.

## Limitations and future work

Although the focus of this study has been primarily on farmed animals, the methods introduced here are also applicable to infectious diseases in humans or plant species, for which individual disease records and genetic data exist. It should be noted, however, that the methodology rests on three fundamental assumptions which may not always hold in practice, and where substantially violated would require further method development:

Firstly, epidemic groups are assumed closed. This assumption can be easily met in disease challenge experiments, but may be more difficult to achieve in field settings, unless herd closures or movement restrictions are in place. Extension of the methodology to incorporate transmission between contact groups is relatively straight-forward. However, as indicated by the detrimental effects of increasing group size on PAs for infectivity observed in this study, one would expect that the ability to obtain reliable genetic parameter estimates for infectivity would be severely compromised.

Secondly, it was assumed that individuals mix homogeneously within contact groups. Whilst this may be a valid assumption for farmed animals housed in pens, modelling of more complex contact patterns would be more likely required for human and plant data (where contact is spatially determined). A key point is that contact structure is likely to be influenced by the relatedness of individuals and other genetic effects affecting individuals’ social behaviour (e.g. [69, 70]). Importantly, heterogeneities in contact structure can have a strong impact on disease transmission dynamics [71, 72] and thus likely affect genetic parameter estimates for host susceptibility and infectivity. Insights into individuals’ contact network, e.g. through individual movement records, may facilitate estimation of these traits.

Thirdly, it was assumed that the dynamics of the epidemics can be adequately represented by an epidemiological SIR model*.* Although this is valid for a large range of diseases [73], extension of the methodology to accommodate more complex epidemiological models is currently ongoing. For example, in many cases infected individuals enter an ‘exposed’ infected state before they become infectious. For other types of diseases, accumulation of pathogen in the environment, or disease vectors, such as insects, as primary routes of transmission may need to be incorporated into the genetic-epidemiological models and inference methods.

The methodology introduced in this paper focuses on estimation of genetic parameters for host susceptibility, infectivity and recoverability. In principle these can be used in genome wide association studies (GWAS) to investigate the genetic architecture of these traits when genomic information on individuals is available. This is yet to be explored, and may facilitate genomic selection or discovery of novel genetic loci or candidate genes associated with disease transmission.

In summary, the methods introduced in this study highlight the potential of longitudinal disease records of individuals coupled with genetic data for estimating individuals’ full genetic contributions to disease transmission. Rapid advances in affordable in-vivo diagnostic tests, increased use of video surveillance on farms, coupled with artificial intelligence software, may allow for the real-time detection of transmission routes and animal’s infection and recovery status to become a reality in the not-too-distant future [74]. Wide-spread adoption of such approaches, and advances in genomic technologies, would lead to huge amounts of data that could be leveraged by future versions of SIRE.

## Conclusions

Genetic selection to reduce individual susceptibility, infectiousness and infectious duration promises an effective and long-lasting solution to diminish the incidence and impact of infectious diseases in farmed animals. This paper introduces a new Bayesian methodology and software tool (SIRE 2.0) to simultaneously estimate variance components and additive genetic contributions for these traits from a range of epidemiological data that can be readily collected in experimental or field studies. A simulation study and analytical expressions reveals how the accuracy of these trait estimates depend on population and contact group structure, providing insights for study design and future research.

## Supplementary Information

Additional file 1. Addition of a SNP into the model. How SNPs can be incorporated into the genetic-epidemiological model.

Additional file 2. Bayesian prior. Prior assumptions for model parameters.

Additional file 3. MCMC procedure. Outlines how Markov chain Monte Carlo is used to generate posterior samples.

Additional file 4. SIRE2.0 software tool. Briefly describes the SIRE2.0 software.

Additional file 5. SIRE2.0 user manual. How to use the SIRE2.0 software.

Additional file 6. Analytic expressions for prediction accuracies. Shows the derivation of mathematical expressions for prediction accuracy from the posterior.

Additional file 7. Simulating epidemics. How simulated data is generated using the modified Doob-Gillespie algorithm.

Additional file 8. Extension to other population structures. Looks at how PAs change under inclusion of full-sibs or related sires.

Additional file 9. Interpreting phenotypic variances and modelling super-spreaders. This section aims to interpret the values used for phenotypic variances and introduces the 80/20 rule.

Additional file 10. Expression of prediction accuracies for a simple paternal half-sib model. Here the complex prediction accuracies are simplified by assuming a paternal half-sib model.

Additional file 11. The incorporation of random group or fixed effects. Investigates the impact of random group or fixed effects on prediction accuracies for the epidemiological host traits.

## Data Availability

The software tool developed and used in this paper is accessible from the GitHub repository https://github.com/theITEAM/SIRE2.0. A snapshot of this repository used in the analysis is deposited on Zenodo [75].

## Footnotes

1 Note, alternatives to the gamma distribution could be the log-normal or Weibull distributions, if they provide a closer fit to the disease under study.

2 “Fractional deviation” is defined by the exponential dependency in Eq. (1), e.g.* g*_*j*_ = 0.1 corresponds to individual *j* being a fraction ≈ 10% more susceptible than a population-wide reference.

3 These include infection and recovery events before observations start and recoveries after observations end. To improve computational efficiency, infection events after observations end are not included, as these have no impact on the posterior.

4 Consequently, most individuals express their susceptibility, infectivity and recoverability phenotypes.

5 i.e. s doesn’t include index cases or individuals not taking part in epidemics (e.g. sires) because they don’t provide information about their susceptibility.

6 In contrast *k* = 1 would correspond to an exponentially distributed infection duration, as is commonly used in Markovian models.

7 For the baseline model each sire *n* has the same prediction accuracy, so it makes sense to define the PAs on the sire level.

8 Because they are population-level parameters, rather than individual-level, so increasing data necessarily leads to smaller uncertainties.

9 Note, it is not necessary for all individuals to become infected, and for cases in which the basic reproduction number *R*_*0*_ approaches one, the proportion of uninfected individuals becomes high.

10 These “replicates” are each like that shown in Fig. 1, but stochastically different due to the pseudo-random number generator being initialised with a different seed. As described in the Methods section, the actual data used by SIRE 2.0 is the infection status of individuals taken at periodic time intervals.

11 A result which can be obtained from Eq. (11) by taking the limit when Ω_*gg*_ and Θ_*gg*_ become large, such that other terms 1 and Θ_*ff*_/*N* become negligible in the denominator, but the ratio between the two, which gives the heritability ${h}_{g}^{2}$, is fixed.

12 This is because fewer individuals are index cases leading to more information regarding susceptibility.

13 As shown in Additional file 9, the frequently stated 80/20 rule, in which 20% of individuals cause 80% of infections, corresponds to a large phenotypic variance in infectivity of Θ_*ff*_ ≈2.8.
