# Supplementary material for: SIRE 2.0: a novel method for estimating polygenic host effects underlying infectious disease transmission, and analytical expressions for prediction accuracies
Source: Genet Sel Evol. 2025 Apr 1;57:17. doi: 10.1186/s12711-025-00956-4 (PMC11963337; doi:10.1186/s12711-025-00956-4)
Supplement: Supplementary file 1 — Additional file 1. Addition of a SNP into the model. How SNPs can be incorporated into the genetic-epidemiological model. [file 12711_2025_956_MOESM1_ESM.pdf]

Addition of SNP to the model

Along with the other terms in Eq.(2), SIRE allows for incorporation of a single SNP effect. This SNP is assumed to be bi-allelic with values *A* and *B*. When SNP data is added the model becomes

$$\begin{aligned} \boldsymbol{g} &= \boldsymbol{g}^{\mathrm{SNP}} + \boldsymbol{X}\boldsymbol{b}_g + \boldsymbol{a}_g + \boldsymbol{\varepsilon}_g, \\ \boldsymbol{f} &= \boldsymbol{f}^{\mathrm{SNP}} + \boldsymbol{X}\boldsymbol{b}_f + \boldsymbol{a}_f + \boldsymbol{\varepsilon}_f, \\ \boldsymbol{r} &= \boldsymbol{r}^{\mathrm{SNP}} + \boldsymbol{X}\boldsymbol{b}_r + \boldsymbol{a}_r + \boldsymbol{\varepsilon}_r, \end{aligned} \tag{A1}$$

where $\boldsymbol{g}^{\mathrm{SNP}}$, $\boldsymbol{f}^{\mathrm{SNP}}$ and $\boldsymbol{r}^{\mathrm{SNP}}$ give contributions from the SNP under investigation. These terms are parameterised by

$$g_j^{\mathrm{SNP}} = \begin{matrix} h_g \\ h_g\Delta_g \\ -h_g \end{matrix}, \quad f_j^{\mathrm{SNP}} = \begin{matrix} h_f \\ h_f\Delta_f \\ -h_f \end{matrix}, \quad r_j^{\mathrm{SNP}} = \left.\begin{matrix} h_r \\ h_r\Delta_r \\ -h_r \end{matrix}\right\} \begin{matrix} \text{if } j \text{ is } AA \\ \text{if } j \text{ is } AB \\ \text{if } j \text{ is } BB \end{matrix} \tag{A2}$$

where h_g, $h1$ and h_r give half the difference in traits between the *AA* and *BB* homozygote genotypes and Δ_g, Δ_f and Δ_r represent the degree of dominance (a value of 1 corresponds to complete dominance of the *A* allele over the *B* allele and -1 when the reverse is true, whereas absence of dominance is represented by a value of 0) [74].
