# Supplementary material for: SIRE 2.0: a novel method for estimating polygenic host effects underlying infectious disease transmission, and analytical expressions for prediction accuracies
Source: Genet Sel Evol. 2025 Apr 1;57:17. doi: 10.1186/s12711-025-00956-4 (PMC11963337; doi:10.1186/s12711-025-00956-4)
Supplement: Supplementary file 2 — Additional file 2. Bayesian prior. Prior assumptions for model parameters. [file 12711_2025_956_MOESM2_ESM.pdf]

Bayesian prior

The prior can be decomposed into

$$\pi(\theta)=\pi(\theta_{-\boldsymbol{a},\varepsilon,\boldsymbol{G}})\pi(\boldsymbol{a}\,|\,\Omega)\pi(\boldsymbol{\varepsilon}\,|\,\Psi)\pi(\boldsymbol{G}\,|\,\sigma_{GE}), \tag{A1}$$

where $\theta_{-\boldsymbol{a},\boldsymbol{\varepsilon},\boldsymbol{G}}$ includes all parameters with the exception of $\boldsymbol{a}$, $\boldsymbol{\varepsilon}$ and $\boldsymbol{G}$.

The priors for the additive genetic and environmental contributions are given by multivariate normal distributions:

$$\pi(\boldsymbol{a}\,|\,\Omega)=\frac{1}{\sqrt{(2\pi)^{2N_{\text{total}}}|\mathrm{A}||\Omega|}}e^{-\frac{1}{2}\sum_{t,m,n,u}a_{t,m}\mathrm{A}^{-1}_{mn}\Omega^{-1}_{tu}a_{u,n}},$$
$$\pi(\boldsymbol{\varepsilon}\,|\,\Psi)=\frac{1}{\sqrt{(2\pi)^{2N_{\text{total}}}|\Psi|}}e^{-\frac{1}{2}\sum_{t,m,u}\varepsilon_{t,m}\Psi^{-1}_{tu}\varepsilon_{u,m}}. \tag{A2}$$

where t and u sum over trait type (*i.e. g*, *f* and *r*) and m and n sum over all N_{total} individuals. The 3×3 covariance matrices $\boldsymbol{\Omega}$ and $\boldsymbol{\Psi}$ describe not only the overall magnitude of the additive genetic and environmental contributions, but also any potential correlations between these traits. The known relationship matrix $\mathbf{A}$ (determined either by a pedigree or through a genomic relationship matrix) determines trait correlations between individuals.

Finally, the prior for the group effects is given by

$$\pi(\boldsymbol{c}\,|\,\sigma_c)=\prod_z\frac{1}{\sqrt{2\pi}\sigma_c}e^{-\frac{1}{2\sigma_c^2}c_z^2}, \tag{A3}$$

where the product z goes over all contact groups and c_z represents the group-based fractional deviation in transmission rate (see Eq.(1)), which is assumed to be independent between groups and normally distributed with standard deviation σ_c.

In this study the prior for $\theta_{-\boldsymbol{a},\boldsymbol{\varepsilon},\boldsymbol{c}}$ (which can be modified in SIRE 2.0 if necessary) is largely uninformative but does place upper and lower bounds on many of the key parameters to stop them straying into biologically unrealistic values:

$$\begin{array}{lll}
 & \Omega_{gg}\sim\text{Uniform}(0,3), & \Psi_{gg}\sim\text{Uniform}(0,3),\\
\beta\sim\text{Uniform}(0,1), & \Omega_{ff}\sim\text{Uniform}(0,3), & \Psi_{ff}\sim\text{Uniform}(0,3),\\
\gamma\sim\text{Uniform}(0,1), & \Omega_{rr}\sim\text{Uniform}(0,3), & \Psi_{rr}\sim\text{Uniform}(0,3),\\
k\sim\text{Uniform}(1,5), & \Omega_{gf}\sim\text{Uniform}(-3,3), & \Psi_{gf}\sim\text{Uniform}(-3,3),\\
\sigma_c\sim\text{Uniform}(1,1), & \Omega_{gr}\sim\text{Uniform}(-3,3), & \Psi_{gr}\sim\text{Uniform}(-3,3),\\
 & \Omega_{fr}\sim\text{Uniform}(-3,3), & \Psi_{fr}\sim\text{Uniform}(-3,3).
\end{array} \tag{A4}$$
