# Supplementary material for: SIRE 2.0: a novel method for estimating polygenic host effects underlying infectious disease transmission, and analytical expressions for prediction accuracies
Source: Genet Sel Evol. 2025 Apr 1;57:17. doi: 10.1186/s12711-025-00956-4 (PMC11963337; doi:10.1186/s12711-025-00956-4)
Supplement: Supplementary file 3 — Additional file 3. MCMC procedure. Outlines how Markov chain Monte Carlo is used to generate posterior samples. [file 12711_2025_956_MOESM3_ESM.pdf]

MCMC procedure

Markov chain Monte Carlo (MCMC) produces a list of (correlated) parameter θ^q and event time ξ^q samples drawn from the posterior probability distribution in Eq.(3), where q indexes sample number.

This file describes a simple adaptive MCMC procedure for generating these samples.

1) Initialisation – MCMC is initially started with some set of parameters θ^1 and event times ξ^1 consistent with the data y. In the case of parameters, θ^1 is simply sampled from the prior distribution $\pi(\theta)$. For events, initialisation depends on the available data: (1) If any event times are known they are set in ξ^1 , (2) when recovery times are known, unmeasured infection times are exponentially sampled backwards in time from the observed recovery time[1], (3) when infection times are known, recovery times are exponentially sampled forwards in time from the observed infection time[2], (4) when the disease status of individuals is periodically checked, infection and recovery times are sampled uniformly in the time intervals identified between successive diagnostic tests.

2**) Iteration** - MCMC operates by proposing changes to the model parameters θ and event times ξ and accepting or rejecting these changes in accordance with a Metropolis-Hastings probability. In this way the underlying dynamics are able to explore all potential possibilities consistent with the observations.

A single MCMC "update" consists of making the following set of proposals:

MCMC UPDATE

Individual parameter proposals – Each individual parameter in $\theta = \{\beta, \gamma, k, \mathbf{b_g}, \mathbf{b_f}, \mathbf{b_r}, \mathbf{a_g}, \mathbf{a_f}, \mathbf{a_r}, \mathbf{\Omega}, \boldsymbol{\varepsilon_g}, \boldsymbol{\varepsilon_f}, \boldsymbol{\varepsilon_r}, \mathbf{\Psi}, \mathbf{c}, \sigma_c\}$, denoted by θ_j, is considered in turn. A proposed value is drawn from a normal distribution centred on the parameter's current chain value

$$\theta_j^p \sim Norm(\theta_j^q, J_j^2), \qquad \text{(A1)}$$

with all other parameters in θ^p remaining the same as in θ^q (if this produces an inconsistent value, *e.g.* β becomes negative, the proposal is immediately rejected). The proposal is accepted with Metropolis-Hastings probability

$$P_{MH} = \min\left(1, \frac{L(\xi^p|\theta^p)\pi(\theta^p)}{L(\xi^q|\theta^q)\pi(\theta^q)}\right). \qquad \text{(A2)}$$

If accepted, we set $\theta^{q+1} = \theta^p$ else $\theta^{q+1} = \theta^q$ with $\xi^{q+1} = \xi^q$.

Tuning J_j in Eq.(A1) is important. If it is too large, very few proposals will be accepted and if too small, mixing will be slow. Motivated by adaptive MCMC [1, 2], a robust heuristic method for optimising J_j within the burn-in period is as follows. Initially, J_j is set to a small quantity. Each time a proposed change on parameter j is accepted J_j is updated according to

$$J_j^{new} = J_j \times 1.01, \qquad \text{(A3)}$$

[1] The standard deviation in recovery times is used to set the decay rate.
[2] The standard deviation in infection times is used to set the decay rate.

and when rejected

$$J_j^{new} = J_j \times 0.995. \tag{A4}$$

These numerical factors are chosen for two reasons: Firstly, the updates in Eqs.(A3) and (A4) balance each other out when acceptance occurs around 33% of the time, leading to a steady state solution for J_j. Secondly, they are chosen to be sufficiently close to *1* to prevent large fluctuations in J_j, but sufficiently far to allow the steady state solution to be found within the burn-in period.

Joint parameter proposals – In addition to the single parameter proposals outlined above, joint proposals are also made on additive genetic contributions and residuals along with their corresponding covariance matrices. The reason these joint updates are necessary is that these quantities are often highly correlated within the model which can lead to extremely slow mixing.

Proposals consist of randomly selecting a trait, *e.g.* susceptibility, and then proposing a change along the diagonal of the covariance matrix:

$$\Omega_{gg}^p \sim Norm(\Omega_{gg}^q, J_g^2). \tag{A5}$$

This defines a scaling factor

$$f = \sqrt{\frac{\Omega_{gg}^p}{\Omega_{gg}^q}}, \tag{A6}$$

which is used to generate proposals for the additive genetic effects and other elements in the covariance matrix:

$$\begin{aligned} &\boldsymbol{a}_g^p = f\boldsymbol{a}_g^q, \\ &\Omega_{gf}^p = f\Omega_{gf}^q, \quad \Omega_{gr}^p = f\Omega_{gr}^p, \\ &\Omega_{fg}^p = f\Omega_{fg}^p, \quad \Omega_{rg}^p = f\Omega_{rg}^p. \end{aligned} \tag{A7}$$

The proposal is accepted with probability:

$$P_{MH} = \min\left(1, \frac{L(\xi^p|\theta^p)\pi(\theta^p)}{L(\xi^q|\theta^q)\pi(\theta^q)} \frac{1}{f^{N+2}}\right), \tag{A8}$$

where N is the number of individuals in the system. As above, the value for J_j is tuned to give and acceptance rate of around 33%.

Event proposals – In situations in which ξ is not precisely known, the unknown latent event times must be stochastically changed in accordance with the posterior distribution. Each individual j is considered in turn. In the case of proposing changes to the infection time of j, the following normal distribution is sampled from:

$$t_j^{\mathrm{I},p} \sim Norm(t_j^{\mathrm{I},q}, J_{I,j}^2), \tag{A9}$$

with all other event times in $\xi^p=(t^I,t^R)$ remaining the same as in ξ^q. If this proposed infection time exceeds the recovery time $t_j^{R,q}$ the proposal is immediately rejected (infections cannot occur after recoveries). The proposal in Eq.(A9) is accepted with probability

$$P_{MH} = \min\left(1, \frac{\pi\left(y|\xi^p\right)L(\xi^p|\theta^p)}{\pi\left(y|\xi^q\right)L(\xi^q|\theta^q)}\right). \quad \text{(A10)}$$

If accepted, we set $\xi^{q+1}=\xi^p$ else $\xi^{q+1}=\xi^q$ with $\theta^{q+1}=\theta^q$.

A similar proposal to Eq.(A9) is used to change recovery times. The jumping parameters $J_{I,j}$ and $J_{R,j}$ are again tuned to give an average acceptance probability of around 33% (using the same method as in Eqs.(A3) and (A4) above). Furthermore, there are data scenarios in which it is not possible to tell whether an individual has become infected or not. In these cases it is necessary to include additional proposals which insert infection/recovery event pairs and, conversely, other proposals which remove them.

Each of the proposals above have been optimised to calculate only those parts of the likelihood that change, *e.g.* if the infection time of individual *j* is altered then only the change in likelihood in the period between then initial and proposed times within *j*'s contact group needs to be calculated. Furthermore, when making some sets of proposals computational speed is substantially increased by pre-calculating terms in the likelihood which remain unchanged.
