# Supplementary material for: SIRE 2.0: a novel method for estimating polygenic host effects underlying infectious disease transmission, and analytical expressions for prediction accuracies
Source: Genet Sel Evol. 2025 Apr 1;57:17. doi: 10.1186/s12711-025-00956-4 (PMC11963337; doi:10.1186/s12711-025-00956-4)
Supplement: Supplementary file 4 — Additional file 4. SIRE2.0 software tool. Briefly describes the SIRE2.0 software. [file 12711_2025_956_MOESM4_ESM.pdf]

## SIRE 2.0 software tool

This desktop application reads in epidemiological data, user-specified fixed or random effects, and pedigree-based or genomic relationship matrices, and generates posterior estimates for parameters $\theta$ from the genetic-epidemiological model using the Bayesian inference methodology outlined in the methods section of the main paper. It is freely available to download from github.com/theITEAM/SIRE2.0 (with versions for Windows, Linux and Mac). The version used to generate the results in this paper has been placed into a Zenodo repository [42], and many of the simulated datasets have been implemented as illustrative examples for training purposes (these are linked to from within the software, with the raw files available in the 'Examples' and 'Datasets' directories).

An easy-to-use interface allows for data tables to be imported in a variety of formats. SIRE 2.0 takes as input any combination of information about individuals' infection times, recovery times, disease status measurements and disease diagnostic test results. Within the model the genotypes of SNPs or other fixed effects along with details of which individuals belong to which contact groups can be specified. The relationship matrix **A,** or it's inverse, can be loaded (either in raw matrix form, which could be used to implement a genomic relationship matrix, or by specifying a pedigree) which allows for the estimation of additive genetic effects, *i.e.* $\boldsymbol{a}_g$, $\boldsymbol{a}_f$ and $\boldsymbol{a}_r$ in Eq.**Error! Reference source not found.**. A range of prior specifications can be made on model parameters (those used in this study are outlined in Additional file 2).

The outputs from SIRE 2.0 consist of posterior trace plots for model parameters, distributions, visualisation of infection and recovery times, dynamic population estimates and summary statistics (means and 95% credible intervals) as well as MCMC diagnostic statistics. Posterior distribution graphs can be exported, as well as files containing posterior samples of parameters and events (for potential further analysis using other tools). The user guide for SIRE 2.0 is available in Additional file 5 as well as on GitHub.

Efforts are currently underway to incorporate the Bayesian methodology presented in this paper into a more widely applicable software tool called BICI (Bayesian Individual-based Compartmental Inference), which allows for greater flexibility in model definition as well as supporting a richer array of data types.
