# Supplementary material for: SIRE 2.0: a novel method for estimating polygenic host effects underlying infectious disease transmission, and analytical expressions for prediction accuracies
Source: Genet Sel Evol. 2025 Apr 1;57:17. doi: 10.1186/s12711-025-00956-4 (PMC11963337; doi:10.1186/s12711-025-00956-4)
Supplement: Supplementary file 5 — Additional file 5. SIRE2.0 user manual. How to use the SIRE2.0 software. [file 12711_2025_956_MOESM5_ESM.pdf]

SIRE

SIRE 2.0

Susceptibility, Infectivity and Recoverability Estimation

Christopher M. Pooley[1,2*], Glenn Marion[1&], Jamie Prentice[2], Ricardo Pong-Wong[2], Stephen C. Bishop† and Andrea Doeschl-Wilson[2&]

[1] Biomathematics and Statistics Scotland, James Clerk Maxwell Building, The King's Buildings, Peter Guthrie Tait Road, Edinburgh, EH9 3FD, UK.

[2] The Roslin Institute, The University of Edinburgh, Midlothian, EH25 9RG, UK.

† Deceased

Table of Contents

1 Introduction 3

1.1 Downloading 3

1.2 Getting started 5

2 Inputs 5

2.1 Description 5

2.2 The data 7

2.3 The model 11

2.4 The priors 14

3 Outputs 15

3.1 Starting inference 15

3.2 Trace plots 16

3.3 Probability distributions 18

3.4 Scatter plot 18

3.5 Individual timelines 19

3.6 Statistics 19

3.7 Population plots 21

3.8 Exporting 21

3.9 Loading and saving 21

4 Examples 22

4.1 SIR model 22

4.2 SI model 23

5 Code 24

6 License and warranty 24

7 Citing SIRE 24

8 Acknowledgments 24

References 25

1 Introduction

Three key epidemiological host traits affect infectious disease spread: susceptibility (propensity to acquire infection), infectivity (propensity to transmit infection to others, once infected) and recoverability (propensity to recover quickly). SIRE 2.0 is a desktop application for estimating factors affecting these three traits based on epidemiological data.

The term "contact group" refers to individuals sharing the same environment, such as a pasture, pen, cage, tank or pond. SIRE 2.0 applies to individual-level disease data originating from one or more contact groups in which infectious disease is transmitted from infectious to susceptible individuals through effective contact (for simplicity it is assumed that groups are closed, *i.e.* no births, migrations, or disease transmission between groups). Data can come from well controlled disease transmission experiments or from much less well controlled field data (which may be less complete, but readily available in greater quantity).

SIRE 2.0 takes as input any combination of information about infection times, recovery times, disease status measurements, disease diagnostic test results, genotypes at a particular SNP[1] under investigation (if applicable) or any other fixed effects, details of which individuals belong to which contact groups, a pedigree, a (potentially genomic) relationship matrix and any prior specifications.

The output from SIRE 2.0 consists of posterior trace plots for model parameters, distributions, visualisation of infection and recovery times, dynamic population estimates and summary statistics (means and 95% credible intervals) as well as MCMC diagnostic statistics.

A detailed description of the epidemiological and statistical model underlying SIRE 1.0 along with the Bayesian inference methodology is given in an accompanying paper [1] (note, a paper looking at additive genetic effects used in SIRE 2.0 is in preparation). The focus of this manual is on the practicalities of analysing real world data and interpreting the results.

1.1 Downloading

SIRE 2.0 is freely available under the GNU General Public License, and can be downloaded from GitHub: https://github.com/theITEAM/SIRE2.0.

Depending on your platform, the following instructions explain how SIRE 2.0 can be run:

- **Windows** – Download the file "SIRE_v2.0_windows.zip" and unzip. SIRE 2.0 is run by clicking on the "SIRE2.exe" icon.
- **Linux** – Download the file "SIRE_v2.0_linux.tar.gz". This can then be extracted by using the terminal command "tar -zxvf SIRE_v2.0_linux.tar.gz". The code is executed using "./SIRE2".
- **Macintosh** – Download the file "SIRE_v2.0_Mac.zip". SIRE is run by clicking on the "SIRE.app" icon (if the error message "SIRE can't be opened because it is from an unidentified developer…" appears, right clicking on "SIRE2.app" and selecting "Open" will allow the option to run).

[1] SNP stands for "single nucleotide polymorphism" and refers to particular locations along the genome that exhibit a large degree of variability across the population.

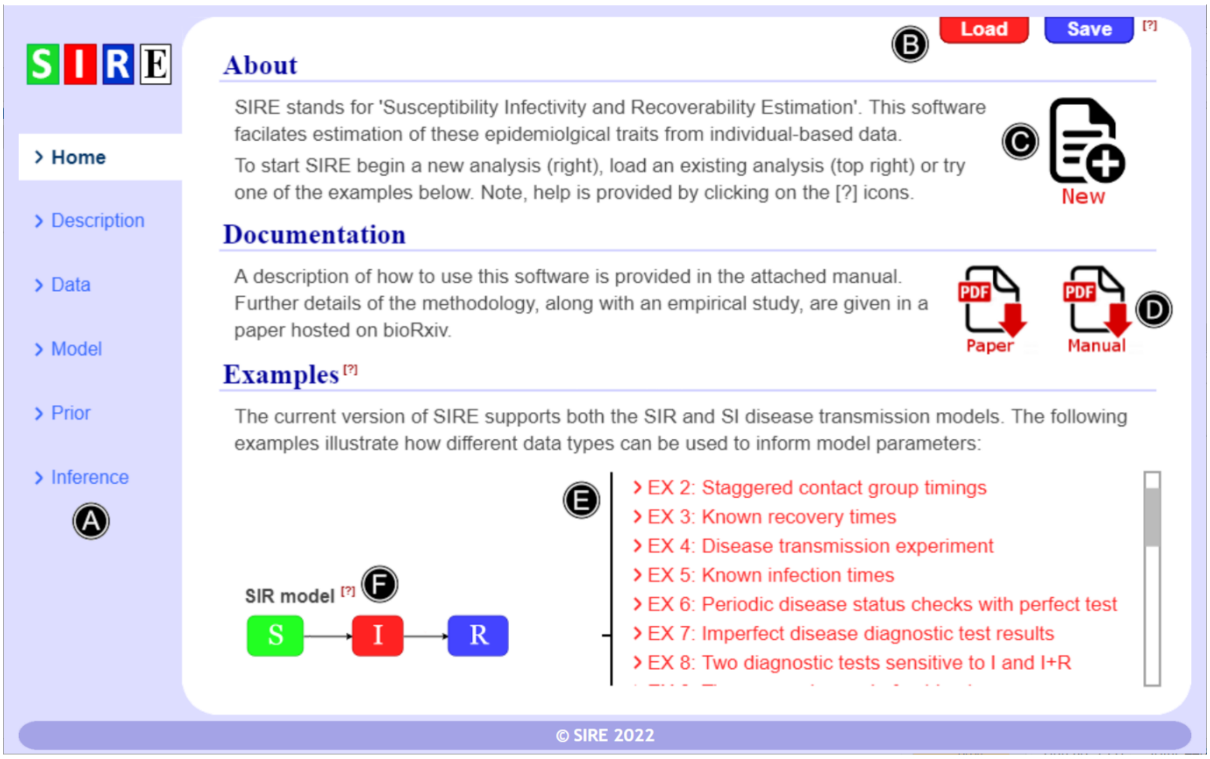

Figure 1 – *The home screen*. A: Main menu, B: load previous analysis or save current analysis, C: start a new analysis, D: link to the manual and paper, E: examples looking at various data scenarios (see section 4), F: Click on [?] for more information (this can be done for most properties on the page).

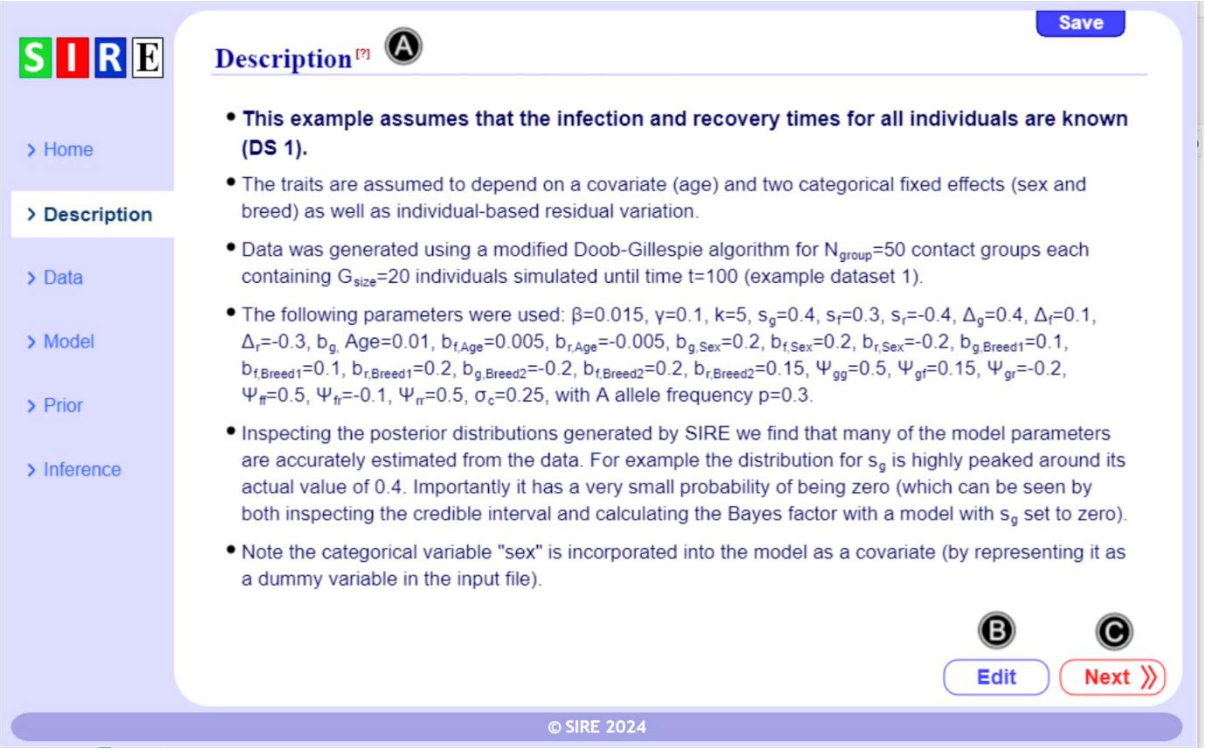

Figure 2 – *Description*. A: Text panel showing a description of the data and model assumptions (here for EX.1); B: edit this description, C: go to the next step. Parameter definitions are given in Table 1.

1.2 Getting started

Figure 1 shows the home screen displayed when SIRE 2.0 is first loaded. The main menu on the left (Fig. 1A) is used to navigate arbitrarily from page to page. To begin three options are available: a previous analysis can be loaded (Fig. 1B, note SIRE 2.0 uses a special ".sire" file format for loading and saving analyses, as described in section 3.9), a new analysis can be started (Fig. 1C), or one of the illustrative examples can be investigated (Fig. 1E). The examples refer to different data scenarios (DS) that SIRE 2.0 can handle (see section 4). New users are encouraged to try these first and spend some minutes exploring the software to get a feeling for how it works. Successively clicking "Next" goes through the various data and model options (discussed in detail below), and starting inference leads to several visualisations for the posterior. These examples can be modified (*e.g.* by making changes to the model/data), but the default settings are restored when reloaded from the home screen in Fig. 1.

Additional information on many of the screens can be gained by clicking on the [?] buttons (*e.g.* Fig. 1F). This manual follows the order of items on the main menu (Fig. 1A).

2 Inputs

This section describes how data is incorporated into SIRE 2.0, along with specification of the model and prior[2].

2.1 Description

As shown in Fig. 2A, SIRE 2.0 allows users to provide a brief description of the data and assumptions used for analysis. This is not only useful to keep track for personal use, but also makes it easier and more transparent for others to understand what has been done. The description can be edited by clicking on Fig. 2B (note, bullet points are automatically generated for each carriage return in the editable text box). Complete these details and click "Next" at the bottom right of the screen (Fig. 2C). Note, the next button on each of the pages is just for convenience. In fact the menu on the left hand side can be used to navigate to any page without loss of information.

---

[2] Priors encapsulate previous knowledge regarding model parameters and ensure they are confined to physically realistic values during Bayesian inference.

	A	B	C	D	E	F	G	H	I
1	ID	Group	SNP	It	Rt	Age	Sex	Breed	Type
2	ind0	Gr 1	AB	0	8.52943	39	1	Breed 1	Seeder
3	ind1	Gr 1	AB	7.46777	17.5333	36	0	Breed 2	Contact
4	ind2	Gr 1	AA	3.26406	44.8453	39	0	Breed 3	Contact
5	ind3	Gr 1	AA	3.02582	12.8424	49	0	Breed 2	Contact
6	ind4	Gr 1	AA	3.7433	18.6858	37	0	Breed 3	Contact
7	ind5	Gr 1	AB	3.84091	12.0541	32	0	Breed 1	Contact
8	ind6	Gr 1	AA	4.53991	21.1796	40	1	Breed 3	Contact
9	ind7	Gr 1	AB	3.93424	9.49594	33	0	Breed 3	Contact
10	ind8	Gr 1	AB	2.73098	17.4823	20	0	Breed 1	Contact
11	ind9	Gr 1	AA	12.3191	23.9068	33	1	Breed 2	Contact
12	ind10	Gr 1	AB	3.55243	11.9158	22	1	Breed 2	Contact
13	ind11	Gr 1	AB	3.2593	12.9687	44	0	Breed 2	Contact
14	ind12	Gr 1	AA	3.75865	20.2027	44	0	Breed 1	Contact
15	ind13	Gr 1	AB	3.24161	6.01077	32	0	Breed 3	Contact
16	ind14	Gr 1	AB	2.00271	50.6246	47	1	Breed 3	Contact
17	ind15	Gr 1	AA	2.07932	19.6202	21	1	Breed 1	Contact
18	ind16	Gr 1	AB	9.02055	32.4941	28	1	Breed 3	Contact
19	ind17	Gr 1	AA	no	no	40	1	Breed 3	Contact
20	ind18	Gr 1	AA	3.386	8.68745	43	1	Breed 2	Contact
21	ind19	Gr 1	AA	32.4559	48.4037	27	0	Breed 1	Contact
22	ind20	Gr 2	AB	0	4.06551	29	0	Breed 1	Seeder
23	ind21	Gr 2	AA	15.5408	46.6454	41	1	Breed 2	Contact
		⋮		⋮			⋮		

Figure 3– *Data formatting*. This shows an example data table from a simulated disease transmission experiment (EX.1). The columns are defined as ID: a unique identifier for individuals, Group: the name of the contact group that individual belongs to, SNP: the genotype at a particular SNP, It: the observed infection time, Rt: the observed recovery time, Age: the age, Sex: where 1 implies male, Breed: breed of the individuals, and Type: set as “Seeder” if an individual is infected at the beginning of the transmission trial else “Contact”.

2.2 The data

First we describe how data needs to be formatted to be imported into SIRE 2.0. Figure 3 shows an example dataset (in fact the one used for EX.1), as displayed on a spreadsheet. SIRE 2.0 is flexible in terms of reading in different types of data, and so the user's data doesn't necessarily need to look like this. Importantly, however, SIRE 2.0 does rely on one column giving individual IDs. Other columns can relate to whatever data happens to be available (with possibly extraneous columns, such as "Type" in this particular example, which are ignored). Details of these different data types are discussed later. The spreadsheet needs to be saved in .csv or tab-delimited .txt format so that it can be read by SIRE 2.0 (an example of this is the "Dataset 1.txt" file in the Datasets directory).

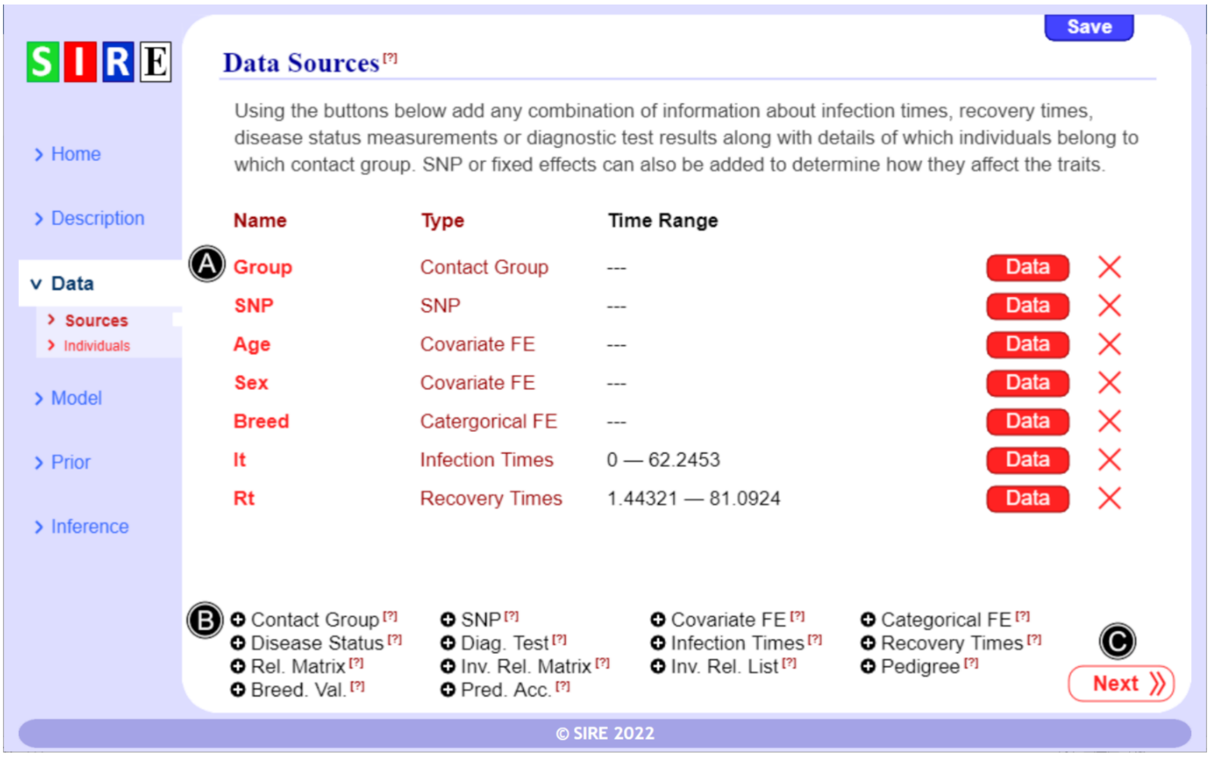

Figure 4– *Inputting data*. A: List of data sources, B: add new types of data, C: go to next page.

Next we discuss how information from this file is incorporated into SIRE 2.0. Rather than loading all the data at once, the user loads different sources of data one at a time (in any order). The data sources for EX.1 are shown in Fig. 4A, which shows information available about which individuals are in which contact groups, various fixed effects and the infection and recovery times for all individuals over a defined observation period.

Clicking on the buttons in Fig. 4B allows for various types of data to be added (whichever is appropriate):

- **Contact group** – Determine which individuals belong to which contact group. If this data is absent, it is assumed that all individuals share the same contact group.

- **SNP** – Provide the genotypes of individuals at a particular SNP under investigation (these must take one of the following possibilities: "AA", "AB", "BA" or "BB").
- **Covariate fixed effect (FE)** – Provide numerical covariate data (*e.g.* age) which potentially modifies the three epidemiological trait values. Here the outputted fixed effects represent regression slopes relating the traits to the data. Note, this approach can also be used to represent binary traits, *e.g.* 1 or 0 elements representing male or female (in which case the fixed effects represent sex-based differences in the traits).
- **Categorical fixed effect (FE)** – Data consisting of the category to which an individual belongs (*e.g.* breed). A reference category is selected (later) and the fixed effects represent the fractional change in the three epidemiological traits compared to this reference.
- **Disease status** – Data giving the infection status of individuals at particular points in time (these must take one of the following possibilities: "S", "I", "R" or "." if unknown).
- **Diagnostic test results** – Diagnostic test results at particular points in time (these must take the values "1" or "0" corresponding to positive or negative test results or "." if unknown). Additionally, the test has an associated sensitivity *Se* and specificity *Sp* which must be set. Note, tests can be selected to be sensitive to both the *I* and *R* states (*e.g.* appropriate for a serological test), or just the *I* state (*e.g.* appropriate for a culture test). Multiple sets of results from different diagnostic tests (*e.g.* ELISA/ γ-interferon / culture) can be incorporated into a single analysis.
- **Infection times** – Provide the times at which individuals become infected. If no infection is observed then the entry "no" is used and if unknown (*i.e.* unmeasured) "." is used. The range in time over which observations are actually made (allowing for censoring) is given in section 3.1.
- **Recovery times** – Provide the times at which individuals recover (or die in the case of disease induced mortality). If no recovery is observed then the entry "no" is used and if unknown "." is used. Again, the observation time range is given in section 3.1.
- **Relationship matrix** - This determines the relationship matrix $\mathbf{A}$, which gives the genetic similarity between different individuals in the system (either obtained from a pedigree or through a genomic relationship matrix). The user must load a table in which the heading line contains the IDs of individuals, and below this a square matrix gives the relationship matrix between individuals.
- **Inverse relationship matrix** – This determines $\mathbf{A}^{-1}$. The user must load a table in which the heading line contains the IDs of individuals, and below this a square matrix gives the inverse relationship matrix between individuals.
- **Inverse relationship matrix list** – If based on a pedigree, the inverse relationship matrix is often sparse (because it contains only non-zero elements for self and parent/sibling relationships). Saving in a sparse format can make file sizes much smaller. The following format is used: the first line gives a list of all individual ID which determines their order in the matrix. The subsequent lines contain three columns giving the x and y position in the matrix followed by the matrix element value (see Dataset 18 for an example). Note, because the matrix is symmetric only the upper triangular elements of the matrix need to be specified.
- **Pedigree** – Rather than specify a relationship matrix, here we specify the parents of each individual. The user loads a table with three columns giving the ID of individuals and their

two parents. If an individual's parent is unknown (or they are not being considered in the analysis) it is entered as '.'.

- **Breeding value** - These are the true values for additive genetic contributions (see $\boldsymbol{a_g}$, $\boldsymbol{a_f}$, $\boldsymbol{a_r}$ in Eq. (2)) to the traits (note, usually these are unknown unless the data has been simulated). Adding them as data allows for SIRE 2.0 to estimate prediction accuracies for the traits. The user must load a table with one column giving individual IDs and another the breeding values. Determining if the breeding value is for susceptibility, infectivity or recoverability is selected.
- **Prediction accuracy** – For a set of individuals, the prediction accuracy is the correlation between the true breeding value and the posterior mean of the estimate provided by inference (note, this can only be calculated if breeding values have been added to the analysis). Clicking this option defines the group of individuals over which the prediction accuracy is estimated. The user must load a table with a single column giving the IDs of individuals.

Note, not all of these data types are needed. For example in some cases only the death times of individuals are known (which are inputted as 'Recovery Times'), or in other scenarios only diagnostic test results are available. These various options are explored in the examples. Data can be viewed or edited by clicking on the red buttons in Fig. 4A, or deleted by clicking on the corresponding red crosses.

As noted above, many sources of data include a missing data option (represented by "."). However for the current version of SIRE, specified contact group, SNP and FE information is assumed to be precisely known.

After one of the possibilities in Fig. 4B is clicked, the user will be prompted to load the data file containing the required information, which will be .txt or .csv file from above. Once loaded the table is displayed on the screen, an example of which is shown in Fig. 5A. The user is prompted to select the key columns (in this example contact group information is being added) and click "Done" when complete. SIRE 2.0 also provides some basic data editing capabilities (Fig. 5B). These allow searching and replacing (useful, for example, when converting "+" and "-" to "1" and "0" for diagnostic test results), sorting and deleting (*e.g.* for removing missing data).

Once all the data sources have been added "Next" (Fig.4C) is clicked, which allows the user to view the data using the "Individuals" tab (Fig. 6). This shows timelines summarising temporal data for each of the individuals as well as contact group, SNP and fixed effect information.

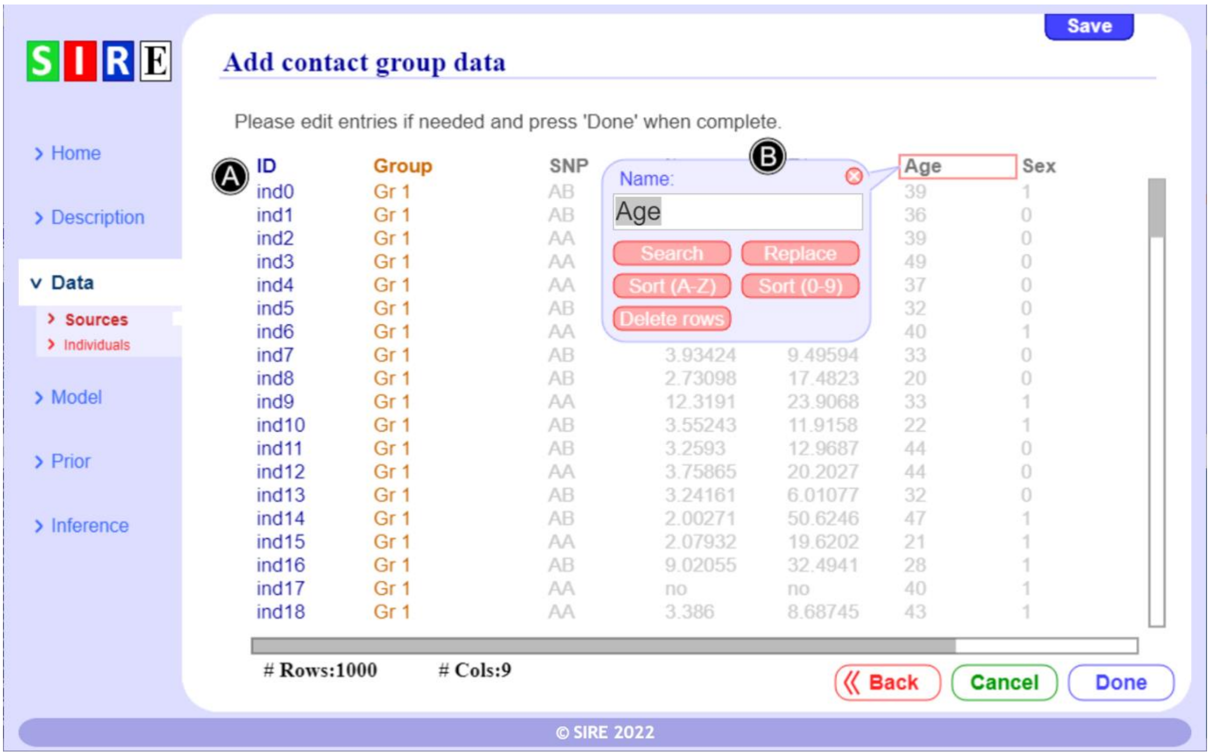

Figure 5 – Inputting *data tables*. A: Tables are loaded in .txt or csv format and the relevant data is then extracted, B: basic editing/manipulation of data can be performed.

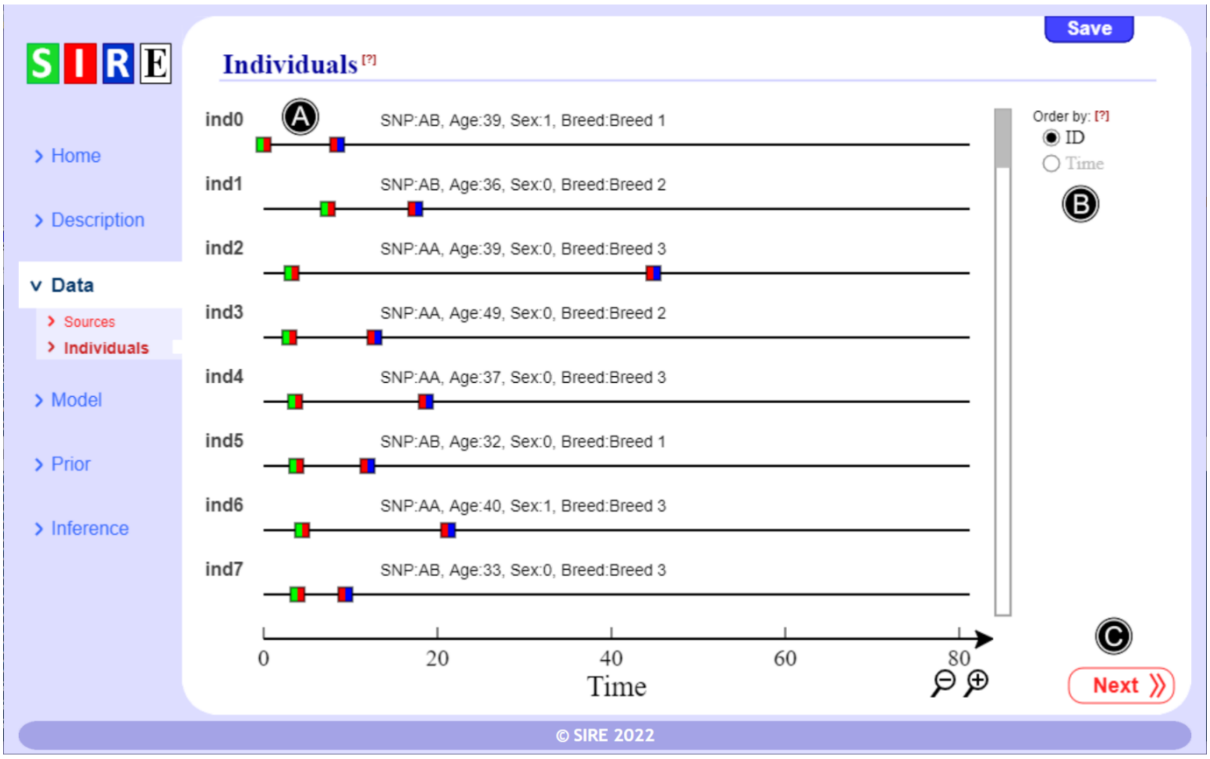

Figure 6 – *Viewing data*. A: Individual-based data (green/red or red/blue squares give infection and recovery times), B: filter by group and ordered by ID or time of first observation, C: go to next page.

2.3 The model

Here we define the underlying epidemiological and statistical model used to analyse the data. SIRE 2.0 generally assumes that the process of disease spread within a contact group follows an epidemiological SIR or SI model, where individuals' transition rates may be affected by various systematic (*e.g.* genotypic) and random effects. A comprehensive description is given in [1] which we briefly reiterate here for convenience. For the SIR model individuals are classified as being either susceptible to infection (*S*), infected and infectious (*I*), or recovered/removed/dead (*R*). The time-dependent force of infection for a susceptible individual *j* (*i.e.* the probability per unit time of becoming infected) is given by $\lambda_j(t)$. For those individuals that do become infected, the duration over which they are infectious is assumed to be gamma distributed with mean w_j and shape parameter k. These quantities can be expressed in the following way:

$$\lambda_j(t) = \beta e^{c_z} e^{g_j} \sum_i e^{f_i}, \qquad w_j = (\gamma e^{r_j})^{-1}, \tag{1}$$

where β and γ are population average transmission and recovery rates and c_z is a so-called "group effect" (z indexes contact group) that accounts for group-specific factors that influence the overall speed of an epidemic in one contact group relative to another (*e.g.* animals kept in different management conditions, environmental differences, or variation in pathogen strains with different virulence). c_z is assumed to be a random effect with standard deviation σ_c. Note, the expression for w_j does not contain a group effect as here it is assumed that the environment is the dominant mechanism affecting the speed at which infection spreads within groups rather than the individual's susceptibility, infectivity or recoverability.

In Eq.(1), g_j characterises the fractional deviation[3] in individual *j's* susceptibility as compared to that of the population as a whole, f_i characterises the corresponding quantity for individual *i's* infectivity, and r_j relates to recoverability. These, themselves, can be decomposed into various factors

$$\begin{aligned} \boldsymbol{g} &= \boldsymbol{g}^{\text{SNP}} + \boldsymbol{X}\boldsymbol{b}_g + \boldsymbol{a}_g + \boldsymbol{\varepsilon}_g, \\ \boldsymbol{f} &= \boldsymbol{f}^{\text{SNP}} + \boldsymbol{X}\boldsymbol{b}_f + \boldsymbol{a}_f + \boldsymbol{\varepsilon}_f, \\ \boldsymbol{r} &= \boldsymbol{r}^{\text{SNP}} + \boldsymbol{X}\boldsymbol{b}_r + \boldsymbol{a}_r + \boldsymbol{\varepsilon}_r, \end{aligned} \tag{2}$$

which are, respectively, a SNP effect $\boldsymbol{g}^{\text{SNP}}$, $\boldsymbol{f}^{\text{SNP}}$ and $\boldsymbol{r}^{\text{SNP}}$, fixed effects $\boldsymbol{b}_g$, $\boldsymbol{b}_f$ and $\boldsymbol{b}_r$, where $\mathbf{X}$ is a design matrix (*e.g.* to account for sex differences in the traits or vaccination status), additive genetic contributions $\boldsymbol{a}=(\boldsymbol{a}_g, \boldsymbol{a}_f, \boldsymbol{a}_r)$ that account for the relationship in trait values between different individuals (these are multivariate-normally distributed with zero mean and covariance matrix[4] $\mathbf{A}\otimes\boldsymbol{\Omega}$, where $\mathbf{A}$ is the pedigree/genomic relationship matrix and $\boldsymbol{\Omega}$ is a 3×3 covariance matrix that characterises potential correlations between traits). Finally, the residual contributions $\boldsymbol{\varepsilon}=(\boldsymbol{\varepsilon}_g,\boldsymbol{\varepsilon}_f,\boldsymbol{\varepsilon}_r)$ in Eq.(2) accounts for all other variation (these are also multivariate normal with zero mean and covariance matrix $\mathbf{I}\otimes\boldsymbol{\Psi}$, where $\mathbf{I}$ is the identity matrix reflecting the fact that residuals are assumed to be uncorrelated between individuals and $\boldsymbol{\Psi}$ is a 3×3 covariance matrix that characterises environmental correlations between traits).

[3] *E.g.* g_j=0.1 corresponds to individual *j* being ≃10% more susceptible than the population average.
[4] The symbol "⊗" is a tensor product, *e.g.* the covariance between $a_{g,i}$ (the genetic contribution for susceptibility for individual *i*) and $a_{f,j}$ (the genetic contribution for infectivity for individual *j*) is given by $A_{ij}\Omega_{gf}$.

If included, the SNP effect in Eq.(2) is itself dependent on the genotype of individuals (taken to be *AA*, *AB* or *BB* for diploid organisms, where *A* and *B* are two potential alleles at the genetic locus under investigation) in the following way

$$
{}_j^{SNP} = \begin{matrix} s_g \\ s_g\Delta_g \\ -s_g \end{matrix}, \quad f_j^{SNP} = \begin{matrix} s_f \\ s_f\Delta_f \\ -s_f \end{matrix}, \quad r_j^{SNP} = \left.\begin{matrix} s_r \\ s_r\Delta_r \\ -s_r \end{matrix}\right\} \begin{matrix} \text{if } j \text{ is } AA \\ \text{if } j \text{ is } AB \\ \text{if } j \text{ is } BB \end{matrix} \qquad (3)
$$

The parameters s_g, s_f and s_r capture the relative differences in trait values between *AA* and *BB* individuals and the scaled dominance factors Δ_g, Δ_f and Δ_r characterise the trait deviations between the heterozygote *AB* individuals and the homozygote mean (a value of 1 corresponds to complete dominance of the *A* allele over the *B* allele and -1 when the reverse is true).

Table 1 summarises all the parameters within the model.

**Parameter**	**Description**
β	Population average contact rate.
γ	Population average recovery rate.
k	Shape parameter that characterises the gamma distributed infection duration.
s_g, s_f, s_r	SNP effects, *i.e.* fractional change in susceptibility, infectivity or recoverability coming from an *A* allele compared to a *B* allele.
Δ_g, Δ_f, Δ_r	Corresponding scaled dominance factors (1 when *A* is completely dominant over *B*).
$\boldsymbol{b}_g$, $\boldsymbol{b}_f$, $\boldsymbol{b}_r$	Vectors of fixed effects for the three traits.
$\mathbf{X}$	Design matrix for fixed effects (*e.g.* this could have value 0/1 for male/female).
$\boldsymbol{a}_g$, $\boldsymbol{a}_f$, $\boldsymbol{a}_r$	Additive genetic contributions to the traits.
$\mathbf{A}$	Relationship or genomic relationship matrix.
$\mathbf{\Omega}$	3x3 covariance matrix for additive genetic contributions. This accounts for potential correlations between traits (*e.g.* genetically more susceptible individuals may also be genetically more infectious).
$\boldsymbol{\varepsilon}_g$, $\boldsymbol{\varepsilon}_f$, $\boldsymbol{\varepsilon}_r$	Residual contributions to the traits (that is individual-based variation over and above that coming from other effects).
$\mathbf{I}$	Identity matrix.
$\mathbf{\Psi}$	3x3 covariance matrix for residual contributions. This accounts for potential correlations between traits (*e.g.* more susceptible individuals may also be more infectious).
c_z	Group effects (accounts for fractional differences in transmission rates in different groups).
σ_c	Standard deviation in group effects.

Table 1. A description of model parameters.

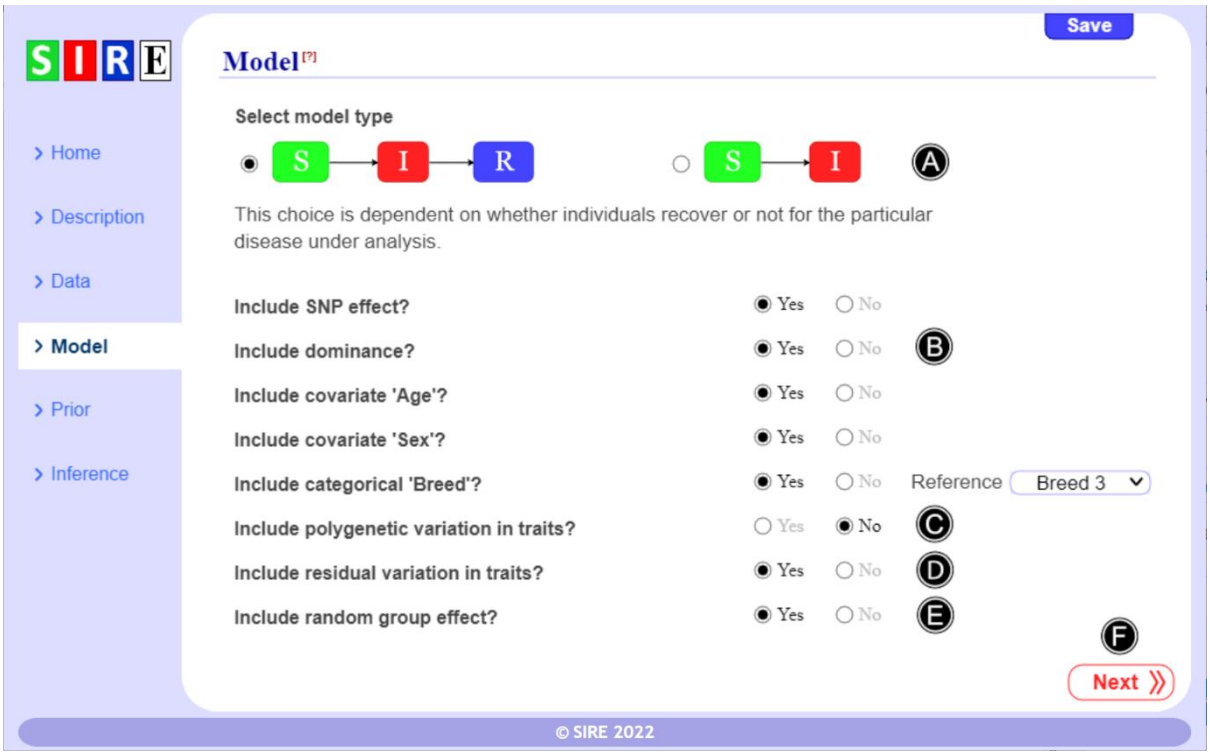

Figure 7 – *Selecting the model.* A: Choose depending on whether recoveries or mortalities occur or not, B: choose which SNP or fixed effects to include (note, for categorical fixed effects the reference needs to be specified), C: include polygenic variation in traits (requires a relationship matrix to be loaded), D: include residual variation in traits, E: include random group effect or not, F: go to next page.

Various features of the model outlined above can be altered:

- Figure 7A determines the compartment model type. For some diseases individuals do not recover (*e.g.* bovine tuberculosis), so a simper SI model is more appropriate than the full SIR.
- Figure 7B allows for SNP (with or without dominance) or fixed effects to easily be turned on or off in the model.
- Figure 7C determines the inclusion or absence of the additive genetic $\boldsymbol{a}$ terms in Eq.(2).
- Figure 7D determines the inclusion or absence of the residual ε terms in Eq.(2).
- Figure 7E determines the inclusion or absence of the c_z group effect term in Eq.(1). In a well-controlled disease challenge experiment, where extraneous factors are largely controlled, it may be appropriate to neglect group effects because their inclusion in the model leads to an unnecessary reduction in parameter precision. On the other hand, for most real-world field data, environmental variation across different locations would doubtless lead to substantial variation in transmission rate, and so inclusion of c_z becomes a necessity.

Click “Next” (Fig.7F) after completing model specification.

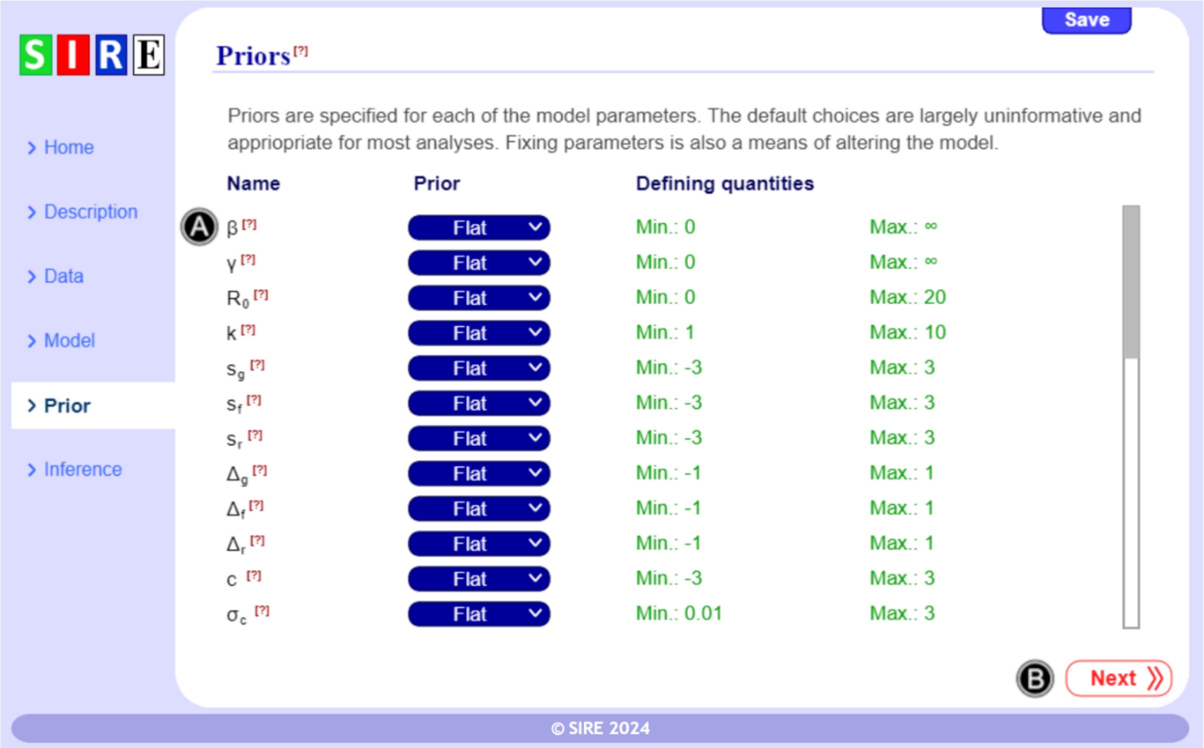

Figure 8 – *Specification of the prior.* A: A list of all model parameters along with prior specifications (note parameters can also be fixed by selecting “Fix” from the drop-down menu). This example shows default values used in SIRE 2.0, B: go to the next page.

2.4 The priors

Priors are specified for each of the model parameters (see Table 1 for reference). The default choices are largely uninformative but do place upper and lower bounds on many of the key parameters (this stops them straying into biologically unrealistic regimes during inference, see appendix C in [1] for further details). Bounding parameters in this way is especially important when considering relatively uninformative data scenarios, in which unbounded flat priors would lead to improper posterior probability distributions.

SIRE 2.0 supports the following prior specifications: flat, which relates to a uniform probability distribution across a range, and the gamma, normal, log-normal and beta distributions, as well as the possibility to fix parameters to specific known values. Additionally, the prior can be chosen to return to its default setting.

It should be noted that the default settings in SIRE 2.0 are generally suitable for most scenarios and changing them is usually only appropriate if specific knowledge on parameter values actually exists. However, fixing parameters is also a means of altering the model. For example, setting shape parameter k=1 results in the model assuming exponentially distributed infection duration (*i.e.* Markovian).

Click “Next” (Fig. 8B) to complete the data and model section and move onto inference.

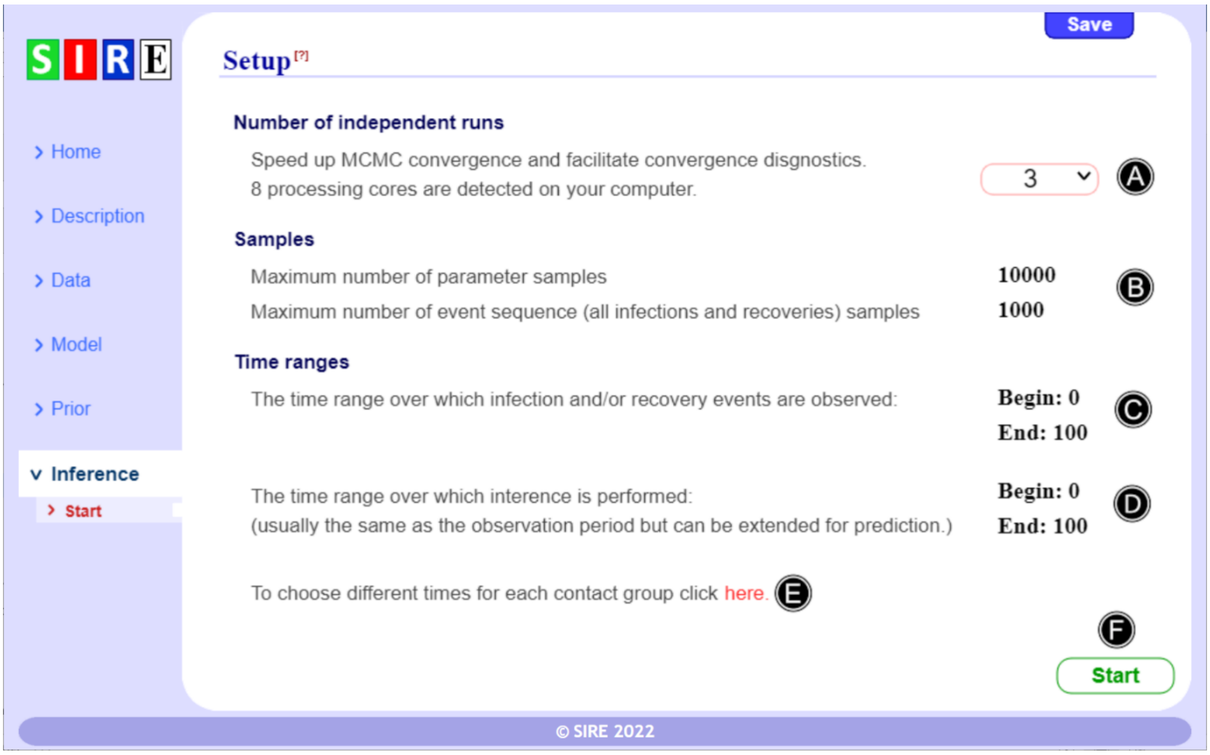

Figure 9 – *Initialising inference.* A: Number of MCMC runs, B: limits the number of parameter and event sequence samples, C: inference time range, D: observation time range, E: select different times for each group, F: start inference.

3 Outputs

Based on the data entered in section 2 it is generally not possible to identify model parameters with perfect precision (or infection and recovery times for that matter, unless they are specified in the data). Rather there exists a distribution in these quantities known as the "posterior", which expresses both a best guess for parameters (*i.e.* posterior means) along with ranges in parameter values consistent with the data (*i.e.* credible intervals). SIRE 2.0 achieves Bayesian inference by means of drawing samples from this posterior distribution using a widely applied technique known as Markov chain Monte Carlo (MCMC) [2]. Unlike other statistical techniques (such as maximum likelihood) MCMC does not simply output a final answer. Rather it successively generates samples which progressively improve the accuracy of the posterior estimate until sufficient accuracy is achieved (how long this process takes is discussed in section 3.6).

3.1 Starting inference

Several options must be selected before inference can begin:

- The number of MCMC runs to be executed is selected using the drop-down menu at Fig. 9A. Each run exists on the computer as a separate process, and since most modern computers contain multiple CPU cores, computationally efficiency can be substantially improved by

selecting more than one run[5]. Additionally, executing multiple runs allows for MCMC diagnostics to verify convergence (see section 3.6). A suitable choice is 3, which is used as the default value.

- As SIRE 2.0 runs it collects parameters samples for both the model parameters and infection/recovery events for all the individuals in the population. Setting the values in Fig. 9B higher allows for smoother output plots to be made, but can potential lead to insufficient computational memory. The default values represent a sensible trade-off between the two.
- Figure 9C shows the time range over which inference is performed. This must include the times at which data is actually collected, but may also be extended forward in time to allow for future model prediction. It is important to note here that at the "Begin" time SIRE 2.0 assumes that all individuals are susceptible. Consequently in scenarios in which the initiation time of epidemics is unknown, this "Begin" time must be set significantly prior to the time at which data is collected.
- In cases in which infection and/or recovery data is available, it is necessary to identify the time range over which these observation are made (Fig. 9D). This allows for the possibility of time censoring whereby only the beginning or end of the epidemics are actually observed.
- Clicking the option on Fig. 9E allows the user to specify time ranges for each of the contact groups separately (if necessary).

Bayesian inference can now begin by clicking on the "Start" button (Fig. 9F).

3.2 Trace plots

As seen in Fig. 10A, the first screen after inference is started shows trace plots for model parameters. As mentioned previously, MCMC works by successively drawing parameter samples (represented by the x-axis) from the posterior. Ideally these samples are randomly distributed, but in reality they are correlated (which manifests itself by structure within these plots). The example in Fig. 10A is one in which mixing is good, because the curves exhibit substantial variation up and down about the posterior mean. Under different circumstances, however, MCMC runs can exhibit poor mixing, resulting in SIRE 2.0 taking much longer to provide results adequately representative of the posterior. The examples in section 4 (which all consist of analysing 1000-4000 individuals under different data scenarios) take from a few seconds to a few minutes to adequately mix. Measures for assessing how long inference should be performed are discussed in section 3.6.

Different parameters are selected in the following way: The drop-down menu (Fig. 10B) classifies different types of variable ("Epi." gives epidemiological parameters, "SNP" gives parameters related to SNPs, "Fix. Eff." gives any fixed effects, "Covar." gives the covariance matrices, "Gr. Eff." gives the group effects, and "Misc." gives any other quantities, such as likelihoods and prior probabilities) and the options in Fig. 10C allows for choosing parameters within each type.

Note, when the number of parameter samples exceeds the value in Fig. 9B, samples are thinned by a factor of two and subsequently gathered at half the rate (this is implemented to ensure that computational memory is not exhausted). The vertical dashed red line in Fig. 10A represents the so-

---

[5] Executing more runs than the number of CPU cores can lead to a substantial slowing down of the SIRE interface. This is not expected to provide any further improvement in computational efficiency, and so is not recommended.

called burn-in period (before which samples are discarded). To maximise efficiency this is dynamically shifted as more and more posterior samples are generated.

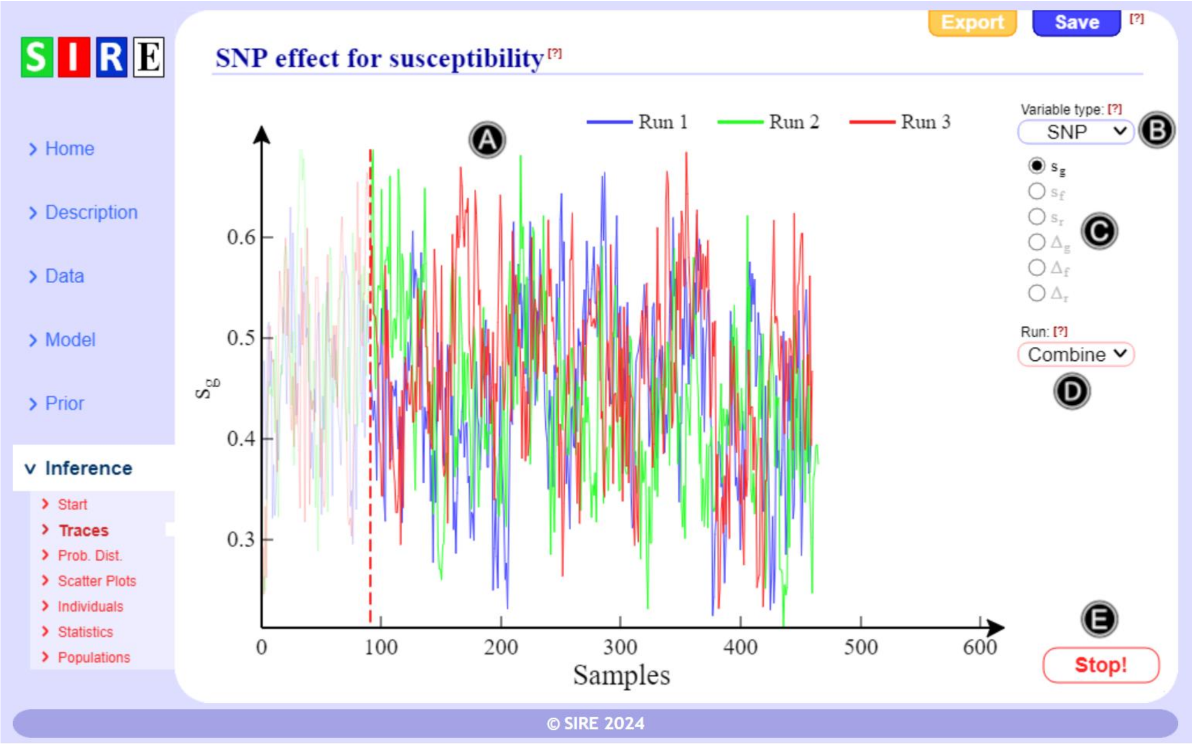

Figure 10 – *Trace plot.* A: Posterior samples for selected parameter, B: variable type selection, C: variable selection, D: which runs should be displayed, E: stop gathering samples.

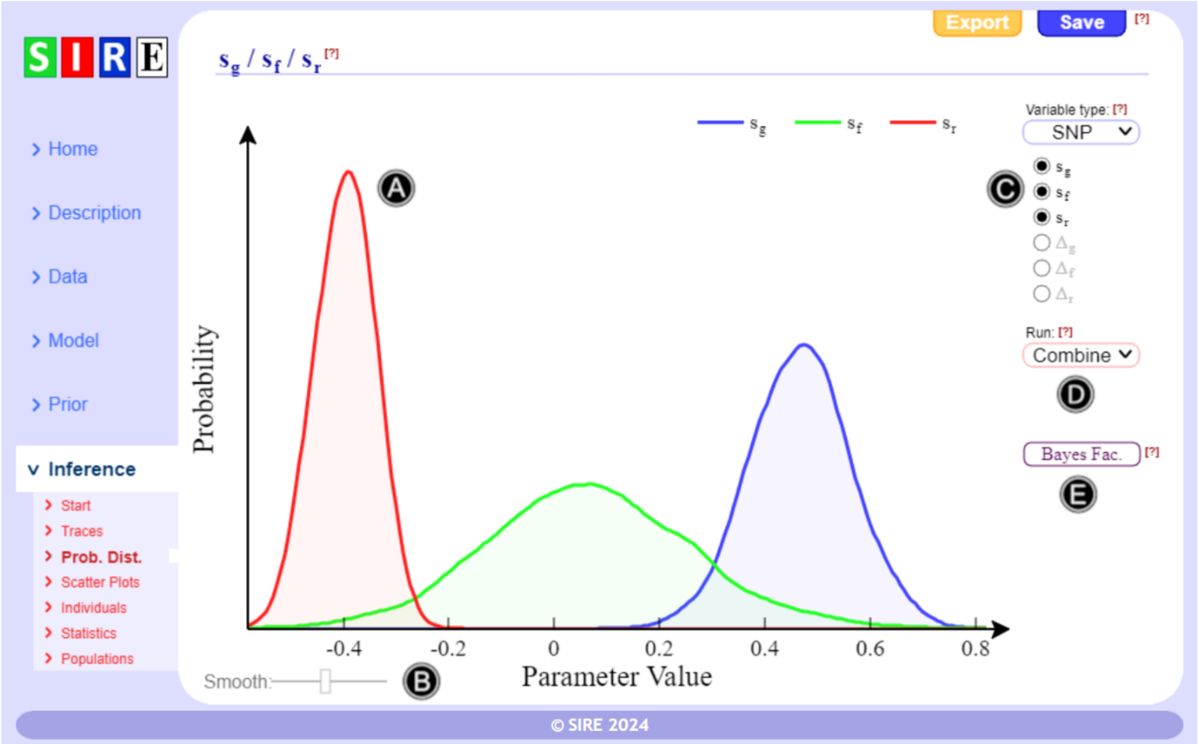

Figure 11 – *Distributions.* A: Probability distribution(s) (use ctrl key to select multiple parameters), B: KDE smoothing parameter, C: parameter selection, D: show results from individual MCMC runs or combined together, E: calculate the Bayes' factor.

3.3 Probability distributions

The raw posterior samples from the previous section can be converted into posterior probability distributions, as shown in Fig. 11A. These are generated using a technique known as kernel density estimation (KDE) [3]. KDE makes use of a smoothing parameter which can be adjusted by means of the slider at Fig. 11B. This particular example simultaneously shows distributions for three model parameters, which can be achieved by holding down the control key and sequentially selecting the relevant parameters at Fig. 11C.

A Bayes factor (BF) is the ratio in the likelihood of one particular hypothesis to the likelihood of another [4]. The BF comparing the full model to one in which a particular parameter is fixed (usually to zero) can be calculated using the button at Fig. 11E. This is one way to determine statistically significant SNP and fixed effects affecting the three traits. A BF between 3 and 10 represents moderate evidence for one hypothesis over another and exceeding 10 is considered strong evidence.

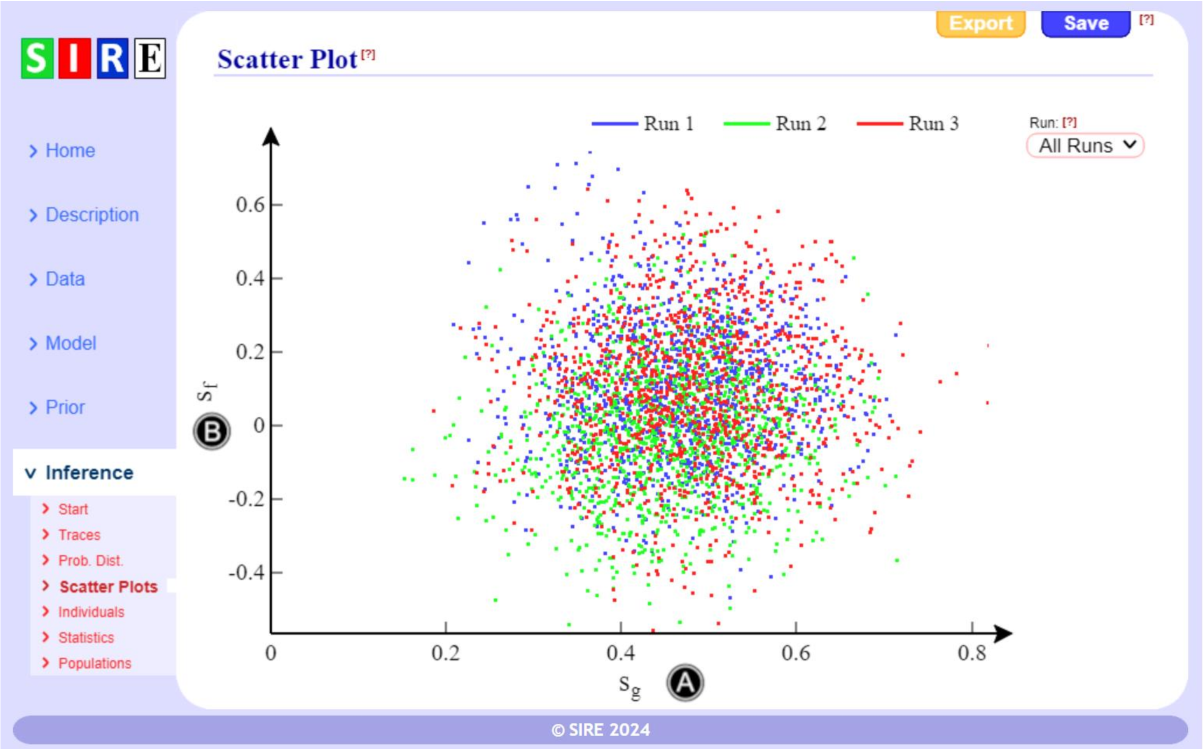

Figure 12 – *Scatter plot.* A: Select x variable, B: select y variable.

3.4 Scatter plot

Scatter plots, as illustrated in Fig. 12, enable the user to display the posterior samples of one variable against another. This is achieved by means of clicking the x-axis (Fig. 12A) selecting the relevant variable and then doing the same for the y-axis (Fig. 12B). This particular example exhibits little correlation between the variables a_g and a_f, but in other cases parameters can be highly correlated. Scatter plots are a useful tool to investigate confounding between different model parameters.

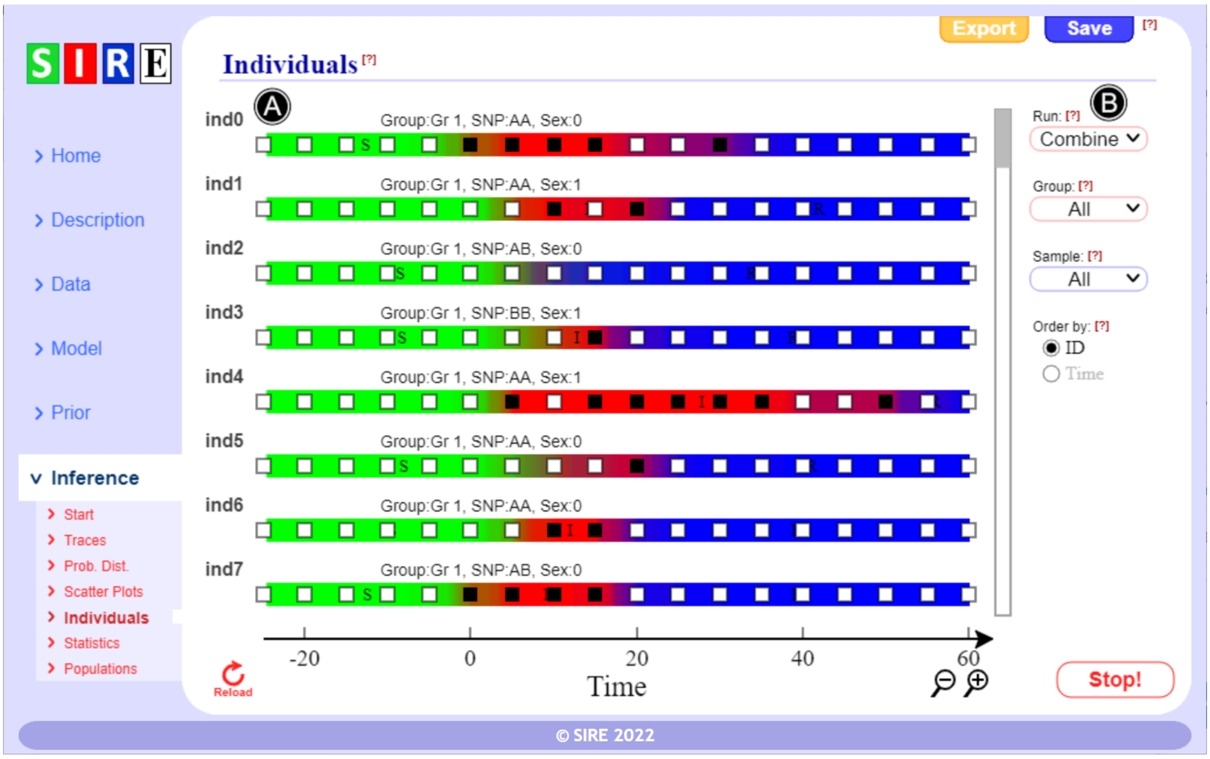

Figure 13 – *Individual timeline plots.* A: Shows posterior distributions for individual timelines (taken from EX.7 which used disease diagnostic test results), B: various filters which can be applied.

3.5 Individual timelines

The view in Fig. 13A shows posterior distributions for individual timelines. Here the colours indicate the posterior probabilities for the disease status of individuals as a function of time. Green indicates that an individual is susceptible, red indicates infectious and blue indicates recovered. Gradations in colour between these extremes represent posterior uncertainty. Overlaid are the actual data (disease diagnostic test results in this particular example, where black/white squares indicate a positive/negative outcome).

Various filters in Fig. 13B can be applied, such as only displaying individuals within a given contact group, a particular run or a given sample. Also the ordering can be based on individual ID or on the time at which they are first observed.

3.6 Statistics

SIRE 2.0 summarises the posterior probability distributions (specifically the means and 95% credible intervals) for all the model parameters, as shown in Fig. 14. The credible intervals are of particular importance, because they can be used to establish if a particular SNP or fixed effect is statistically significant or not. For example, we note that the credible interval for a_g in Fig. 14 goes from 0.295 to 0.668. This represents strong evidence that the genotype at the SNP truly does affect the susceptibility of individuals, because this range does not contain zero. On the other hand the same cannot be said for a_f, and so here nothing can definitively be concluded regarding SNP-based infectivity variation.

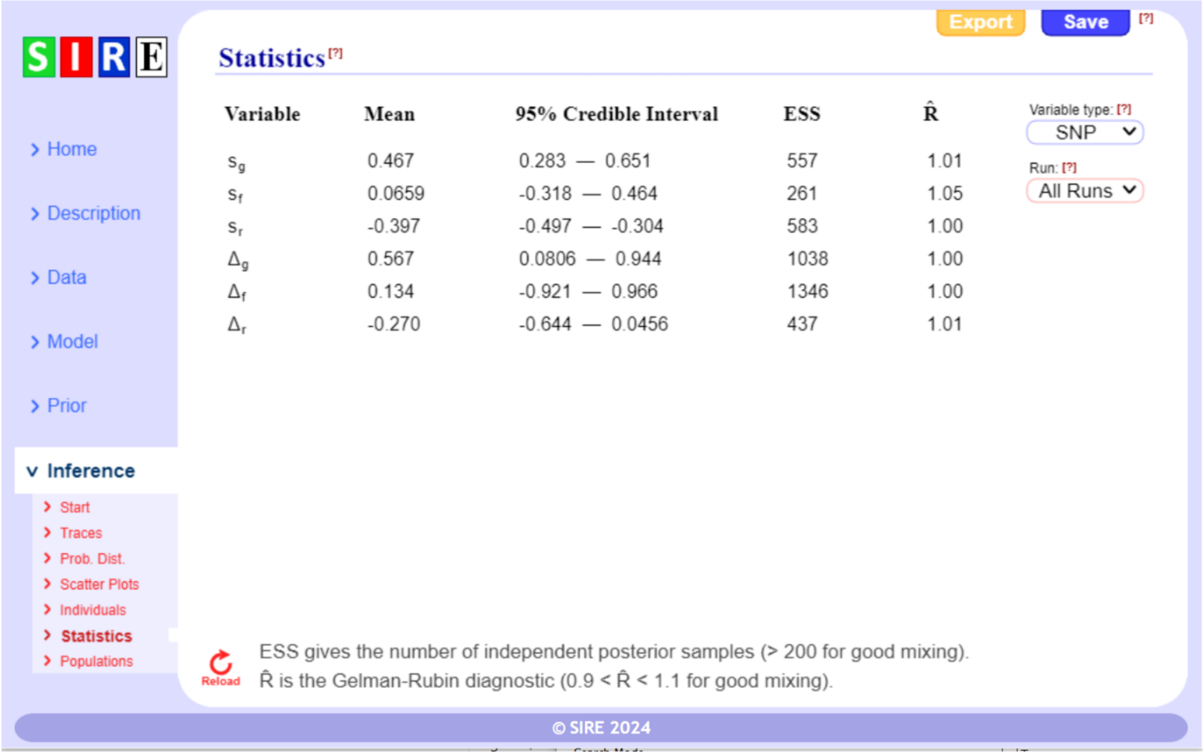

Figure 14 –*Statistics.* A summary of posterior parameter estimates along with MCMC diagnostics.

Two measures are used to test for MCMC convergence:

- **Effective sample size (ESS)** – This estimates the number of truly independent posterior samples [2] (*i.e.* it accounts for correlations shown in the trace plots in section 3.2). Provided the ESS is greater than around 200 then posterior estimates can be reliably trusted. This is the case in Fig. 14 so SIRE 2.0 can be stopped. Note, the ESS is not always guaranteed to monotonically increase (in fact if it is less than 100 it often fluctuates wildly). However, these fluctuation should dampen with increasing sample size.
- **The Gelman-Rubin statistic $\hat{R}$** – This checks that distributions from independent runs converge on the same posterior probability distribution [5]. Values between around 0.9 and 1.1 are considered to be indicative of convergence. If $\hat{R}$ fails to approach 1, even after a large number of iterations, this may be an indication of multimodality in the posterior distribution. This describes a scenario in which different MCMC chains become stuck in different local minima. This possibility can occur when there is only weak individual-based data, as is evident in [1] when SIRE 2.0 was applied to final disease status data. Under these circumstances the results from SIRE 2.0 cannot be trusted. Note this statistic relies on comparing independent MCMC runs, and so is not applicable when only a single run is being executed.

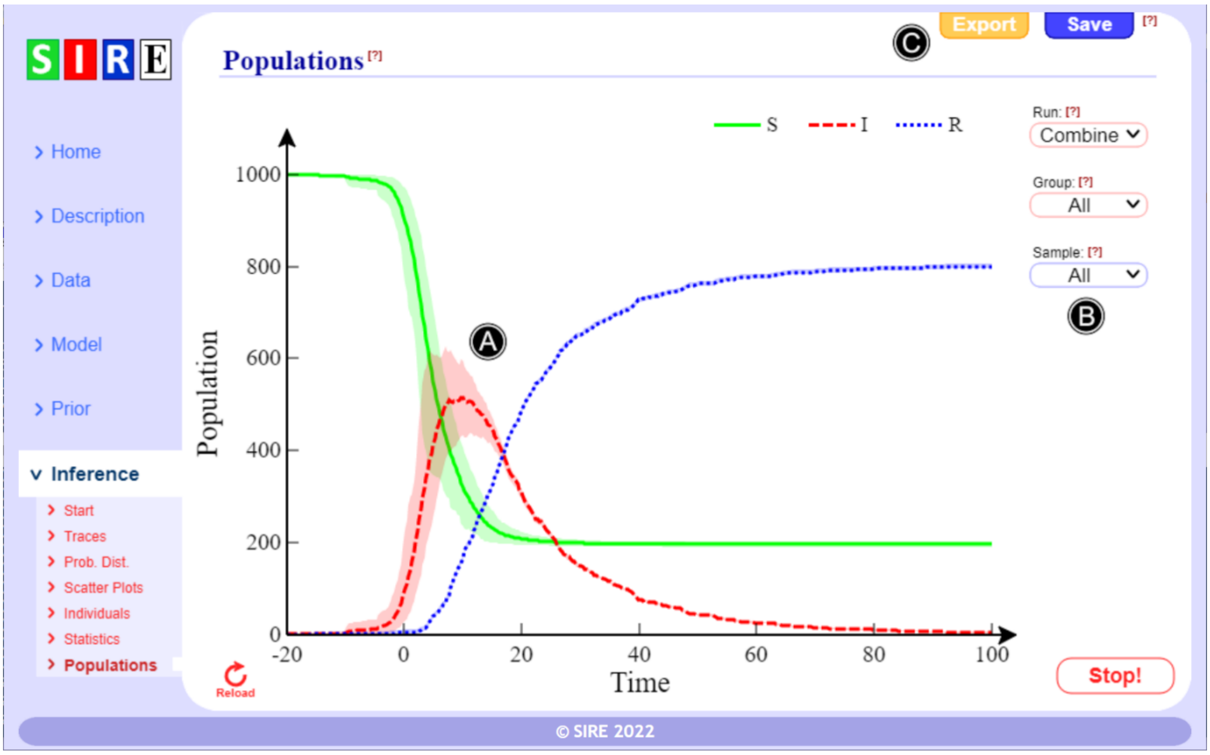

Figure 15 –*Population plots.* A: The dynamic variation in the populations of susceptible, infected and recovered individuals (taken from EX.3), B: filters, C: exporting and saving.

3.7 Population plots

The number of susceptible, infected and recovered individuals can be plotted as a function of time, as illustrated in Fig. 15A. Here the lines represent posterior means and the shaded areas give 95% credible intervals. The results can be filtered by MCMC run, contact group or sample number (Fig. 15B).

3.8 Exporting

Exporting outputs can be achieved by clicking on the “Export” button on the top right hand corner (*e.g.* see Fig. 15C). A number of exporting possibilities exist:

- **Graph (.png)** – This outputs graphs (such as trace, scatter and population plots) as images.
- **Graph (.txt)** – This outputs the raw data so they can be plotted using other software.
- **Parameters** – This outputs posterior parameter samples in text format (for analysis in other software).
- **Events** – This outputs posterior samples giving the raw infection and recovery times of individuals in text format (for subsequent analysis in other software).
- **EBVs** – Generates a table of estimated breeding values (EBVs). Outputs means and standard deviations for $\boldsymbol{a}=(\boldsymbol{a}_g, \boldsymbol{a}_f, \boldsymbol{a}_r)$ from Eq.(2).

3.9 Loading and saving

SIRE 2.0 permits users to load and save analyses in a special “.sire” format (see Fig. 15C for saving and and Fig. 1B for loading). This is useful because it conveniently allows description, data and analysis to all be contained in a single file for future reference. Also those publishing results using SIRE 2.0 can include the “.sire” file in the supplementary material such that analysis can

transparently be reproduced by readers of the paper. When saving, two options are available: "With results" includes the posterior samples along with the model and data (so that inference does not need to be run again when the file is loaded), and "W/o results" which does not store the posterior samples (leading to a much smaller file size which can, for example, be emailed).

4 Examples

This section describes various examples chosen to illustrate potential applications of SIRE 2.0 (see Fig. 1E).

The examples are split according to whether they used an SIR or SI model, and whether they contain a polygenic contribution. For non-polygenic models, 1000 individual were assumed split equally into 50 contact groups, each containing 20 individuals. For polygenic models, 100 sires were assumed to be randomly mated with 20 dams to generate 2000 individuals in the next generation. These progeny were then randomly split equally into 200 contact groups, each containing 10 individuals.

Simulated data were generated by means of a modified Doob-Gillspie algorithm in which each contact group is assumed to initially contain a single infected individual with others in the susceptible compartment. Data tables derived from these simulations are located in the subdirectory "Datasets" of the downloaded SIRE 2.0 folder.

Different disease scenarios (broadly these are classified as DS1: infection/recovery times known precisely, DS2: only recovery times known, DS3: only infection times known, DS4: periodic disease state data measurements and DS5: time censoring) are considered.

4.1 SIR model

Without polygenic contribution

- **EX.1: Known infection and recovery times –** This example assumes that the infection and recovery times for all individuals are known (DS 1). Inspecting the posterior distributions for model parameters we find that overall they accurately represent the true parameter values used to simulate the data (see above). Data was taken from "Dataset 1.txt" in the "Datasets" folder.
- **EX.2: Staggered contact group timings –** Here the times at which different contact groups have epidemics are staggered. Again infection and recovery times for all individuals are assumed known (DS 1). Data was taken from "Dataset 2.txt".
- **EX.3: Known recovery times** – This example assumes that only the recovery times for all individuals are known (DS 2). Data was taken from "Dataset 3.txt".
- **EX.4: Disease transmission experiment** – This assumes that not only are the recovery times for all individuals known (DS 2), but also the initial disease status at t=0 is defined by the experiment. Data was taken from "Dataset 3.txt".
- **EX.5: Known infection times** – This example assumes that only the infection times for all individuals are known (DS 3). Data was taken from "Dataset 3.txt".
- **EX.6: Periodic disease status checks –** This example assumes that the disease status of individuals are measured periodically (DS 4). Data was taken from "Dataset 4.txt".

- **EX.7: Disease diagnostic test results –** Here periodic disease diagnostic tests are performed on individuals (DS 4). The test is assumed to be sensitive to the *I* state. Data was taken from "Dataset 5.txt".
- **EX.8: Disease diagnostic test results II –** Here two sets of diagnostic tests are made on individuals. The first is sensitive to the *I* state and the second is sensitive to both *I* and *R* states. Data was taken from "Dataset 5.txt".
- **EX.9: Time censoring end of epidemics –** This illustrates data censoring (DS5), whereby the infection and recovery times for individuals are only observed up to a time before the end of epidemics. Data was taken from "Dataset 6.txt".
- **EX.10: Time censoring beginning of epidemics –** This illustrates data censoring (DS5), whereby the infection and recovery times for individuals are only observed after epidemics have already started (*i.e.* missing the beginning). Data was taken from "Dataset 7.txt".

With polygenic contribution

- **EX.11: Known infection and recovery times –** This example assumes that the infection and recovery times for all individuals are known (DS 1). Data was taken from "Dataset 14.txt" and "Dataset 15.txt".
- **EX.12: Periodic disease status measurements –** This example assumes that the disease status of individuals are measured periodically (DS 4). Data was taken from "Dataset 16.txt", "Dataset 17.txt" and "Dataset 18.txt".
- **EX.13: Two sets of disease diagnostic test results –** Here two sets of diagnostic tests are made on individuals. The first is sensitive to the *I* state and the second is sensitive to both *I* and *R* states. Data was taken from "Dataset 19.txt", "Dataset 20.txt" and "Dataset 21.txt".

4.2 SI model

This is a simplified model for diseases in which individuals do not recover/die. Again, different illustrative data scenarios are considered:

Without polygenic contribution

- **EX.14: Known infection and recovery times –** This example assumes that the infection and recovery times for all individuals are known (DS 1). Data was taken from "Dataset 8.txt".
- **EX.15: Staggered contact group timings –** Here the times at which different contact groups have epidemics are staggered. Again infection and recovery times for all individuals are assumed known (DS 1). Data was taken from "Dataset 9.txt".
- **EX.16: Periodic disease status checks –** This example assumes that the disease status of individuals are measured periodically (DS 4). Data was taken from "Dataset 10.txt".
- **EX.17: Disease diagnostic test results –** Here periodic disease diagnostic tests are performed on individuals (DS 4). Data was taken from "Dataset 11.txt".
- **EX.18: Time censoring end of epidemics –** This illustrates data censoring (DS5), whereby the infection and recovery times for individuals are only observed up to a time before the end of epidemics. Data was taken from "Dataset 12.txt".
- **EX.19: Time censoring beginning of epidemics –** This illustrates data censoring (DS5), whereby the infection and recovery times for individuals are only observed after epidemics have already started (*i.e.* missing the beginning). Data was taken from "Dataset 13.txt".

With polygenic contribution

- **EX.20: Known infection and recovery times –** This example assumes that the infection times for all individuals are known (DS 1). Data was taken from "Dataset 22.txt" and "Dataset 23.txt".
- **EX.21: Periodic disease status measurements –** This example assumes that the disease status of individuals are measured periodically (DS 4). Data was taken from "Dataset 22.txt" and "Dataset 23.txt".
- **EX.22: Disease diagnostic test results –** Here periodic diagnostic tests are made on individuals. Data was taken from "Dataset 24.txt", "Dataset 25.txt" and "Dataset 26.txt".

5 Code

The code for SIRE 2.0 is split into two parts:

- **The interface** – This is written in JavaScript and runs on the desktop by means of software package NW.js (see nwjs.io). For those interested, the code consists of the "index.html" file in the main directory[6] and the JavaScript files in the "js" directory.
- **The core code** – Performs the MCMC Bayesian analysis when SIRE 2.0 is executed. This is written in highly efficient C++ code which can be found in the "Execute" directory (it consists of "sire.cc" along with numerous header files).

6 License and warranty

SIRE 2.0 is free software under the terms of the GNU General Public License version 3 www.gnu.org/licenses/gpl-3.0.en.html. This allows users to redistribute and/or modify SIRE 2.0. The program is distributed in the hope that it will be useful, but WITHOUT ANY WARRANTY.

7 Citing SIRE

We kindly request that those who do use SIRE analysis in their publications cite this tool.

SIRE 1.0 is currently published:

Pooley CM, Bishop SC, Doeschl-Wilson AB, Marion G. *Estimating genetic and non-genetic effects for host susceptibility, infectivity and recoverability using temporal epidemic data*." *PLOS Computational Biology* 16.12 (2020): e1008447.

A paper for SIRE 2.0 is in preparation.

8 Acknowledgments

SIRE 2.0 makes use of two other pieces of software and we would like to acknowledge their contribution. Firstly, NW.js (from the website nwjs.io/) was used to build the interface. Secondly, tinyXML (from the website www.grinninglizard.com/tinyxml/) was used by the C++ code to parse the

[6] On the Macintosh platform this is located in the "SIRE.app/Contents/Resources/app.nw/"folder.

XML file which provides initialisation information. Both these software are excellent and highly recommended to others.

References

[1] Pooley C.M., Bishop S.C., Doeschl-Wilson A.B., Marion G., Estimating genetic and non-genetic effects for host susceptibility, infectivity and recoverability using temporal epidemic data, bioRxiv. (2019) 618363.

[2] Geyer C.J., Practical Markov Chain Monte Carlo, Statist. Sci. 7 (1992) 473-483.

[3] Parzen E., On estimation of a probability density function and mode, The annals of mathematical statistics. 33 (1962) 1065-1076.

[4] Kass R.E., Raftery A.E., Bayes factors, Journal of the american statistical association. 90 (1995) 773-795.

[5] Gelman A., Rubin D.B., Inference from iterative simulation using multiple sequences, Statistical science. 7 (1992) 457-472.
