# Supplementary material for: SIRE 2.0: a novel method for estimating polygenic host effects underlying infectious disease transmission, and analytical expressions for prediction accuracies
Source: Genet Sel Evol. 2025 Apr 1;57:17. doi: 10.1186/s12711-025-00956-4 (PMC11963337; doi:10.1186/s12711-025-00956-4)
Supplement: Supplementary file 6 — Additional file 6. Analytic expressions for prediction accuracies. Shows the derivation of mathematical expressions for prediction accuracy from the posterior. [file 12711_2025_956_MOESM6_ESM.pdf]

Analytic expressions for prediction accuracies

The aim here is to obtain analytical expressions for prediction accuracies (PAs) for additive genetic contributions to susceptibility, infectivity, and recoverability. These accuracies measure correlations between the posterior means for $\boldsymbol{a}_g$, $\boldsymbol{a}_f$, and $\boldsymbol{a}_r$ (based on the data) and their true (usually unknown) values.

Here we consider a scenario that consists of Z contact groups each containing a set of N individuals with genetic relationship specified by a pedigree or genomic relationship matrix $\mathbf{A}$. Epidemics are instigated by means of a single infected index case. For tractability this analysis assumes that epidemic dynamics are governed by a simple SIR (or SI) model and that the infection and recovery times of all individuals are known. Furthermore, traits are assumed to be uncorrelated and the diagonals of the matrices $\mathbf{\Omega}$ and $\mathbf{\Psi}$, as well as values for the transmission rate β and recovery rate γ, are taken to be exactly known (equivalent to assuming that the number of contact groups Z is large). Lastly, fixed and group effects are ignored.

Because the likelihood in Eq.(4) naturally divides into a part related to the infection process and a part related to the recovery process, so analysis can be performed separately for each. First we look at PAs for susceptibility/infectivity and later the PA for recoverability is derived.

PAs for susceptibility/infectivity

By Bayes' theorem

$$\pi(\theta \mid \xi) \propto \pi(\xi \mid \theta)\pi(\theta) \tag{A1}$$

Substituting the part corresponding to the infection process in the likelihood in Eq.(4) and the prior in Eq.(A2) in Additional file 2 leads to the posterior probability

$$\pi(\theta \mid \xi) \propto \prod_z \left[\left(\prod_{j \in z} \lambda_j \right) \left(\prod_{e \in z} e^{-\Lambda_z(t_e)\times(t_e - t_{e-1})} \right) \right] e^{-\frac{1}{2}\sum_{t,m,n,u} a_{t,m} \mathrm{A}^{-1}_{mn} \Omega^{-1}_{tu} a_{u,m} - \frac{1}{2}\sum_{t,m,u} \varepsilon_{t,m} \Psi^{-1}_{tu} \varepsilon_{u,m}}, \tag{A2}$$

where z goes over contact groups, j goes over infected individuals (the notation $j \in z$ denotes the fact that j only goes over individuals within group z and the symbol "∈" is subsequently used in a corresponding fashion), and e goes over infection events that occur at times t_e (not to be confused with Euler's number e also used in this expression). The quantities t and u sum over trait types (*i.e.* g, f and r correspond, respectively, to susceptibility, infectivity and recoverability) and m and n sum over all individuals in the system.

Substituting in the explicit expression for the force of infection in Eq.(1) and using Eq.(2) gives

$$\pi(\theta \mid \xi) \propto \beta^{N_I} e^{-\beta J} \prod_z \left[\prod_{j \in z} \left(e^{a_{g,j} + \varepsilon_{g,j}} \sum_{i \in j} e^{a_{f,i} + \varepsilon_{f,i}} \right) \right] e^{-\frac{1}{2}\sum_{t,m,n,u} a_{t,m} \mathrm{A}^{-1}_{mn} \Omega^{-1}_{tu} a_{u,m} - \frac{1}{2}\sum_{t,m,u} \varepsilon_{t,m} \Psi^{-1}_{tu} \varepsilon_{u,m}}, \tag{A3}$$

where i goes over all infected individuals in group z immediately prior to the time when individual j gets infected and N_I is the total number of infected individuals over all groups. The quantity J in Eq.(A3) is given by

$$J = \sum_z \left(\sum_{e \in z} \left[\left(\sum_{s \in e} e^{a_{g,s}+\varepsilon_{g,s}} \right) \left(\sum_{i \in e} e^{a_{f,i}+\varepsilon_{f,i}} \right) \left(t_e - t_{e-1} \right) \right] \right), \tag{A4}$$

where the sums s and i go over the susceptible and infected populations in z immediately prior to event e.

The log of the posterior probability in Eq.(A3) is given by (up to a constant term)

$$\begin{aligned} l = \log\left[\pi(\theta \mid \xi)\right] = N_I \log(\beta) - \beta J + \sum_z \left(\sum_{j \in z} \left[a_{g,j} + \varepsilon_{g,j} + \log\left(\sum_{i \in j} e^{a_{f,i}+\varepsilon_{f,i}} \right) \right] \right) \\ -\tfrac{1}{2} \sum_{t,m,n,u} a_{t,m} \mathrm{A}_{mn}^{-1} \Omega_{tu}^{-1} a_{u,n} - \tfrac{1}{2} \sum_{t,m,u} \varepsilon_{t,m} \Psi_{tu}^{-1} \varepsilon_{u,m}. \end{aligned} \tag{A5}$$

.

The partial derivatives of this are

$$\begin{aligned} \frac{\partial l}{\partial a_{g,m}} &= 1 - e^{a_{g,m}+\varepsilon_{g,m}} \beta \sum_{e \in m_S} \left[\left(\sum_{i \in e} e^{a_{f,i}+\varepsilon_{f,i}} \right) \left(t_e - t_{e-1} \right) \right] - \sum_{n,u} \mathrm{A}_{mn}^{-1} \Omega_{gu}^{-1} a_{u,n}, \\ \frac{\partial l}{\partial a_{f,m}} &= e^{a_{f,m}+\varepsilon_{f,m}} \left[\sum_{e \in m_I} \left(\frac{1}{\sum_{i \in e} e^{a_{f,i}+\varepsilon_{f,i}}} \right) - \beta \sum_{e \in m_I} \left[\left(\sum_{s \in e} e^{a_{g,s}+\varepsilon_{g,s}} \right) \left(t_e - t_{e-1} \right) \right] \right] \\ &\quad - \sum_{n,u} \mathrm{A}_{mn}^{-1} \Omega_{fu}^{-1} a_{u,n}, \end{aligned} \tag{A6}$$

where m_S represents those infection events that occur during the time period in which individual m is susceptible and within the contact group in which m resides. Correspondingly, m_I represents only those events during the time period in which individual m is infected.

This analytical treatment estimates the mean posterior values for the additive genetic effects for susceptibility $\boldsymbol{\mu_g}$ and infectivity $\boldsymbol{\mu_f}$ by the values that $\boldsymbol{a_g}$ and $\boldsymbol{a_f}$ take in order to maximise the posterior probability distribution in Eq.(A5). This maximum is calculated by setting the derivatives in Eq.(A6) to zero and solving. In the general case this set of equations is intractable and to make further progress we take the limit in which $\boldsymbol{a_g}$, $\boldsymbol{a_f}$, $\boldsymbol{\varepsilon_g}$, $\boldsymbol{\varepsilon_f}$ are all assumed to be small. Taylor expanding Eq.(A6) in these quantities we find that, to first order, the terms involving the relationship matrix dominate. Consequently

$$\begin{aligned} &1 - \beta \sum_{e \in m_S} \left[I_e \left(t_e - t_{e-1} \right) \right] - \sum_{n,u} \mathrm{A}_{mn}^{-1} \Omega_{gu}^{-1} \mu_{u,n} = 0, \\ &\sum_{e \in m_I} \left(\frac{1}{I_e} \right) - \beta \sum_{e \in m_I} \left[S_e \left(t_e - t_{e-1} \right) \right] - \sum_{n,u} \mathrm{A}_{mn}^{-1} \Omega_{fu}^{-1} \mu_{u,n} = 0, \end{aligned} \tag{A7}$$

where S_e and I_e represent the total susceptible and infected populations immediately preceding event e. Remembering that $\boldsymbol{\Omega}$ is a diagonal matrix (*i.e.* correlations are ignored), this can be rearranged to

$$\mu_{g,n} = \Omega_{gg} \sum_m \left[\mathrm{A}_{nm} \left(1 - \beta \sum_{e \in m_S} \left[I_e \left(t_e - t_{e-1} \right) \right] \right) \right] \tag{A8}$$

and

$$\mu_{f,n} = \Omega_{ff} \sum_m \left[\mathrm{A}_{nm} \left(\sum_{e \in m_I} \left(\frac{1}{I_e} \right) - \beta \sum_{e \in m_I} \left[S_e \left(t_e - t_{e-1} \right) \right] \right) \right]. \tag{A9}$$

These two expressions are estimated in the following two subsections:

Estimating $\mu_{g,n}$

Suppose we simulate from the model using a given set of model parameters θ. The various quantities in Eq.(A8) can be estimated. First we start with

$$\sum_{e \in m_S} \left[I_e \left(t_e - t_{e-1} \right) \right], \tag{A10}$$

where the sum goes over events in which individual m is susceptible. For brevity this quantity is denoted by κ_m. The values of κ_m vary drastically between individuals because some become infected earlier on within the epidemic and some later. Furthermore, the order in which individuals becomes infected is essentially random, so to simplify matters we replace Eq.(1) with its mean field approximation

$$\lambda_n = \beta e^{a_{g,n} + \varepsilon_{g,n}} \overline{e^{a_f + \varepsilon_f}}^z I, \tag{A11}$$

where I represents the number of currently infected individuals and the bar with superscript z indicates that the quantity is averaged over all individuals within group z.

Focusing on individual m, the distribution in the likelihood from Eq.(A3) is given by

$$\pi(\kappa_m \mid \theta, \xi_{-m}) \propto e^{-\beta e^{a_{g,m} + \varepsilon_{g,m}} \overline{e^{a_f + \varepsilon_f}}^z \kappa_m}. \tag{A12}$$

This implies that κ_m is, in fact, exponentially distributed, a result which can be expressed as

$$\kappa_m = \frac{u_m}{\beta e^{a_{g,m} + \varepsilon_{g,m}} \overline{e^{a_f + \varepsilon_f}}^z}, \tag{A13}$$

where u_m is an exponentially distributed random variable with unit mean. An intuitive reason for this expression is that if an individual is more susceptible than the population as a whole (*i.e.* $a_{g,m}+\varepsilon_{g,m}$ is positive) then on average it will tend to get infected earlier on within the epidemic (subject to large stochastic fluctuations coming from u_m) and hence κ_m is expected to typically be smaller. Similarly, if the contact group contains many highly infectious individuals $\overline{e^{a_f + \varepsilon_f}}^z$ will be significantly larger than one meaning that epidemics proceed more quickly, again reducing κ_m.

Substitution of Eq.(A13) into Eq.(A8) leads to (assuming effect sizes are small such that the exponential terms can be Taylor expanded)

$$\mu_{g,n} = \Omega_{gg} \sum_m \mathrm{A}_{nm} \left(1 - \left[1 - a_{g,m} - \varepsilon_{g,m} - \overline{a_f}^z \right] u_m \right). \tag{A14}$$

For future manipulation it is of use to rearrange this as

$$\mu_{g,n} = \Omega_{gg} \sum_z \left[\sum_{j \in z} \mathrm{A}_{nj} \left(1 - \left[1 - a_{g,j} - \varepsilon_{g,j} - \tfrac{1}{N} \sum_{q \in z} \left(a_{f,q} + \varepsilon_{f,q} \right) \right] u_j \right) \right], \tag{A15}$$

where z sums over contact groups and j and q sum over all individuals within group z.

Estimating $\mu_{f,n}$

Equation (A9) can be rewritten as

$$\mu_{f,n} = \Omega_{ff} \sum_z \left[\sum_{e \in z} \left(\sum_{j \in e} \mathrm{A}_{nj} \left(\frac{1}{I_e} - \beta S_e (t_e - t_{e-1}) \right) \right) \right], \tag{A16}$$

where z goes over contact groups, e goes over all infection events (after the initially infected index case), and j sums over all infected individuals immediately prior to infection event e.

The time difference between consecutive events is exponentially distributed (because events are Markovian) according to

$$t_e - t_{e-1} = \frac{u_e}{\beta \left(\sum_{s \in e} e^{a_{g,s} + \varepsilon_{g,s}} \right) \left(\sum_{i \in e} e^{a_{f,i} + \varepsilon_{f,i}} \right)}, \tag{A17}$$

where s goes over all susceptible individuals immediate prior to time t_e and i goes over all infected individuals. The random variables u_e are exponentially distributed with a mean of one. Substituting Eq.(A17) into Eq.(A16), as well as assuming that phenotypic effects are small, leads to

$$\mu_{f,n} = \Omega_{ff} \sum_z \left[\sum_{e=1 \in z}^{N-1} \left(\sum_{j \in e} \mathrm{A}_{nj} \left(\frac{1}{e} - \frac{u_e}{e} \left[1 - \frac{1}{N-e} \sum_{s \in e} \left(a_{g,s} + \varepsilon_{g,s} \right) - \frac{1}{e} \sum_{i \in e} \left(a_{f,i} + \varepsilon_{f,i} \right) \right] \right) \right) \right], \tag{A18}$$

Note, here events have been numbered such that e=1 corresponds to the first infection in group z after the index case all the way up to e=N-1, where N is the number of individuals within each contact group. This expression assumes a simple SI model dynamics for the system, *i.e.* recoveries are ignored. This is justified by the fact that most of the information regarding an individual's infectivity comes from when it has just become infected (considering the begin of an epidemic the index case definitely infects the second individual, then one of the first two cases infects the third etc... successively less information in available regarding who is infecting whom).

Expressions for PAs

We now estimate the susceptibility and infectivity PAs for a given individual n. This is defined by the correlation between its estimated and true value:

$$\alpha_{g,n} = \frac{\langle \mu_{g,n} a_{g,n} \rangle - \langle \mu_{g,n} \rangle \langle a_{g,n} \rangle}{\sqrt{\left(\langle a_{g,n}^2 \rangle - \langle a_{g,n} \rangle^2 \right) \left(\langle \mu_{g,n}^2 \rangle - \langle \mu_{g,n} \rangle^2 \right)}},$$

$$\alpha_{f,n} = \frac{\langle \mu_{f,n} a_{g,n} \rangle - \langle \mu_{f,n} \rangle \langle a_{f,n} \rangle}{\sqrt{\left(\langle a_{f,n}^2 \rangle - \langle a_{f,n} \rangle^2 \right) \left(\langle \mu_{f,n}^2 \rangle - \langle \mu_{f,n} \rangle^2 \right)}}. \tag{A19}$$

where the angular brackets represent averages over potential realisations from the system (*e.g.*, this is equivalent to simulating from the system a large number of times and taking the average over those simulations). Note, unlike alternative definitions which rely of averages taken over the entire population (or sub-population, such as sires), this allows us to generate PAs for each individual (which makes sense because those individuals with more relatives are necessarily going to have higher PAs).

The means of quantities are zero $\langle a_{g,n}\rangle = \langle a_{f,n}\rangle = \langle \mu_{g,n}\rangle = \langle \mu_{f,n}\rangle = 0$ and the variances of the additive genetic effects are given by the covariance matrix $\mathbf{\Omega}$. Consequently Eq.(A19) simplifies to

$$\alpha_{g,n} = \frac{\langle \mu_{g,n} a_{g,n}\rangle}{\sqrt{\Omega_{gg}\langle \mu_{g,n}^2\rangle}}, \qquad \alpha_{f,n} = \frac{\langle \mu_{f,n} a_{f,n}\rangle}{\sqrt{\Omega_{ff}\langle \mu_{f,n}^2\rangle}}. \tag{A20}$$

Based on the expressions in Eq.(A15) and Eq.(A18), the follow quantities can be identified:

$$\begin{aligned}
\langle \mu_{g,n} a_{g,n}\rangle &= \Omega_{gg}\sum_z\Big[\sum_{j\in z} \mathrm{A}_{nj}^2\Big],\\
\langle \mu_{f,n} a_{f,n}\rangle &= \Omega_{ff}\sum_z\Big[\tfrac{1}{N}\sum_{j,q\in z} \mathrm{A}_{nj}\mathrm{A}_{nq}\nu_{jq}\Big],\\
\langle \mu_{g,n}^2\rangle &= \sum_z\Big[\sum_{j\in z}\mathrm{A}_{nj}^2\Big] + \sum_{z,z'}\Big[\sum_{j\in z,j'\in z'}\mathrm{A}_{nj}\mathrm{A}_{nj'}\Big(\Omega_{gg}\mathrm{A}_{jj'} + \Psi_{gg}\mathrm{I}_{jj'}\\
&\qquad + \tfrac{1}{N^2}\sum_{q\in z,q'\in z'}(\Omega_{ff}\mathrm{A}_{qq'} + \Psi_{ff}\mathrm{I}_{qq'})\Big)\Big],\\
\langle \mu_{f,n}^2\rangle &= \sum_z\Big[\tfrac{1}{N}\sum_{j\in z}\nu_{jj}\mathrm{A}_{nj}^2\Big] + \sum_{z,z'}\Big[\tfrac{1}{N^2}\sum_{j,q\in z,j',q'\in z'}\mathrm{A}_{nj}\mathrm{A}_{nj'}\\
&\qquad \times\Big(\omega_{jqj'q'}\big(\Omega_{gg}\mathrm{A}_{qq'} + \Psi_{gg}\mathrm{I}_{qq'}\big) + \eta_{jqj'q'}\big(\Omega_{ff}\mathrm{A}_{qq'} + \Psi_{ff}\mathrm{I}_{qq'}\big)\Big)\Big].
\end{aligned} \tag{A21}$$

Here z sums over contact groups and j and q sum over all individuals within z. Similarly z' sums over contact groups and j' and q' sum over all individuals within z'. Finally, $\mathbf{A}$ is the relationship matrix and $\mathbf{I}$ is the identity matrix.

These expression involve the following tensors:

$$\begin{aligned}
\tau_{jq} &= \Big\langle \sum_{e=E_j}^{E_q-1}\frac{N}{e(N-e)}\Big\rangle, \qquad \nu_{jq} = \Big\langle \sum_{e=\max(E_j,E_q)}^{N-1}\frac{N}{e^2}\Big\rangle,\\
\omega_{jqj'q'} &= \Big\langle \Big(\sum_{e=E_j}^{E_q-1}\frac{N}{e(N-e)}\Big)\Big(\sum_{e'=E_{j'}}^{E_{q'}-1}\frac{N}{e'(N-e')}\Big)\Big\rangle,\\
\eta_{jqj'q'} &= \Big\langle \Big(\sum_{e=\max(E_j,E_q)}^{N-1}\frac{N}{e^2}\Big)\Big(\sum_{e'=\max(E_{j'},E_{q'})}^{N-1}\frac{N}{e'^2}\Big)\Big\rangle,
\end{aligned} \tag{A22}$$

where E_j is the value of e for which individual j becomes infected and the angle brackets average over random orderings for E_j.

To make progress the expressions in Eq.(A22) are replaced by integral approximations. For example

$$
\begin{aligned}
\nu_{jj} &= \left\langle \sum_{e=E_j}^{N-1} \frac{N}{e^2} \right\rangle \cong \frac{1}{N-1} \int_{\frac{1}{2}}^{N-\frac{1}{2}} \int_{e=E_j}^{N} \frac{N}{e^2} de\, dE_j, \\
&\cong \frac{1}{N-1} \int_{\frac{1}{2}}^{N-\frac{1}{2}} \left(\frac{N}{E_j} - 1 \right) dE_j, \\
&\cong \frac{N\log(2N-1) - (N-1)}{N-1}, \\
&\cong \log(2N).
\end{aligned}
\tag{A23}
$$

The last line comes from assuming that N is reasonably large. Integral approximations for all the other quantities in Eq.(A22) yield the following results:

$$
\begin{aligned}
&\tau_{jq} = \begin{cases} 1 & \text{if } j \neq q \\ 0 & \text{if } j = q \end{cases}, \quad \nu_{jq} = \begin{cases} 1 & \text{if } j \neq q \\ \log(2N) & \text{if } j = q \end{cases}, \\
&\omega_{jqj'q'} = \begin{cases} \tau_{jq}\tau_{j'q'} & \text{if } j \text{ and } q \text{ different from } j' \text{ and } q' \\ 3 & \text{if } j = j' \neq q = q' \\ 2 & \text{if } j = j' \neq q \neq q' \text{ or } q = q' \neq j \neq j' \\ 0 & \text{if } j = q' \neq j' = q \end{cases} \\
&\eta_{jqj'q'} = \begin{cases} \nu_{jq}\nu_{j'q'} & \text{if } j \text{ and } q \text{ different from } j' \text{ and } q' \\ 3.5 & \text{if } j = q = j' = q' \\ 2 & \begin{array}{l} \text{if } j = j' \neq q \neq q' \text{ or } j = q' \neq q \neq j' \\ \text{or } q = j' \neq j \neq q' \text{ or } q = q' \neq j \neq j' \end{array} \\ \log^2(N) & \begin{array}{l} \text{if } j \neq j' = q = q' \text{ or } j' \neq q = q' = j \\ \text{or } q \neq j = j' = q' \text{ or } q' \neq q = j = j' \end{array} \\ 2\log(N) & \text{if } j = j' \neq q = q' \text{ or } j = q' \neq q = j' \end{cases}
\end{aligned}
\tag{A24}
$$

Substituting the expressions from Eq.(A21) into Eq.(A20) gives the final results:

$$
\begin{aligned}
\alpha_{g,n} &= \frac{\sqrt{\Omega_{gg}} \sum_s \mathrm{A}_{ns}^2}{\sqrt{\begin{array}{r} \sum_s \mathrm{A}_{ns}^2 + \sum_{z,z'} \Big[\sum_{j\in z, j'\in z'} \mathrm{A}_{nj}\mathrm{A}_{nj'} \big(\Omega_{gg}\mathrm{A}_{jj'} + \Psi_{gg}\mathrm{I}_{jj'} \\ + \frac{1}{N^2} \sum_{q\in z, q'\in z'} (\Omega_{ff}\mathrm{A}_{qq'} + \Psi_{ff}\mathrm{I}_{qq'}) \big) \Big] \end{array}}}, \\
\alpha_{f,n} &= \frac{\sqrt{\Omega_{ff}} \left(\frac{\log(2N)}{N} \sum_m \mathrm{A}_{nm}^2 + \sum_z \left[\frac{1}{N} \sum_{j\neq q\in z} \mathrm{A}_{nj}\mathrm{A}_{nq} \right] \right)}{\sqrt{\begin{array}{r} \frac{\log(2N)}{N} \sum_m \mathrm{A}_{nm}^2 + \sum_{z,z'} \Big[\frac{1}{N^2} \sum_{j,q\in z, j',q'\in z'} \mathrm{A}_{nj}\mathrm{A}_{nj'} \big(\omega_{jqj'q'} \big(\Omega_{gg}\mathrm{A}_{qq'} + \Psi_{gg}\mathrm{I}_{qq'} \big) \\ + \eta_{jqj'q'} \big(\Omega_{ff}\mathrm{A}_{qq'} + \Psi_{ff}\mathrm{I}_{qq'} \big) \big) \Big] \end{array}}}.
\end{aligned}
\tag{A25}
$$

To reiterate from above: z sums over contact groups and j and q sum over all individuals within z, z' also sums over contact groups and j' and q' sum over all individuals within z', and N is the number individuals within each contact group.

PA for recoverability

Substituting the part corresponding to the recovery process in the likelihood Eq.(4) and the prior in Additional file 2 Eq.(A2) into Eq.(A1) leads to the posterior probability

$$\pi(\theta\,|\,\xi) \propto \left(\prod_p F_\Gamma(\delta t_p\,|\,w_p,k)\right) \times e^{-\frac{1}{2\Omega_{rr}}\sum_{m,n} a_{r,m}\mathrm{A}^{-1}_{mn}a_{r,n} - \frac{1}{2\Psi_{rr}}\sum_m \varepsilon^2_{r,m}}, \tag{A26}$$

where p sums over all individual that get infected (including the index cases), and m and n sum over all individuals (whether they take part in epidemics or not). The term

$$F_\Gamma(\delta t_p\,|\,w_p,k) = \Gamma(k)^{-1} w_p^{-k} k^k \delta t_p^{k-1} e^{-k\delta t_p/w_p} \tag{A27}$$

is the gamma distribution, assuming that individuals have a mean infection duration w_p, as defined in Eq.(1). Substituting Eq.(1) into Eq.(A27) and taking the logarithm gives

$$\begin{aligned} l = \log\left[\pi(\theta\,|\,\xi)\right] = \sum_p k\left(a_{r,p} + \varepsilon_{r,p}\right) + (k-1)\log(\delta t_p) - k\gamma\delta t_p e^{a_{r,p}+\varepsilon_{r,p}} \\ - \tfrac{1}{2\Omega_{rr}}\sum_{m,n} a_{r,m}\mathrm{A}^{-1}_{mn}a_{r,n} - \tfrac{1}{2\Psi_{rr}}\sum_m \varepsilon^2_m. \end{aligned} \tag{A28}$$

The partial derivatives of this are

$$\frac{\partial l}{\partial a_{g,m}} = k\left(1 - \gamma\delta t_m e^{a_{r,m}+\varepsilon_{r,p}}\right) - \tfrac{1}{\Omega_{rr}}\sum_n \mathrm{A}^{-1}_{mn} a_{r,n} \tag{A29}$$

.

This analytical treatment estimates the mean posterior values for the additive genetic effects for the recoverability $\boldsymbol{\mu}_r$ by the values that $\boldsymbol{a}_r$ take in order to maximise the posterior probability distribution in Eq.(A28). This maximum is calculated by setting the derivatives in Eq.(A29) to zero and solving. In the general case this set of equations is intractable and to make further progress we take the limit in which $\boldsymbol{a}_r$ and $\boldsymbol{\varepsilon}_r$, are assumed to be small. Taylor expanding Eq.(A29) in these quantities we find that to first order the terms involving the relationship matrix dominate. This gives

$$\mu_{r,n} = k\Omega_{rr}\sum_m\left[\mathrm{A}_{nm}\left(1-\gamma\delta t_m\right)\right]. \tag{A30}$$

The recovery times can be written as

$$\delta t_m = \frac{u_m}{\gamma e^{a_{r,m}+\varepsilon_{r,m}}}, \tag{A31}$$

where u_m is a gamma distributed variable with mean 1. Substituting this into Eq.(A30) and making use of the fact that $a_{r,m}$ and $\varepsilon_{r,m}$ are small leads to

$$\mu_{r,n} = k\Omega_{rr}\sum_m\left[\mathrm{A}_{nm}\left(1-\left[1-a_{r,m}-\varepsilon_{r,m}\right]u_m\right)\right]. \tag{A32}$$

The following quantities can be derived from this equation:

$$\left\langle \mu_{r,n} a_{r,n} \right\rangle = \Omega_{rr} \sum_m \mathrm{A}_{nm}^2,$$
$$\left\langle \mu_{r,n}^2 \right\rangle = \tfrac{1}{k} \sum_m \mathrm{A}_{nm}^2 + \sum_{mq} \mathrm{A}_{nm} \mathrm{A}_{nq} \left(\Omega_{rr} \mathrm{A}_{mq} + \Psi_{rr} \mathrm{I}_{mq} \right), \tag{A33}$$

where $\mathbf{A}$ is the relationship matrix, $\mathbf{I}$ is the identity matrix and k is the shape parameter. From these quantities the PA can be derived:

$$\alpha_{r,n} = \frac{\left\langle \mu_{r,n} a_{r,n} \right\rangle}{\sqrt{\Omega_{rr} \left\langle \mu_{r,n}^2 \right\rangle}} = \frac{\sqrt{\Omega_{rr}} \sum_m \mathrm{A}_{nm}^2}{\sqrt{\tfrac{1}{k} \sum_m \mathrm{A}_{nm}^2 + \sum_{mq} \mathrm{A}_{nm} \mathrm{A}_{nq} \left(\Omega_{rr} \mathrm{A}_{mq} + \Psi_{rr} \mathrm{I}_{mq} \right)}}. \tag{A34}$$
