# Supplementary material for: SIRE 2.0: a novel method for estimating polygenic host effects underlying infectious disease transmission, and analytical expressions for prediction accuracies
Source: Genet Sel Evol. 2025 Apr 1;57:17. doi: 10.1186/s12711-025-00956-4 (PMC11963337; doi:10.1186/s12711-025-00956-4)
Supplement: Supplementary file 7 — Additional file 7. Simulating epidemics. How simulated data is generated using the modified Doob-Gillespie algorithm. [file 12711_2025_956_MOESM7_ESM.pdf]

Simulating epidemics

When simulating from the genetic-epidemiological model, firstly the additive genetic contributions $\boldsymbol{a}=(\boldsymbol{a}_g, \boldsymbol{a}_f, \boldsymbol{a}_r)$ and residual contributions $\varepsilon=(\varepsilon_g, \varepsilon_f, \varepsilon_r)$ are sampled from multivariate normal distributions with covariance matrices $\mathbf{A}\otimes\mathbf{\Omega}$ and $\mathbf{I}\otimes\mathbf{\Psi}$, respectively. This then allows for calculation of the underlying trait values in Eq.(1). The next stage of simulation involves sampling infection and recovery times from the SIR (or SI) compartmental model, as described below.

The Doob-Gillespie algorithm provides a means of taking into account inherent stochasticity in Markovian compartmental models (*i.e.*, models for which the transition rates depend solely on the current state of the system) [44]. The model used in this paper combines Markovian infection transitions with more realistic non-Markovian recovery dynamics. Below we describe how these recovery events are incorporated into the standard Doob-Gillespie framework.

The purpose of this procedure is to build up a time-ordered sequence of infection and recovery event times indexed by event number e. The following notation is used: t_e is the event time, x_e is the event type (infection "inf." or recovery "rec."), j_e is the affected individual, and t_j^I and t_j^R are the infection and recovery times for individual j, respectively.

Initialization: An epidemic in each contact group is assumed to be started by one initially infected index case j at some initial time point t_0. The infection duration δt_j for this individual is drawn from a gamma distribution parameterised in terms of an individual-based mean and shape parameter:

$$\delta t_j \sim \text{Gamma}(w_j, k) \tag{A1}$$

(note, the dependency of w_j on θ is given through Eqs. (1) and (2)). This allows us to set $t_j^I = t_0$ and $t_j^R = t_0 + \delta t_j$. Individual j is then placed onto a list $\mathcal{R}$, which represents all currently infected individuals. We set event index to $e=1$.

Step 1: Calculate the time to the next infection event. This is done by first evaluating the total transition rate that any individual becomes infected

$$\Lambda = \sum_s \lambda_s, \tag{A2}$$

where the sum s goes over all currently susceptible individuals and the force of infection λ_s (which gives the probability per unit time of s becoming infected) is given by Eq.(1). In accordance with a Poisson process, the time to the next infection event is generated by drawing a sample from the exponential distribution $\Lambda e^{-\Lambda\Delta t}$. In practice, this is achieved by selecting an inter-event time using

$$\Delta t = -\frac{\log(u)}{\Lambda}, \tag{A3}$$

where u is a (uniform) randomly generated number between 0 and 1. The new event time is then defined by

$$t^{new} = t_{e-1} + \Delta t. \tag{A4}$$

Step 2: Choosing the event type. For the SIR model two possibilities exist:

a) If t^{new} is greater than the smallest recovery time of all the individuals in $\mathcal{R}$, which we label j_{min}, then we remove $j_{\min}$ from $\mathcal{R}$ and set

$$t_e = t^R_{j_{\min}}, \;\; x_e = \mathrm{rec.}, \;\; j_e = j_{\min}. \tag{A5}$$

b) Otherwise, we set

$$t_e = t^{new}, \;\; x_e = \inf., \tag{A6}$$

and select the individual that becomes infected with probability

$$\mathrm{Prob}(j_e = s) = \frac{\lambda_s}{\Lambda}. \tag{A7}$$

The infection duration δt_{j_e} for j_e is sampled using Eq.(A1), and the infection and recovery times are set to

$$\begin{aligned} t^I_{j_e} &= t_e, \\ t^R_{j_e} &= t_e + \delta t_{j_e}. \end{aligned} \tag{A8}$$

Individual j_e is then placed onto the list $\mathcal{R}$.

Step 3: If there are any remaining infected individuals, increment e and jump to step 1.

End: Insert recovery times for any remaining individuals j in $\mathcal{R}$

$$t_e = t^R_j, \;\; x_e = \mathrm{rec.}, \;\; j_e = j, \tag{A9}$$

incrementing e after each addition.

The above algorithm describes simulation of a single contact group. The procedure is repeated separately for each contact group (which is valid because the infection dynamics of groups are assumed to be closed) to generate a complete set of infection and recovery events ξ for the entire population of individuals.
