# Supplementary material for: SIRE 2.0: a novel method for estimating polygenic host effects underlying infectious disease transmission, and analytical expressions for prediction accuracies
Source: Genet Sel Evol. 2025 Apr 1;57:17. doi: 10.1186/s12711-025-00956-4 (PMC11963337; doi:10.1186/s12711-025-00956-4)
Supplement: Supplementary file 8 — Additional file 8. Extension to other population structures. Looks at how PAs change under inclusion of full-sibs or related sires. [file 12711_2025_956_MOESM8_ESM.pdf]

Extension to other population structures

The models introduced so far used a simple population structure in which unrelated sires randomly mate with unrelated dams to produce paternal half-sib offspring. Here we look at how PAs change under different scenarios:

Inclusion of full-sibs – Here each sire mates with a certain number of dams to produce (at least, some) full-sib offspring. Results are shown in Fig. A(a) for fixed overall number of offspring P=24 (using 100 sires, Z=240 contact groups and 2400 progeny in total). Although not a strong dependency, we find that PAs are highest when each sire mates with many dams, each resulting in a single offspring (right-hand side of the diagram) but reduces until the sire mates with just a single dam (on the left-hand side) to produce a full-sib family. The reason for this is that in the latter case inference is unable to distinguish whether any systematic feature of the offspring (*e.g.* they are highly susceptible or infectious) is attributable to the sire or dam.

Related sires – Rather than considering just two generations[1], we consider the case of multiple generations, each of which contains a population of 2400 individuals. Within each generation 100 sires are randomly selected and mated with P=24 randomly selected dams to produce the next generation. The last generation are randomly divided into contact groups that undergo epidemics from which the infection and recovery times are used as data. PAs for the sires in the last-but-one generation are shown in Fig. A(b), where the total number of generations is varied. Note, the case of 2 generations correspond to the scenario previously looked at. However as more generations are added, so the sires (whose PAs are being measured) become increasingly related. We find that this results in a modest reduction in PAs.

The trends shown in Fig. A agree well with those predicted by the analytical dashed line in Eq.(6).

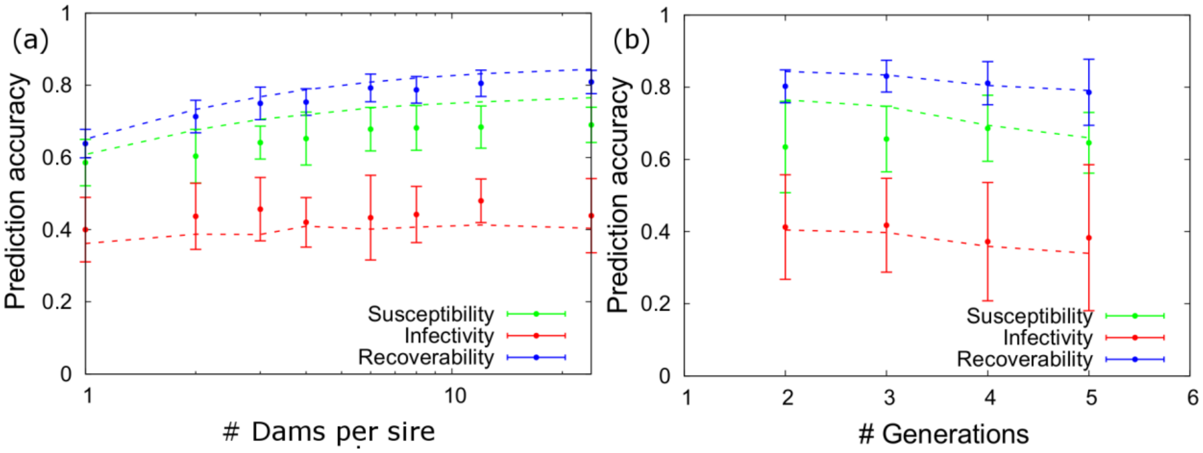

Figure A. Different population structures. These plots show how sire PAs for the susceptibility (green), infectivity (red) and recoverability (blue) vary with (a) the number of dams per sire (P=24, N=10, Z=200 contact groups) and (b) the number of generations (P=24, 2400 individuals per generation). The circles with error bars give the mean and standard deviation of numerical estimates obtained from 20 simulated datasets. The analytical dashed lines come from Eq.(6).

[1] Sires and dams in the first generation and progeny in the second.
