# Supplementary material for: SIRE 2.0: a novel method for estimating polygenic host effects underlying infectious disease transmission, and analytical expressions for prediction accuracies
Source: Genet Sel Evol. 2025 Apr 1;57:17. doi: 10.1186/s12711-025-00956-4 (PMC11963337; doi:10.1186/s12711-025-00956-4)
Supplement: Supplementary file 9 — Additional file 9. Interpreting phenotypic variances and modelling super-spreaders. This section aims to interpret the values used for phenotypic variances and introduces the 80/20 rule. [file 12711_2025_956_MOESM9_ESM.pdf]

Interpreting phenotypic variances and modelling super-spreaders

This section aims to interpret the values used for phenotypic variances. When variances are small, the square root in their value (*i.e.*, the phenotypic standard deviation) gives the expected fractional change in the trait. So Θ_{gg}=0.1 corresponds to a fractional variation in susceptibility of √0.1=0.32. In other words, individuals will be roughly 32% more or less susceptible, randomly distributed around a population average. As variances become larger and larger, however, this simple interpretation breaks down. Figure A looks at the case of infectivity. Here we plot the fraction of infections caused by the top 20% most infectious individuals at any given point in time against phenotypic variance in infectivity Θ_{ff}. This curve assumes a large number of individuals and is generated by numerically evaluating

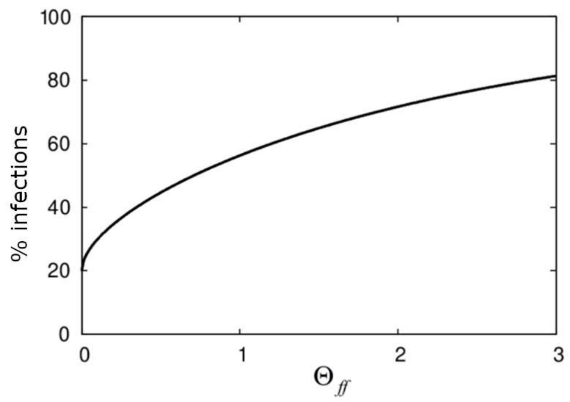

Figure A: Percentage of infections caused by the 20% most infectious individuals as a function of the phenotypic variance in infectivity Θ_{ff}.

$$\int_{x_{80}}^{\infty} N(x\,|\,0,\Theta_{ff})e^{x}dx \Big/ \int_{-\infty}^{\infty} N(x\,|\,0,\Theta_{ff})e^{x}dx \qquad (1)$$

where $N(x|0,\Theta_{ff})$ is a normal distribution with zero mean and variance Θ_{ff}. The value x_{80} is the 80th percentile of this distribution.

When the phenotypic variance is small, individuals have almost identical infectivity, hence the curve in Fig. A goes through 20% on the left-hand edge. On the other extreme, the right-hand edge corresponds to some individuals being vastly more infectious than others (by several orders of magnitude). Uncommonly infectious individual, otherwise known as “super-spreaders”, are an established feature in many epidemiological settings. Some cases of super-spreading conform to the 80/20 rule [75], where approximately 20% of the infected individuals are responsible for 80% of transmissions. By inspecting Fig. A we find that this corresponds to a phenotypic variance of Θ_{ff} ≈2.8. This value is used in the exemplar model (Table 2).
