# Supplementary material for: SIRE 2.0: a novel method for estimating polygenic host effects underlying infectious disease transmission, and analytical expressions for prediction accuracies
Source: Genet Sel Evol. 2025 Apr 1;57:17. doi: 10.1186/s12711-025-00956-4 (PMC11963337; doi:10.1186/s12711-025-00956-4)
Supplement: Supplementary file 10 — Additional file 10. Expression of prediction accuracies for a simple paternal half-sib model. Here the complex prediction accuracies are simplified by assuming a paternal half-sib model. [file 12711_2025_956_MOESM10_ESM.pdf]

Expression of prediction accuracies for a simple paternal half-sib model

This section derives concrete expressions for sire prediction accuracies (PAs) assuming the following simple population structure: Each unrelated sire is randomly mated with P unrelated dams to generate P offspring. These offspring are separated into groupings of size c which are randomly allocated across contact groups. Epidemics are then instigated in each contact group and the infection and recovery time data is used to inform the susceptibility, infectivity and recoverability phenotypes for the sire population.

Using the analytic expressions in Additional file 6 Eqs.(A21) and (A33) arbitrarily selecting individual n to be one of the sires in the population, the following quantities can be derived:

$$
\begin{aligned}
&\left\langle \mu_{g,SIRE} a_{g,SIRE} \right\rangle = \tfrac{P'}{4}\Omega_{gg},\\
&\left\langle \mu_{f,SIRE} a_{f,SIRE} \right\rangle = \tfrac{P}{4}\tfrac{\log(2N)+c-1}{N}\Omega_{ff},\\
&\left\langle \mu_{r,SIRE} a_{r,SIRE} \right\rangle = \tfrac{P}{4}\Omega_{rr},\\
&\left\langle \mu_{g,SIRE}^2 \right\rangle = \tfrac{P'}{4} + \tfrac{P'}{16}\left((3+c)\Omega_{gg} + 4\Psi_{gg} + \tfrac{c(4N+c^2-c)}{N^2}\Omega_{ff} + \tfrac{4c}{N}\Psi_{ff}\right)\\
&\qquad + \tfrac{P'(P'-c)}{16}\left(\Omega_{gg} + \tfrac{c^2}{N^2}\Omega_{ff}\right),\\
&\left\langle \mu_{f,SIRE}^2 \right\rangle = \tfrac{P}{4N}\log(2N) + \tfrac{P}{4N^2}\left(\left[3N + \tfrac{1}{2}(c-1)N - (c-1)\right]\Omega_{gg} + 3(N-1)\Psi_{gg}\right)\\
&\qquad + \tfrac{P(c-1)}{4N^2}\left(\left[2(N-2) + \tfrac{1}{4}(c-1)N - \tfrac{1}{2}(c-1)\right]\Omega_{gg} + 2(N-2)\Psi_{gg}\right)\\
&\qquad + \tfrac{P(P-c)}{16N^2}(c-1)^2\Omega_{gg}\\
&\qquad + \tfrac{P}{4N^2}\left(\left[\tfrac{1}{2}(c-1)N - (c-1) + 3.5N + \tfrac{1}{2}\log^2(N) + 2(N-1)\log(N)\right]\Omega_{ff}\right)\\
&\qquad + \tfrac{P}{4N^2}\left(\left[3.5N + 2(N-1)\log(N)\right]\Psi_{ff}\right)\\
&\qquad + \tfrac{P(c-1)}{4N^2}\left(\left[2(N-2) + \tfrac{1}{4}(c-1)N - \tfrac{1}{4}c + 2\log^2(N) + \tfrac{1}{2}\log(N) + \tfrac{1}{4}\log^2(2N)\right]\Omega_{ff}\right)\\
&\qquad + \tfrac{P(c-1)}{4N^2}\left(\left[2(N-2) + 2\log^2(N)\right]\Psi_{ff}\right)\\
&\qquad + \tfrac{P(P-c)}{16N^2}\left([c-1]^2 + 2(c-1)\log(2N) + \log^2(2N)\right)\Omega_{ff},\\
&\left\langle \mu_{r,SIRE}^2 \right\rangle = \tfrac{P}{4k} + \tfrac{P}{4}\Theta_{rr} + \tfrac{P(P-1)}{16}\Omega_{rr}.
\end{aligned}
\tag{A1}
$$

Here, the modified number of offspring $P'=PN/(N-1)$ takes into account the fact that on average some offspring are index cases which provide no information regarding their susceptibility.

A special case is when c=1, *i.e.* each offspring from the sire are placed into different contact groups. Here, the expressions from Eq.(A1) simplifies somewhat:

$$\begin{aligned}
&\left\langle \mu_{g,SIRE} a_{g,SIRE} \right\rangle = \tfrac{P}{4}\Omega_{gg}, \\
&\left\langle \mu_{f,SIRE} a_{f,SIRE} \right\rangle = \tfrac{P}{4}\tfrac{\log(2N)}{N}\Omega_{ff}, \\
&\left\langle \mu_{r,SIRE} a_{r,SIRE} \right\rangle = \tfrac{P}{4}\Omega_{rr}, \\
&\left\langle \mu^2_{g,SIRE} \right\rangle = \tfrac{P}{4}\left[1+\tfrac{P}{4}\Omega_{gg}+\Theta_{gg}+\tfrac{1}{N}\Theta_{ff}\right], \\
&\left\langle \mu^2_{f,SIRE} \right\rangle = \tfrac{P}{4N}\left[\log(2N)+3\Theta_{gg}+2\log(N)\Theta_{ff}+\tfrac{P^2}{4N}\log^2(2N)\Omega_{ff}\right], \\
&\left\langle \mu^2_{r,SIRE} \right\rangle = \tfrac{P}{4}\left[\tfrac{1}{k}+\Theta_{rr}+\tfrac{P}{4}\Omega_{rr}\right],
\end{aligned} \qquad \text{(A2)}$$

where it is assumed that N and P are reasonably large such that only dominant terms in N are kept[1] and P-1 can be approximated by simply P. Substituting these results into Additional file 6 Eqs.(A20) and (A34) gives the final expressions:

$$\begin{aligned}
\alpha_{g,SIRE} &= \left[1+\tfrac{4N}{P\Omega_{gg}(N-1)}\left(1+\Theta_{gg}+\tfrac{1}{N}\Theta_{ff}\right)\right]^{-\frac{1}{2}}, \\
\alpha_{f,SIRE} &= \left[1+\tfrac{4N}{P\Omega_{ff}\log(2N)}\left(1+2\Theta_{ff}+\tfrac{3}{\log(2N)}\Theta_{gg}\right)\right]^{-\frac{1}{2}}, \\
\alpha_{r,SIRE} &= \left[1+\tfrac{4}{P\Omega_{rr}}\left(\tfrac{1}{k}+\Theta_{rr}\right)\right]^{-\frac{1}{2}}.
\end{aligned} \qquad \text{(A3)}$$

In the more general case in which offspring are not placed into separate epidemic groups, a similar set of approximations leads to

$$\begin{aligned}
\alpha_{g,SIRE} &= \left[1+\tfrac{4N}{P\Omega_{gg}(N-1)}\left(1+\Theta_{gg}+\tfrac{1}{n_S}\Theta_{ff}\right)+\tfrac{1}{n_s^2}\tfrac{\Omega_{ff}}{\Omega_{gg}}\right]^{-\frac{1}{2}}, \\
\alpha_{r,SIRE} &= \left[1+\tfrac{4}{P\Omega_{rr}}\left(\tfrac{1}{k}+\Theta_{rr}\right)\right]^{-\frac{1}{2}},
\end{aligned} \qquad \text{(A4)}$$

where $n_s=N/c$ is the number of sires used to generate individuals for a particular contact group (*e.g.* n_s=1 implies that all individuals in a given contact group are paternal half-sibs). Comparing (A4) with (A3) shows that if half-sibs are preferentially selected to share the same contact groups then $\alpha_{g,SIRE}$ decreases and $\alpha_{r,SIRE}$ stays the same. A compact expression for $\alpha_{f,SIRE}$ was found to not be possible.

[1] *E.g.* Terms of order $N\log(N)$ dominate over terms of order N, so the latter are ignored.
