# Supplementary material for: SIRE 2.0: a novel method for estimating polygenic host effects underlying infectious disease transmission, and analytical expressions for prediction accuracies
Source: Genet Sel Evol. 2025 Apr 1;57:17. doi: 10.1186/s12711-025-00956-4 (PMC11963337; doi:10.1186/s12711-025-00956-4)
Supplement: Supplementary file 11 — Additional file 11. The incorporation of random group or fixed effects. Investigates the impact of random group or fixed effects on prediction accuracies for the epidemiological host traits. [file 12711_2025_956_MOESM11_ESM.pdf]

The incorporation of random group or fixed effects

This section investigates the impact of random group or fixed effects on prediction accuracies for the epidemiological host traits. Because these impact only the infection process, the results focus on only susceptibility and infectivity PAs (recoverability results do not change).

Figure A(a) shows how PAs depend on the size of the group effect σ_c (*i.e.* the standard deviation across contact groups in the contribution c_z in Eq.(1)). We find that variation in this quantity has almost no effect on the prediction accuracies. The left-hand side of the graph shows the case in which group effect is removed from the model altogether, again resulting in no appreciable change to the PAs.

Figure A(b) shows the case in which a single fixed effect was incorporated into the model, as follows: in Eq.(2) the elements for the design matrix $\mathbf{X}$ (which is actually a vector rather than a matrix in this particular case) were randomly assigned a value of ½ or -½ for each individual (*e.g.* representing a sex difference in trait value) and the values for the fixed effects $b_{g,1}$ and $b_{f,1}$ were randomly drawn from a normal distribution with standard deviation σ_{FE}. Figure A(b) shows how PAs depend on the effect size σ_{FE}. We find that even fixed effects of large size result in no appreciable change in PAs compared to the model with no fixed effect at all (on left-hand edge of this graph).

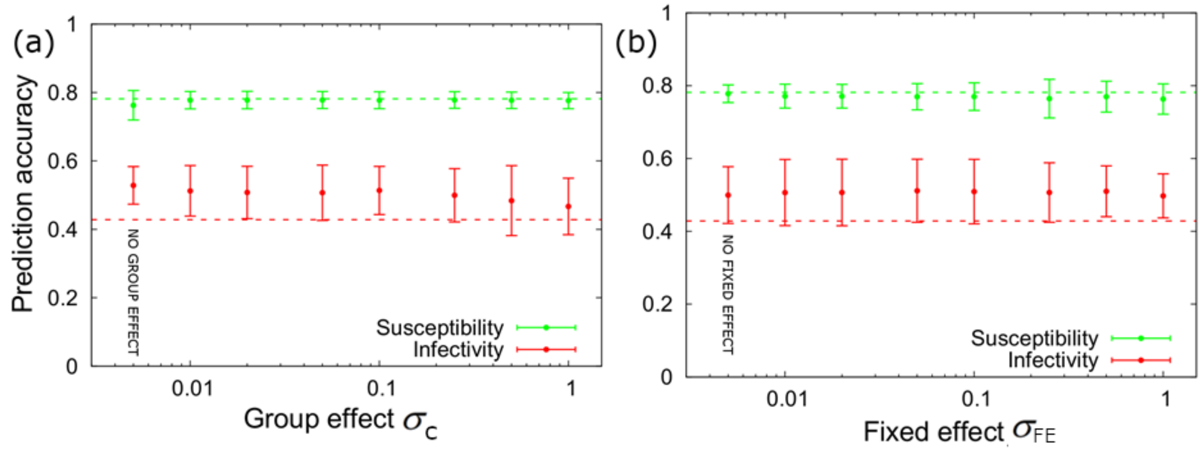

Figure A. Dependency of PAs on random group and fixed effects. These plots show how PAs for susceptibility (green) and infectivity (red) vary for (a) the size of the group effect, as measured by the standard deviation σ_c, and (b) the size of a fixed effect σ_{FE}. Results were generated from simulated data using the baseline scenario with known infection and recovery times (Table 2). The circles with error bars give the mean and standard deviation of numerical estimates obtained from 20 simulated datasets. The analytical dashed lines come from Eq.(6), which assumes no fixed or group effects.
